# Global Seasonal Activities of Respiratory Syncytial Virus Before the Coronavirus Disease 2019 Pandemic: A Systematic Review

**DOI:** 10.1093/ofid/ofae238

**Published:** 2024-04-25

**Authors:** Songwei Shan, Weixin Zhang, Huizhi Gao, Pei-Yu Huang, Zhanwei Du, Yuan Bai, Yiu-Chung Lau, Dongxuan Chen, Eric H Y Lau, Joshua Nealon, Peng Wu

**Affiliations:** World Health Organization Collaborating Centre for Infectious Disease Epidemiology and Control, School of Public Health, Li Ka Shing Faculty of Medicine, The University of Hong Kong, Hong Kong Special Administrative Region, China; Laboratory of Data Discovery for Health, Hong Kong Science and Technology Park, Hong Kong Special Administrative Region, China; World Health Organization Collaborating Centre for Infectious Disease Epidemiology and Control, School of Public Health, Li Ka Shing Faculty of Medicine, The University of Hong Kong, Hong Kong Special Administrative Region, China; World Health Organization Collaborating Centre for Infectious Disease Epidemiology and Control, School of Public Health, Li Ka Shing Faculty of Medicine, The University of Hong Kong, Hong Kong Special Administrative Region, China; World Health Organization Collaborating Centre for Infectious Disease Epidemiology and Control, School of Public Health, Li Ka Shing Faculty of Medicine, The University of Hong Kong, Hong Kong Special Administrative Region, China; World Health Organization Collaborating Centre for Infectious Disease Epidemiology and Control, School of Public Health, Li Ka Shing Faculty of Medicine, The University of Hong Kong, Hong Kong Special Administrative Region, China; Laboratory of Data Discovery for Health, Hong Kong Science and Technology Park, Hong Kong Special Administrative Region, China; World Health Organization Collaborating Centre for Infectious Disease Epidemiology and Control, School of Public Health, Li Ka Shing Faculty of Medicine, The University of Hong Kong, Hong Kong Special Administrative Region, China; Laboratory of Data Discovery for Health, Hong Kong Science and Technology Park, Hong Kong Special Administrative Region, China; World Health Organization Collaborating Centre for Infectious Disease Epidemiology and Control, School of Public Health, Li Ka Shing Faculty of Medicine, The University of Hong Kong, Hong Kong Special Administrative Region, China; Laboratory of Data Discovery for Health, Hong Kong Science and Technology Park, Hong Kong Special Administrative Region, China; World Health Organization Collaborating Centre for Infectious Disease Epidemiology and Control, School of Public Health, Li Ka Shing Faculty of Medicine, The University of Hong Kong, Hong Kong Special Administrative Region, China; Laboratory of Data Discovery for Health, Hong Kong Science and Technology Park, Hong Kong Special Administrative Region, China; World Health Organization Collaborating Centre for Infectious Disease Epidemiology and Control, School of Public Health, Li Ka Shing Faculty of Medicine, The University of Hong Kong, Hong Kong Special Administrative Region, China; Laboratory of Data Discovery for Health, Hong Kong Science and Technology Park, Hong Kong Special Administrative Region, China; World Health Organization Collaborating Centre for Infectious Disease Epidemiology and Control, School of Public Health, Li Ka Shing Faculty of Medicine, The University of Hong Kong, Hong Kong Special Administrative Region, China; World Health Organization Collaborating Centre for Infectious Disease Epidemiology and Control, School of Public Health, Li Ka Shing Faculty of Medicine, The University of Hong Kong, Hong Kong Special Administrative Region, China; Laboratory of Data Discovery for Health, Hong Kong Science and Technology Park, Hong Kong Special Administrative Region, China

**Keywords:** activity, global, respiratory syncytial virus, pre-pandemic, seasonality

## Abstract

Varied seasonal patterns of respiratory syncytial virus (RSV) have been reported worldwide. We conducted a systematic review on articles identified in PubMed reporting RSV seasonality based on data collected before 1 January 2020. RSV seasonal patterns were examined by geographic location, calendar month, analytic method, and meteorological factors including temperature and absolute humidity. Correlation and regression analyses were conducted to explore the relationship between RSV seasonality and study methods and characteristics of study locations. RSV seasons were reported in 209 articles published in 1973–2023 for 317 locations in 77 countries. Regular RSV seasons were similarly reported in countries in temperate regions, with highly variable seasons identified in subtropical and tropical countries. Longer durations of RSV seasons were associated with a higher daily average mean temperature and daily average mean absolute humidity. The global seasonal patterns of RSV provided important information for optimizing interventions against RSV infection.

## Teaser

Respiratory syncytial virus (RSV) seasons are largely regular in temperate regions but more variable in tropical/subtropical areas. Durations of RSV seasons are not only associated with the meteorological factors but also the methods used in the analysis.

Respiratory syncytial virus (RSV) is a leading cause of hospitalization for acute lower respiratory tract infection (LRTI) in young children <5 years old [1–4], as well as an increasingly recognized important pathogen in older adults (aged ≥65 years) [5, 6]. Globally, RSV was estimated to cause nearly 33.1 million LRTI episodes and >110 000 deaths every year [1]. In addition to palivizumab, a monoclonal antibody recommended for high-risk infants [7, 8] providing a short-term protection against RSV-associated severe infections [9, 10], a new monoclonal antibody, nirsevimab, with a prolonged protection [11, 12] has received approval in Europe in 2022, and subsequently in the United States (US) in 2023 [13]. More recently, prefusion F-protein–based RSV vaccines were approved by the US Food and Drug Administration and the European Medicines Agency for protecting older adults against severe RSV infection and for use in pregnant mothers to protect their infants against RSV-associated LRTIs [14–16].

Activities of RSV varied temporally and geographically. In temperate regions, the highest viral activities have been observed during winter months with similar intensities across different years [17–19]. Two-year cycles of RSV activity have been reported for some countries in Northern Europe, with a major epidemic wave in winter followed by a minor wave in spring the next year [20]. In subtropical and tropical regions, seasonal patterns of RSV were more diverse and less clear than those reported for temperate countries [18, 21, 22]. Weak latitudinal gradients in the peaking time of RSV circulation were reported, with peak timing occurring later with increasing latitude [17, 18]. Knowledge about the seasonality of RSV can guide the formulation of prevention strategies and the planning of public health resources. In particular, accurate estimates of the RSV season start and its duration can help to determine the optimal timing of initial doses and optimal dosing interval for prophylactic monoclonal antibodies and vaccination schedules.

We conducted a systematic review on RSV seasonality to characterize the global patterns of RSV activity by summarizing the reported start, peak, and end of RSV seasons worldwide and describe approaches applied in analyzing the seasonality. We aimed to provide a better understanding of the seasonal patterns of RSV circultion to inform policy and decisions in prevention, diagnosis, and treatment of RSV infections.

## METHODS

### Literature Search Strategy and Selection Criteria

**Figure 1.**
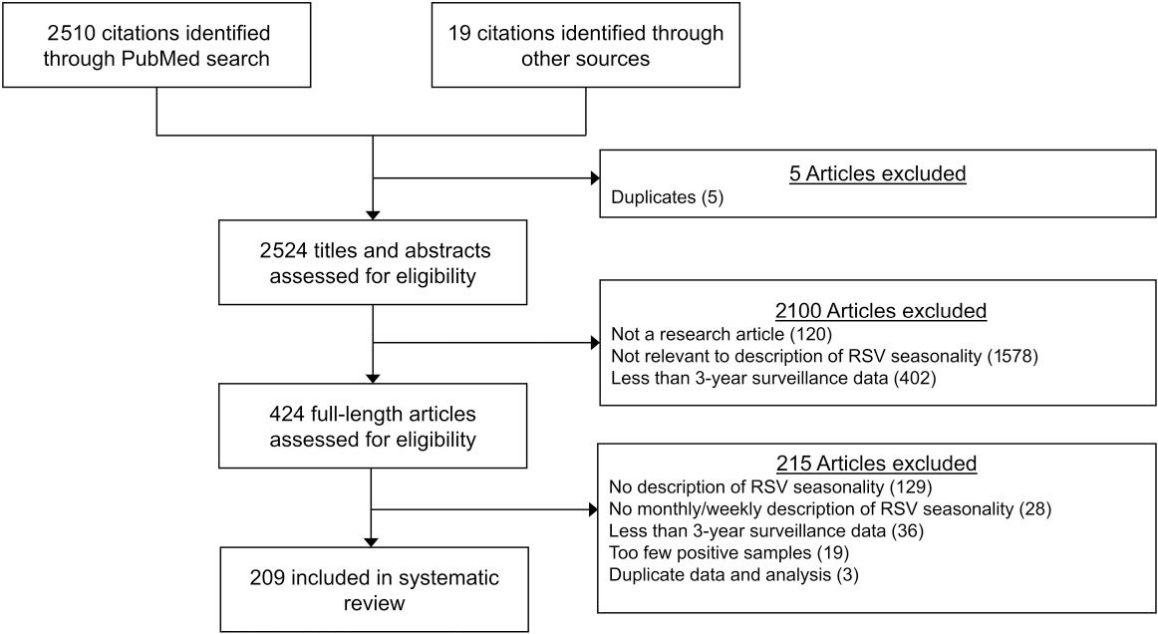
Flowchart of study identification and selection for the systematic review. Abbreviation: RSV, respiratory syncytial virus.

This systematic review was conducted following the Preferred Reporting Items for Systematic Reviews and Meta-Analyses (PRISMA) guidelines [23]. We focused on studies in which seasonal patterns of RSV were characterized by providing the time of the start, peak, and/or end of a season based on laboratory-confirmed RSV infection data. We searched published articles from PubMed on 17 January 2023 using the combination of following search items in “Title/Abstract” (Figure 1): (1) “RSV” or “respiratory syncytial virus”; (2) “seasonality” or “season*”; 2 authors (S. S. and W. Z.) performed the literature search and screened retrieved articles independently. Full-text screening was conducted after title/abstract screening. Two authors (S. S. and W. Z.) independently identified articles for inclusion. Any disagreement in article selection and data retrieval was resolved by a third author (P. W.).

Eligible articles were studies reporting the seasonality of RSV based on monthly or weekly laboratory-confirmed RSV infections collected for a specified place (ie, a city, a province, or a country) for 3 years or longer. We excluded articles if (1) the full text was not available or not in English; (2) the analysis was based on <3 years’ data; (3) the average number of reported RSV infections per year in a study was <50; or (4) the time of the start, peak, and/or end of RSV seasons was not specified in a calendar month or week. If an investigation on RSV seasonality covered the time period beyond 2019, the study period before the coronavirus disease 2019 (COVID-19) pandemic ≥3 years would be included in our analysis and the RSV data collected during the pandemic would be excluded. Articles without original data analysis on RSV activities, such as reviews, commentaries, and letters, were excluded, but we reviewed the references of these articles for additional studies to include for screening.

### Data Extraction and Analysis

Two authors (S. S. and W. Z.) independently extracted data into a standardized data extraction form. Data fields included the timing (start, peak, and end) of an RSV season, study location (city, region or country), study period, and information on study characteristics (eg, the source of cases, case definitions, laboratory testing methods, and analytic methods applied to determine RSV seasons). We classified the sources of study cases into outpatient, inpatient, and outpatient and inpatient; case definitions into influenza-like illness (ILI) or acute respiratory infection (ARI), severe ARI, and acute LRTI; and grouped laboratory testing methods for RSV confirmation into virus detection, antibody detection, antigen detection, and nucleic acid detection.

One publication might contain several different investigations on seasonal patterns of RSV, either based on different data sources or based on the same dataset but with different analytic methods. If >1 estimate on timing of RSV season was reported in a single publication, using different data sources or methods, all were extracted and included in our analyses. It was common that 1 investigation on RSV seasonality was conducted based on >1 type of case source, case definition, and/or testing method, and all the methodological details were extracted and documented for analyses. Analytic methods used by different studies to determine the RSV seasons were classified as qualitative methods and quantitative methods based on whether the analysis on RSV seasonality was carried out using a statistical or mathematical approach, and the quantitative methods were further divided into 3 groups: threshold-based, coverage-based, and model-based (Supplementary Materials).

In addition to the study-specific information, we also documented the latitude, daily average mean temperature, and daily average mean relative humidity for all study locations reported in each investigation using Google Geocoding API [24] and R package GSOR [25] (Supplementary Materials). The extracted daily average mean temperature and relative humidity data were used to derive the daily average mean absolute humidity for each location. We also extracted the geoclimatic information of each study site, and classified the investigations of RSV seasonality into 3 groups based on the latitude of the study sites: temperate zone (latitude >35° or <−35°), subtropical zone (23.5° < latitude ≤35° or −35° ≤ latitude <−23.5°), and tropical zone (−23.5° ≤ latitude ≤23.5°).

We described the reported RSV seasons as a function of geographical location, meteorological factors, and characteristics of study design, and explored potential associations using Pearson correlation and Kendall rank correlation analyses between the duration of the season and the latitude, daily average mean temperature and daily average mean absolute humidity of study sites, as well as the analytic method and climate zone. Finally, linear regression models were applied to explore the independent associations between the duration of RSV seasons and the abovementioned factors as covariates. The statistical analyses were conducted using R software version 4.0.3 (R Foundation for Statistical Computing, Vienna, Austria).

## RESULTS

We identified 2510 citations from PubMed through the keyword search and additional 19 citations from other sources. In total, 209 publications were included after screening based on the inclusion and exclusion criteria (Figure 1), and 552 investigations of RSV seasonality were identified and included in analyses.

**Figure 2.**
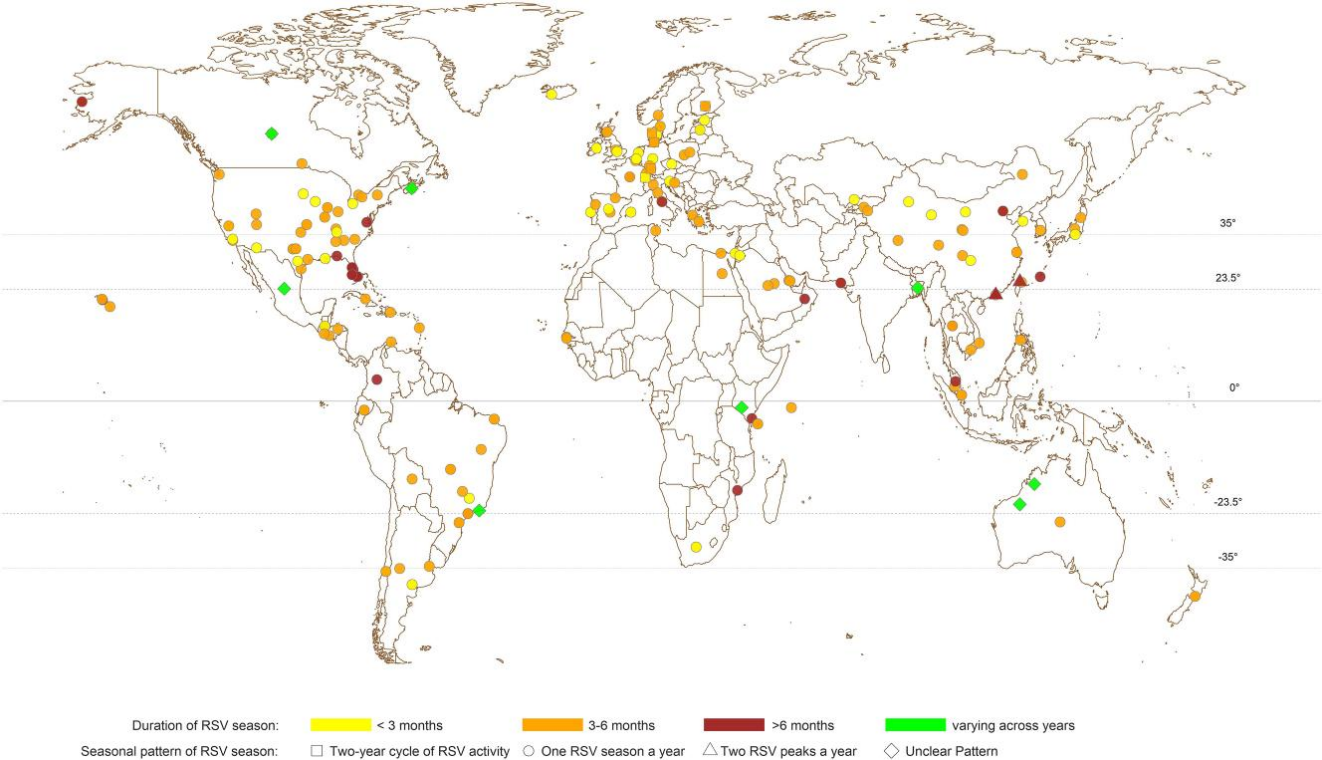
Geographic distribution of study locations and the reported respiratory syncytial virus (RSV) seasonal patterns from all included investigations using either quantitative or qualitative approaches.

The identified studies were published between 1973 and 2023, with the majority (147/209 [70%]) from 2010 onward (Supplementary Figure 1). RSV seasonality was reported for 317 distinct study sites in 77 countries from Asia, Europe, North America, South America, Oceania, and Africa (Figure 2). Most investigations (342/552 [62%]) were conducted with a study period of 3–6 years (Supplementary Figure 1), with the longest one from Malaysia using surveillance data over 27 years [26]. Among the identified publications, more than half (124/209 [59%]) were from temperate regions (latitude >35° or latitude <−35°), followed by 31% (65/209) and 22% (47/209) in subtropical (23.5° < latitude ≤35° or −35° ≤ latitude <−23.5°) and tropical (−23.5° ≤ latitude ≤23.5°) regions, respectively (Table 1, Supplementary Table 1).

**Table 1.** Characteristics of Publications and Investigations Included in the Systematic Review

Characteristic	No. (%) of Publications	No. (%) of Investigations
	(N = 209)^a^	(N = 552)
Climatic zone		
Temperate (>35°)	123 (58.9)	335 (60.7)
Subtropical (23.5°–35°)	65 (31.1)	134 (24.3)
Tropical (equator, 23.5°)	49 (23.4)	83 (15.0)
Continent		
Asia	64 (30.6)	140 (25.4)
Europe	68 (32.5)	155 (28.1)
North America	38 (18.2)	164 (29.7)
South America	14 (6.7)	49 (8.9)
Africa	12 (5.7)	24 (4.3)
Oceania	5 (2.4)	19 (3.4)
Others^b^	8 (3.8)	1 (0.2)
Study period^c^		
Before palivizumab approved	88 (42.1)	147 (26.6)
After palivizumab approved	75 (35.9)	320 (58.0)
Others^d^	46 (22.0)	85 (15.4)
Length of study period		
3–6 y	127 (60.8)	342 (62.0)
7–10 y	53 (25.4)	123 (22.3)
>10 y	37 (17.7)	87 (15.8)
Type of RSV data		
Weekly data	57 (27.3)	294 (53.3)
Monthly data	155 (74.2)	258 (46.7)
Analytic method		
Qualitative	146 (69.9)	179 (32.4)
Quantitative	66 (31.6)	373 (67.6)
Threshold-based	54 (25.9)	205 (37.1)
Coverage-based	8 (3.8)	142 (25.7)
Model-based	6 (2.9)	26 (4.7)
RSV case source		
Inpatient	94 (45.0)	152 (27.5)
Outpatient	14 (6.7)	25 (4.5)
Both	53 (25.4)	115 (20.8)
Unknown	48 (23.0)	260 (47.1)
RSV case definition^e^		
ARI or ILI	82 (39.2)	150 (27.2)
SARI	23 (11.0)	45 (8.2)
ALRI	48 (23.0)	59 (10.7)
Clinical judgment	9 (4.3)	30 (5.4)
Unknown	70 (33.5)	310 (56.2)
Testing method^f^		
Virus detection	45 (21.5)	168 (30.4)
Antigen detection	107 (51.2)	303 (54.9)
Antibody detection	8 (3.8)	42 (7.6)
Nucleic acid detection	110 (52.6)	323 (58.5)
Unknown	33 (15.8)	127 (23.0)

Abbreviations: ALRI, acute lower respiratory infection; ARI, acute respiratory infection; ILI, influenza-like illness; RSV, respiratory syncytial virus; SARI, severe acute respiratory infection.

^a^An investigation was identified as an analysis with a specified method on the reported RSV data for the seasonality of RSV activities. Analyses on the same data with different analytic methods were recognized as different investigations. A publication might include several investigations describing the seasonality of RSV activities by analyzing different sets of data or applying different analytic methods. Therefore, the sum of investigations might be greater than the total number of publications.

^b^Refers to the publications/investigations with study sites involving >1 continent.

^c^For different locations, the time for palivizumab to be licensed for prophylactic use is different. For the countries where palivizumab has not been licensed, such as China, all of the publications/investigations of this country would be recognized as “before palivizumab approved.”

^d^Refers to the publications/investigations in which the study period contains the periods both before and after the year when palivizumab was approved in the study locations.

^e^A publication/investigation on RSV seasonality might be based on multiple sources of cases; for example, ARI, ILI, and SARI perhaps were used together or in different combinations to identify patients for RSV testing. Some investigations/studies might be counted multiple times, and the sum of the proportions of publications/investigations from different case definition categories may exceed 100%.

^f^A publication/investigation on RSV seasonality might adopt multiple testing methods for RSV detection. Some investigations/studies might be counted multiple times, and the sum of the proportions of publications/investigations from different testing method categories may exceed 100%.

The information on the case source, case definition, and laboratory testing methods was missing in some investigations (case source: 260/552 [47%]; case definition: 310/552 [56%]; testing method: 127/552 [23%]). Among those with information of case source and case definition provided, the majority of investigations (152/292 [52%]) identified RSV cases from inpatients; ARI or ILI (150/242 [62%]) was the most frequently used syndromic definitions to screen patients for RSV testing, and antigen (325/552 [59%]) and nucleic acid detections (323/552 [59%]) were the most commonly used laboratory testing methods in more recent publications (Table 1, Supplementary Table 1, Supplementary Figures 2 and 3). The majority of investigations (373/552 [68%]) applied quantitative methods to determine the time of the start, peak, and/or end of seasons, and among these studies, more than half of the investigations used the threshold-based method (205/373 [55%]) (Supplementary Tables 2 and 3).

**Figure 3.**
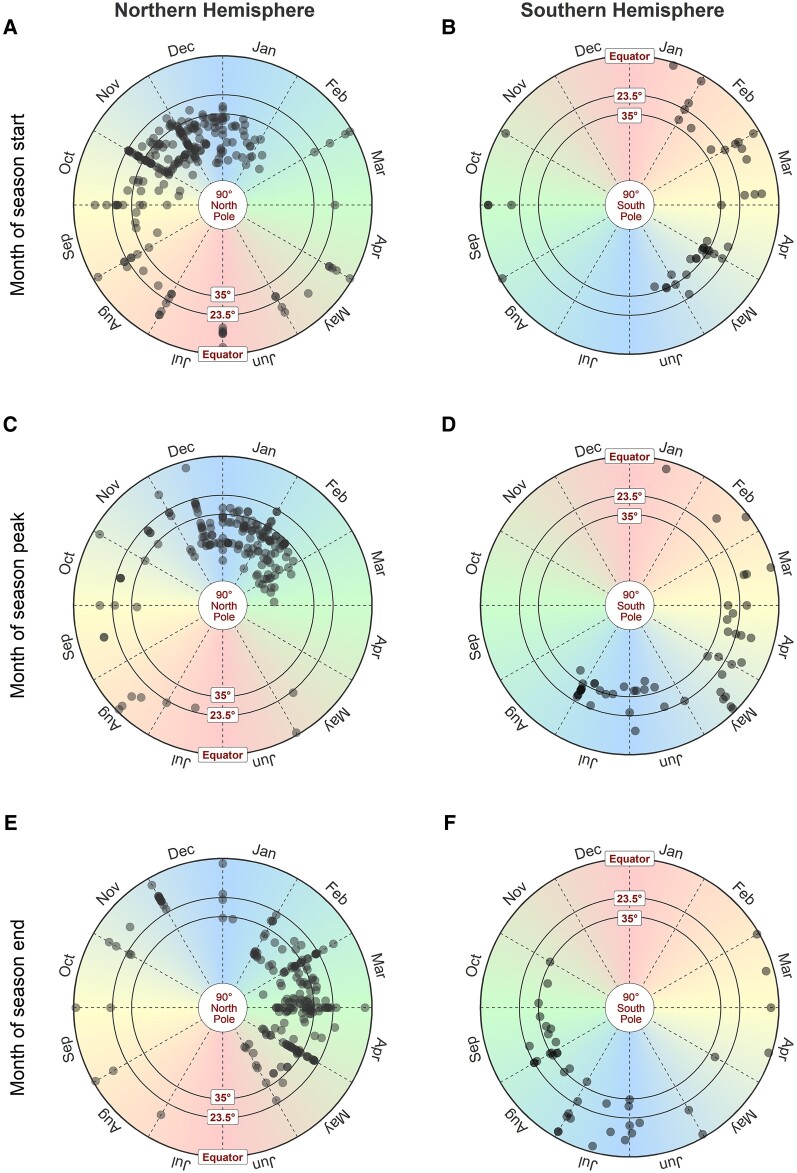
The timing of 1-peak respiratory syncytial virus (RSV) seasons reported in investigations using either quantitative or qualitative approaches against the latitude of the study site located in the Northern Hemisphere (NH, centered at the north pole) in panels *A*, *C*, and *E*, and Southern Hemisphere (SH, centered at the south pole) in panels *B*, *D*, and *F*. The start (*A*), peak (*C*), and end (*E*) of RSV seasons in NH. The start (*B*), peak (*D*), and end (*F*) of RSV seasons in SH. The center of the radius circles represents the north pole or the south pole (latitude = 90°) and the external border represents the equator (latitude = 0°). The circles were divided by the dashed radiuses into 12 sections to represent 12 months of the year. Two radius circles between the north/south pole and the external border indicating the latitudes at 35° and 23.5° are used to define the temperate region (35°–90°), subtropical region (23.5°–35°), and tropical region (equator–23.5°).

The majority of the investigations were conducted using data collected from locations in the Northern Hemisphere (NH) and indicated 1 RSV season every year (Figure 2). A small number of investigations (11/552 [2%]) reported 2 RSV peaks identified annually (Supplementary Table 4), while 18 (3%) investigations indicated unique RSV seasonal patterns alternating every 2 years (Supplementary Table 5) and 8 (1%) reported unclear RSV seasonal patterns (Supplementary Table 6). Among study sites reporting 1 RSV season annually, locations in temperate countries were fairly consistent in the time of the start, peak, and end of RSV seasons in either the NH or Southern Hemisphere (SH) (Figure 3). In temperate countries, RSV activities generally started from late autumn or early winter (ie, October–December in NH and May–June in SH), peaked in winter (December–February in NH and June–July in SH), and ended in late winter or spring (February–May in NH and July–September in SH). (Figure 3, Supplementary Figure 4). Larger variations were observed in the start, peak, and end month of RSV seasons in subtropical and tropical countries at a lower latitude, with high RSV activities observed in July–August through November in tropical locations (Supplementary Figures 5 and 6). The durations of RSV seasons were relatively shorter in study sites in the temperate region (median, 3.97 [interquartile range {IQR}, 3.00–5.00] months) compared with the estimates from subtropical (median, 4.00 [IQR, 3.52–6.14] months) and tropical countries (median, 5.00 [IQR, 4.00–6.00] months) (Figure 2, Supplementary Figure 6).

The Pearson correlation analysis indicated an overall negative association between the duration of the RSV season (in months) and the latitude both in the NH (*P* < 0.001) and the SH (*P* = 0.002) (Supplementary Table 7), and the statistical significance in the association was only indicated with the estimates derived from quantitative methods (*P* < 0.001). Positive associations were indicated in the correlation analyses between the daily average mean temperature (correlation coefficient, 0.33 [95% confidence interval {CI}, .22–.44]) (Supplementary Figures 7 and 8) and absolute humidity (correlation coefficient, 0.34 [95% CI, .22–.45]) (Supplementary Figure 9) of the study locations and the durations of RSV season estimated by quantitative methods (*P* < 0.001) but not for those estimated by qualitative methods (Supplementary Table 7). Durations of RSV seasons appeared to be longer in tropical/subtropical regions than the estimates from temperate regions, and the estimated RSV seasons were longer from the threshold-based methods but shorter from the coverage-based method (Table 2, Supplementary Table 8).

**Table 2.** Associations Identified in the Linear Regression Analysis Between Durations of Respiratory Syncytial Virus Seasons Estimated From Quantitative Approaches and Characteristics of the Studies

Variable	Change in Duration of RSV Season, Months	95% CI	*P* Value
Climatic zone (latitude)			
Temperate region (<−35° or >35°)	0	Referent	
Subtropical region (−35° to −23.5° or 23.5°–35°)	1.06	.45–1.66	<.001
Tropical region (−23.5° to 23.5°)	1.65	.77–2.54	<.001
Analysis method			
Coverage-based method	0	Referent	
Threshold-based method	1.43	.96–1.90	<.001
Model-based method	1.98	−.99 to 4.95	.19
Meteorological factor			
Daily average mean absolute humidity	0.013	−.0049 to .030	.15

Abbreviations: CI, confidence interval; RSV, respiratory syncytial virus.

In the main analysis with the linear regression model, we identified that climatic zone appeared to be independently associated with the estimated duration of RSV seasons; the analytic method used in estimating RSV seasons and the annual daily average mean absolute humidity at the study location could also explain some variations (adjusted *R*^2^ = 0.33 for estimates from quantitative approaches, 0.25 for all estimates) in the estimated durations of RSV seasons (Table 2, Supplementary Table 8). Considering the close intercorrelations between climatic zone, latitude, and temperature, we included the climatic zone (Table 2, Supplementary Table 8), latitude (Supplementary Tables 9 and 10), and daily average mean temperature (Supplementary Tables 11 and 12) of study locations into different linear regression models separately. The duration of RSV season would decrease by 0.041 (95% CI, .017–.064) months with every increase of 1 degree in latitude, and increase by 0.058 (95% CI, .0077–.11) months with every increase of 1 degree in daily average mean temperature. Durations of RSV seasons estimated for the subtropical and tropical locations were on average 1.06 (95% CI, .45–1.66) and 1.65 (95% CI, .77–2.54) months longer than for the study sites in the temperate region. Compared to the coverage-based methods, the threshold-based methods tended to produce longer estimates of the duration of RSV season by 1.43 (95% CI, .96–1.90) months. Overall, the duration of RSV season prolonged by 0.013 (95% CI, −.0048 to .030) months with every 1 g/m^3^ increase in absolute humidity at the study location (Table 2).

## DISCUSSION

RSV seasonal patterns have been studied widely in particular since the last decade, with most of the data reported from temperate countries in the NH. Our review indicated that largely similar seasonal patterns of RSV were observed in locations from the same climatic region either in the NH or the SH. In temperate regions, RSV activities primarily showed regular annual patterns starting in winter seasons, and the estimated durations of RSV season were relatively shorter than those reported from tropical and subtropical regions where prolonged RSV circulations were often more than half a year with greatly varied times of season start and/or peak.

Human infections of RSV are likely driven by 2 major antigenic groups, A and B [27], between which cross-immunity has been suggested [28, 29]. However, RSV typing is often not included in most of the established surveillance systems [30]. The increasing susceptibility in infants due to the loss of protection from waning maternal antibodies leads to a higher risk of infection, particularly in young children with high-risk conditions [1, 31, 32], potentially driving the transmission of RSV in the population [33], although the virus can infect people at any age including older adults [34, 35]. RSV shows comparable environmental survivability and transmissibility to influenza viruses with a similar estimate of the basic reproduction number (R_0_) and household second attack rate [36]. Increasing RSV activities were commonly reported in cold seasons starting from late autumn through to early spring in temperate regions, and in rainy seasons in tropical/subtropical areas, and the associations between RSV activities and environmental factors including temperature, relative/absolute humidity, and rainfall were inconsistently reported from studies conducted in tropical areas [37–39].

RSV seasonality characterized in different studies appeared to be associated with the approaches used in data collection and analysis [21]. Previous research demonstrated that the timing of RSV seasons determined with laboratory surveillance data might be different from the results based on hospitalized patient data, although the 2 estimates were highly correlated [40]. The improvement of laboratory testing methods could also affect the estimates of RSV seasons [41, 42]. Our review showed that estimates of the timing of RSV seasons were correlated with the analytic approaches used in the studies, with shorter durations of RSV seasons estimated from the “coverage-based method” (defined as the shortest time period when the majority of confirmed [severe] RSV infections occurred) than the estimates from the threshold-based methods, which were highly dependent on the often arbitrarily selected threshold of varied measures for RSV activities, such as proportion of virus detections or patients presenting ILI/ARI symptoms [43]. Therefore, prudent comparison and interpretation of RSV seasons estimated from different studies or with different approaches is necessary.

RSV surveillance is often established based on the existing routine surveillance on influenza, more commonly seen in developed economies including the US [44], Canada [45], and some European countries [46]. The World Health Organization initiated a pilot RSV surveillance scheme by leveraging the current Global Influenza Surveillance and Response System covering >100 countries to explore possible surveillance strategies including case definitions, laboratory methods, and target population, etc [47]. Current RSV surveillance largely applied similar syndromic case definitions as those used for influenza surveillance (ie, sampling patients showing ILI and testing for the virus), whereas the validity of using such case definitions for RSV surveillance has been questioned [48]. In some countries/areas RSV surveillance was not conducted throughout a year but only during restricted period(s) following the influenza surveillance protocol given the regular annual influenza seasons in those locations [19, 49]. However, this might lead to an underreporting of RSV infections during the nonsurveillance periods [50].

During the COVID-19 pandemic, circulation of respiratory viruses including RSV changed considerably worldwide largely due to implementation of nonpharmaceutical interventions and changes in human behaviors in response to the pandemic [51, 52]. RSV remained at a low level in many countries in 2020, when public health and social measures were widely adopted globally to contain the spread of severe acute respiratory syndrome coronavirus 2, but caused surges of infections with a large number of severe cases admitted into hospital in some countries when some interventions were relaxed from 2021 onward [53, 54], including the US during 2021–2023 [55], some geographically separated locations in Australia in 2020–2021 [56], and the United Kingdom in 2021–2022 [57]. The interseasonal viral activities were perhaps related to the increased susceptibility in the population due to the lack of exposure to RSV during the previous years in response to COVID-19 [58].

Monoclonal antibodies approved for prophylactic therapy provide immediate protection of high-risk individuals, particularly young children, against RSV-associated severe infections [7, 13]. Five monthly injections of palivizumab and a single dose of nirsevimab can substantially reduce the risk of hospitalizations due to RSV in individuals receiving the therapy throughout RSV seasons, especially in temperate regions where RSV seasons would normally not be longer than 5 months. The duration of protection provided by RSV vaccines has not been fully determined, although promising efficacy for short-term prevention from infections was demonstrated in clinical trials [14, 16, 59]. The effectiveness of the abovementioned pharmaceutical interventions for high-risk populations is determined by the duration of protection of the monoclonal antibodies and vaccines, the timing of administration, and RSV activities in specific locations. Ideally these preventive measures should be administered before an RSV season starts and the protection can be maintained throughout the season, likely during November–April and May–October in temperate regions of the NH and the SH, respectively. However, great challenges in implementing these efficacious interventions would be seen in locations where there were irregular seasons, particularly with prolonged viral circulations [60–62]. Based on the reported seasonal patterns in locations from northern temperate regions in this review, studies incorporating epidemiologic analysis on RSV seasonality into the effectiveness and cost-effectiveness of these RSV preventive strategies are urgently needed [63].

There are several limitations in this study. First, data of RSV seasonality that were published and included in the review were skewed to high-income countries with well-established RSV surveillance systems. These countries are largely in temperate regions where RSV circulation might be different from the underrepresented middle- and low-income countries due to varied population structure, contact patterns, and geoclimatic features [22]. Second, RSV antigenic information could not be incorporated into the current analysis of seasonality due to the lack of data. Expanding RSV surveillance by collecting antigenic and genetic data is urgently needed to improve our understanding of the transmission dynamics of RSV locally and globally [64]. Third, except for the analytic method, we could not examine potential associations between RSV seasonality and other study characteristics such as case sources, case definitions, and laboratory methods due to the lack of information and nonstandardized laboratory protocols used for RSV surveillance; we also could not explore the association between RSV seasonality and population structure, particularly birth rate, given the lack of data, although the high disease burden of RSV identified in young children [65–67] implies a possible driving force from the young population on RSV activities. Last, the reported data of seasonality in this review did not allow us to investigate the RSV-associated disease burden that occurred during the characterized RSV seasons, which is a critical metric for evaluation of the health impact related to RSV circulation, particularly when there were large uncertainties in the start and the end of RSV seasons in a specific location and the infection risk is less likely to be homogeneous in a population over the season.

## CONCLUSIONS

This study provided a global overview of RSV seasonality before the COVID-19 pandemic. The seasonal patterns illustrated in this review may inform optimal use of preventive and therapeutic interventions against RSV infection, particularly in temperate regions of the NH and SH. Considerable variations in methods used by different studies highlighted the importance of developing and applying standardized approaches in RSV surveillance and data reporting. Further research is needed to understand the underlying mechanisms driving RSV seasonal patterns locally and globally.

## Supplementary Material

ofae238_Supplementary_Data

## Supplementary Data

Supplementary materials are available at *Open Forum Infectious Diseases* online. Consisting of data provided by the authors to benefit the reader, the posted materials are not copyedited and are the sole responsibility of the authors, so questions or comments should be addressed to the corresponding author.

## Notes

***Acknowledgments.*** The authors thank Prof. Julian W. Tang and Dr Eeva Broberg for kindly sharing the data of RSV seasonality from their publications. The authors also thank Stella S. W. Lai for technical assistance.

***Author contributions.*** S. S., P. W., Y. B., and Z. D. conceived and designed the study. S. S., W. Z., H. G., and P. H. collected and extracted data. S. S. and P. W. conducted the data analysis and wrote the first draft of the manuscript. All authors contributed to the interpretation of the results and revision of the manuscript, and have approved the final version of the manuscript.

***Patient consent.*** Data used in this review were collected from published studies, and patient consent is not applicable for this review.

***Disclaimer.*** The funding bodies had no role in study design, data collection and analysis, preparation of the manuscript, or the decision to publish.

***Financial support*.** This project was supported by the Health and Medical Research Fund (project number 18171252) from the **Food and** Health Bureau of Hong Kong SAR Government, and AIR@InnoHK administered by the Innovation and Technology Commission - Hong Kong.
