# Supplementary material for: Global Seasonal Activities of Respiratory Syncytial Virus Before the Coronavirus Disease 2019 Pandemic: A Systematic Review
Source: Open Forum Infect Dis. 2024 Apr 25;11(5):ofae238. doi: 10.1093/ofid/ofae238 (PMC11103620; doi:10.1093/ofid/ofae238)
Supplement: ofae238_Supplementary_Data [file ofae238_supplementary_data.docx]

## **SUPPLEMENTARY INFORMATION**

**Global seasonal activities of respiratory syncytial virus before the COVID-19 pandemic: a systematic review**

**Literature search and selection**

We performed a literature search on PubMed on 17 January 2023 with search terms pertaining to respiratory syncytial virus (RSV) (#1 “RSV OR respiratory syncytial virus”), and seasonlity (#2 “seasonality OR season*”) in the “Title/Abstract” field, without restrictions on the publication time and language. The search results were shown below.

|  | **Search terms** | **Number of publications identified** |
| --- | --- | --- |
| RSV disease | #1: (RSV OR respiratory syncytial virus) | 23,005 |
| Seasonality | #2: (Seasonality OR season*) | 201,170 |
| RSV seasonality | #3: #1 AND #2. | 2,510 |

After removing the duplicates and articles not meeting the inclusion criteria (See Methods), our review focused on publications estimating RSV seasons with a study period of three years or longer. If a publication included data from a time period beyond the year 2020, only the data before January 1, 2020 would be included into the analysis. Besides, the remaining study period after excluding the data from 2020 onwards should be at least three years. For example, if a publication applied the data collected from 2016 through to 2021 to estimate RSV seasonality, we would only include RSV seasonal patterns indicated by the data collected during 2016-2019 into our analysis.

**Latitude coordinates of the studied sites**

We extracted the latitude and longitude coordinates for each study site with RSV seasonal patterns reported using Google Geocoding API [[1]](https://paperpile.com/c/zPIKrB/pdyI). For multicenter studies we used the coordinates of the centroid of multiple centers. For the 10 Health and Human Services (HHS) [213] regions of the United States, we used coordinates of the corresponding reference cities following the study by [Staadegaard](http://paperpile.com/b/zPIKrB/aIPfL) et al. [[2]](https://paperpile.com/c/zPIKrB/aIPfL).

**Temperature and absolute humidity of studied sites**

We used the latitudes inferred by the above-mentioned method to represent the study sites and collected the daily average temperature, average relative humidity from the nearest weather station providing the data within the study period using R package GSODR. The daily average absolute humidity was calculated from the temperature and relative humidity. Each season estimate was associated with a value of dialy average mean temperature and daily average mean absolute humidity by calculating the mean of the daily average temperature and daily average absolute humidity within the study period for further analysis.

**Categorization of RSV seasonal patterns**

We classified the RSV seasonal patterns into four different types based on the reported season timing (the start, peak and end) during the study period.

1. One RSV season a year. RSV activity presented one season every year, and the time of RSV season start, peak and end were similar during the study period (differences of season starts, peaks, and ends across different years were within 3 months). There was only one peak of RSV activity reported in a year during the study period.
2. Two-year cycle. The seasonality of RSV presented a two-year cycle in terms of the timing of season start or peak, or the magnitude of peak in the study period, i.e. RSV activity patterns repeatedly occur every 2 years. Under this pattern, RSV activity may exhibit an alternating pattern with an early season followed by a late season, and/or a mild outbreak followed by a markedly intense one.
3. Two RSV peaks a year. Two peaks of RSV activities occurred in one year during the study period.
4. Unclear pattern. If the original study reported that RSV seasons were not able to be clearly identified, or the timing of RSV seasons (the start, peak, and end) changed substantially across years (differences were larger than 3 months), we put it into the category of “unclear pattern”.

## **Categorization of analysis methods**

We coarsely categorized the methods to determine the RSV seasons as qualitative methods and quantitative methods. If the analysis of RSV seasonality in an investigation was carried out using a statistical or mathematical approach, it would be classified into quantitative methods. Otherwise, if the seasonality of RSV was only reported by descriptive texts and/or graphic illustrations, it would be of qualitative methods.

We further classified the quantitative methods into three types based on the approaches used to determine the RSV seasons: 1) threshold-based methods, which means that if the indicator of RSV activity is beyond a predefined threshold RSV season begins. The indicators used in the methods were different, and the mostly used indicators were positive percentage (what percentage of samples were tested positive), the number of RSV cases, and RSV hospitalization. The thresholds could be defined as fixed values, or dynamically calculated from historical data. Possibly there were additional requirements for the season start other than beyond the threshold values, such as meeting threshold values for a minimum number of consecutive weeks/months, or the minimum requirement of testing/positive samples ([Supplementary Table](#sta_7) 6). 2) coverage-based methods, which basically defines RSV seasons as a probably shortest consecutive temporal period or intermittent periods covering at least a certain percentage of RSV cases (for example, 75% in [[3]](https://paperpile.com/c/zPIKrB/BQ74y)). 3) model-based methods, which apply certain statistical models (e.g. Poisson regression model [[4]](https://paperpile.com/c/zPIKrB/JH4bW), wavelet model [[5]](https://paperpile.com/c/zPIKrB/o6J01) etc.) to the RSV surveillance data and the seasons were determined based on the fitting results.

**Categorization of case definitions**

Case definition refers to the syndromic definition of the patients selected for RSV testing. We categorized the case definition into four groups, ARI or ILI, SARI, ALRI and clinical judgment. For the studies that the case definition was not clearly described, we categorized them as “Unknown”. If the case definition was described as either ARI (acute respiratory infection) or ILI (influenza-like illness), or as “respiratory disease”, “respiratory symptoms”, the case definition of this study would be classified as “ARI or ILI”. The type “SARI” included the studies in which the criteria were described as “SARI” or hospitalized for respiratory illness. “ALRI” included the inclusion criteria of lower respiratory tract infection including pneumonia and bronchitis. If the inclusion criteria was described as clinical requirement or clinician's discretion, the study would be categorized as “clinical judgment”.

**Categorization of testing methods**

We categorized the testing methods adopted in the included studies into four categories by the substance they detect to confirm RSV infection: virus detection, antibody detection, antigen detection, and nucleic acid detection. Testing methods like cell culture and virus isolation were classified into the group of virus detection, serology and HAI were classified as antibody detection, IF, DFA, EFA, ELISA were generally classified as antigen detection if they test the existence of antigen, and PCR and molecular method were categorized as nucleic acid detection.

**The timing of RSV seasons**

The timing of RSV season (the start, peak, and end) were extracted from the included studies. For the studies using qualitative methods, the estiamtes about the season start, peak and end were extracted from the descriptive text of RSV seasons. For example, from description of “ RSV seasons occur during the winter months from November to March”, season start as November and season end as March were extracted.

All the extracted estimates of the season start, end and peak were transformed to numerical values between 0 to 12, 0-1 representing January and 1-2 representing February, and so on. We assume that there is 52 weeks in a year (no week 53), and weeks 1-52 were evenly transformed to the scope of 0-12.

The timing of RSV seasons (the start, peak, and end) were reported in different formats. The estimates might be a specific month or week, such as “November” or “Week 42”, as a range like “from November to December”, or both with a specific time as the median or mean of multi-year timing and a period representing the range, 95% confidence interval or interquartile range such as “Week 42 (41-45)”.

In Supplementary Figure 4, we tried to provide as much information in the studies as possible, so we plotted the period (range, 95% CI, or IQR) of RSV season start, end and peak when they were available, and plotted the specific week/month otherwise. While in the correlation and regression analysis, the duration of RSV season was calculated using the specific estimate when available, or using the mid-time point of a reported duration when the estimate of a specific time point was not available.

**Linear regression model of durations of RSV seasons with the latitude, climatic zone, absolute humidity and analytic method**

The linear regression model below was used to investigate the relationship between duration of RSV seasons and the latitude, climatic zone, absolute humidity and analytic method. The model was separately applied to quantitative estimates (Table 2) and all estimates (Supplementary Table 8) of RSV seasons for a period not less than 3 years from the included studies.

$$y_{i}=\beta_{0}+\beta_{1}x_{i1}+\beta_{2}x_{i2}+\beta_{3}x_{i3}$$

Where

$i=n$ observed RSV duations

$y_{i}=RSV duration in months$

$$\beta_{0}=intercept (constant term)$$

$$x_{i1}=climatic zone$$

$$x_{i2}=daily average mean absolute humidity$$

$$x_{i3}=analytic method$$

$\beta_{1},\beta_{2}, \beta_{3}$are the corresponding coefficients for $x_{i1},x_{i2},$ and $x_{i3}$

We also applied alternate regression models using the absolute value of latitude and daily average mean temperature to relplace the climatic zone (let $x_{i1}$be the daily average mean temperature and absolute value of latitude in the above regression equation, respectively) to investigate the association between study characteristics and estimated durations of RSV seasons from quantitative methods (Supplementary Table 9 and Supplementary Table 11) and all estimates (Supplementary Table 10 and Supplementary Table 12).

**Possible strategies in prophylactic use of monoclonal antibodies**

Based on the RSV seasonality identified in this review, we intended to examine possible strategies in applying currently available preventive pharmaceutical interventions against RSV epidemics. As indicated in previous efficacy studies and recommendations from the professional organizations, the two approved monoclonal antibodies, palivizumab and nirsevimab, presumably could provide 5-month protection (one dose of nirsevimab or 5 doses of palivizumab) for the individual treated.

Substantial uncertainties of RSV seasonal patterns resulted from the fact that the start or the end the RSV season was reported as a time period covering multiple months, which surely would bring challenges in determining the optimal time for the use of prophylactic therapies in the target population. We investigated the coverage of durations of RSV seasons of 123 distinct estimates extracted from 76 different locations in northern temperate regions where less heterogeneous seasonal patterns were indicated if administering the monoclonal antibodies to provide 5-month protection. If using the month of the mid-point of the reported season start period as the possible time for initiation of the prophylactic therapy for the target population, and the month of the mid-point of the reported season end period as the end of the season, the seasons of the locations in temperate countries were shown to start from autumn to winter months (median: December, range: September-February), and end in winter to spring (median: March, range: December-May), leading to the durations of the RSV seasons being 2 to 8 months (median: 4 months). Assuming that all the 123 locations in northern temperate regions start to administer the monoclonal antibody for the target population in November, 355/563 (60·1%) RSV season months would be covered in these locations, administration in December leading to treated individuals being protected in 480/563 (85·3%) RSV season months, and the initiation in January covering 406/563 (72·1%) season months. If the prophylactic therapy can be initiated exactly on the reported month of season start in each location, the targe population can only be protected in 521/563 (92.5%) RSV season months. However, we were aware that data of seasonality from some locations might be over represented in this analysis.

Alternatively, if using the earliest month of the reported season start period as the possible time for initiation of the prophylactic therapy, and the latest month of the reported season end period as the end of a season, the median months of season start and end were November (range: July-February) and April (range: December – June), respectively, with the median duration of RSV seasons being 5 months (range: 2-9). Assuming that all the 123 locations in northern temperate regions initiated the therapy from November, it would cover 350/563 (62·2%) season months, 516/633 (81·5%) season months if starting from December, 445/633 (70·3%) months if from January. Even if the prophylactic therapy can be initiated exactly on the reported month of season start in each location, the targe population can only be protected in 553/633 (87·4%) RSV season months.

**SUPPLEMENTARY FIGURES**

**
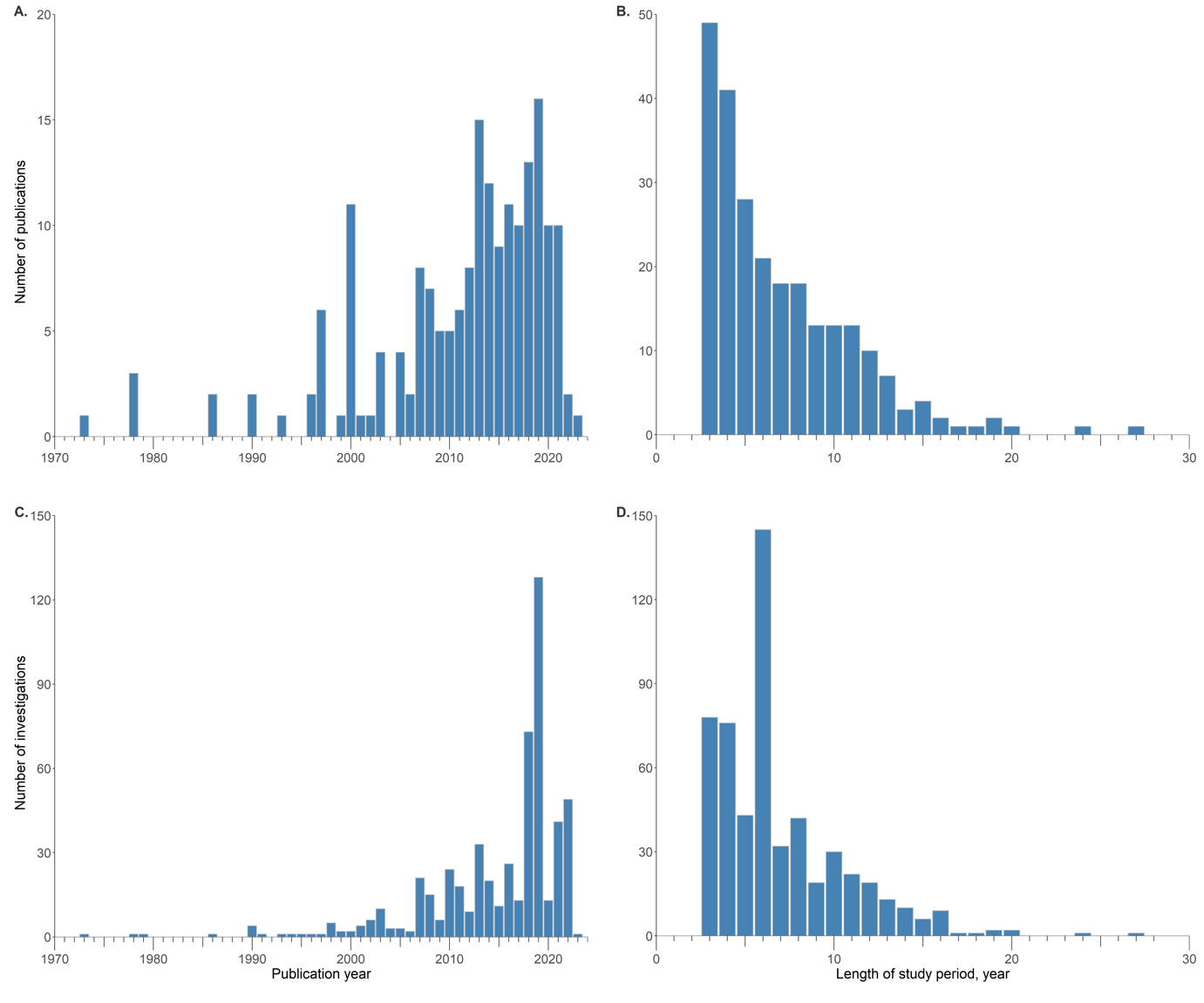
**

**Supplementary Figure 1** Annual numbers of publications and the investigations on RSV seasonality as of January 17, 2023 and the distribution of length of study period. (A) Annual number of included publications. (B) The distribution of length of study period for the included publications. (C) Annual number of investigations in the selected publications. (D) The distribution of length of study periods for the investigations in the selected publications.

**
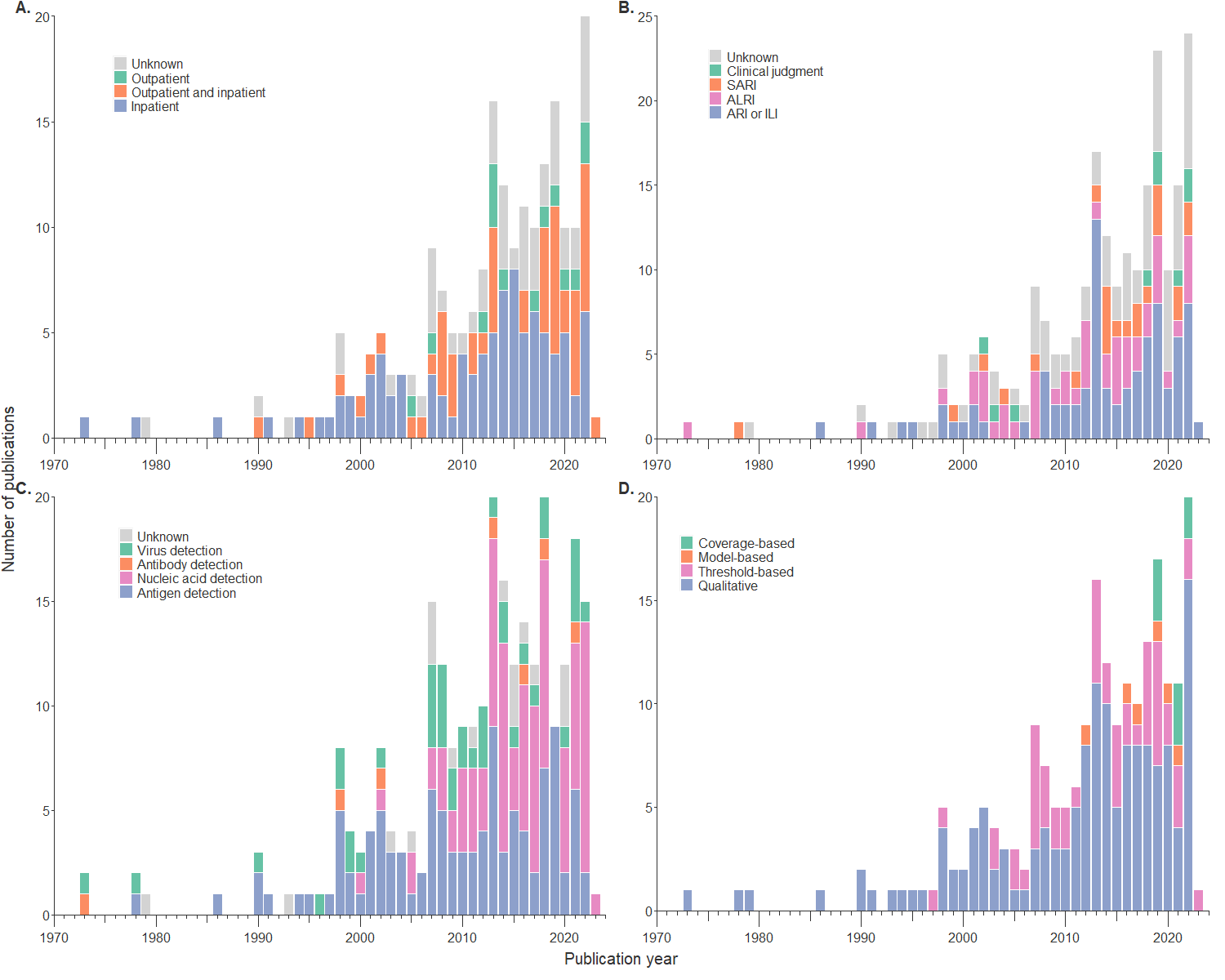
**

**Supplementary Figure 2.** The distribution of case sources (A), case definitions (B), testing methods (C) and analytic methods (D) adopted in the included publications by publication year.

**
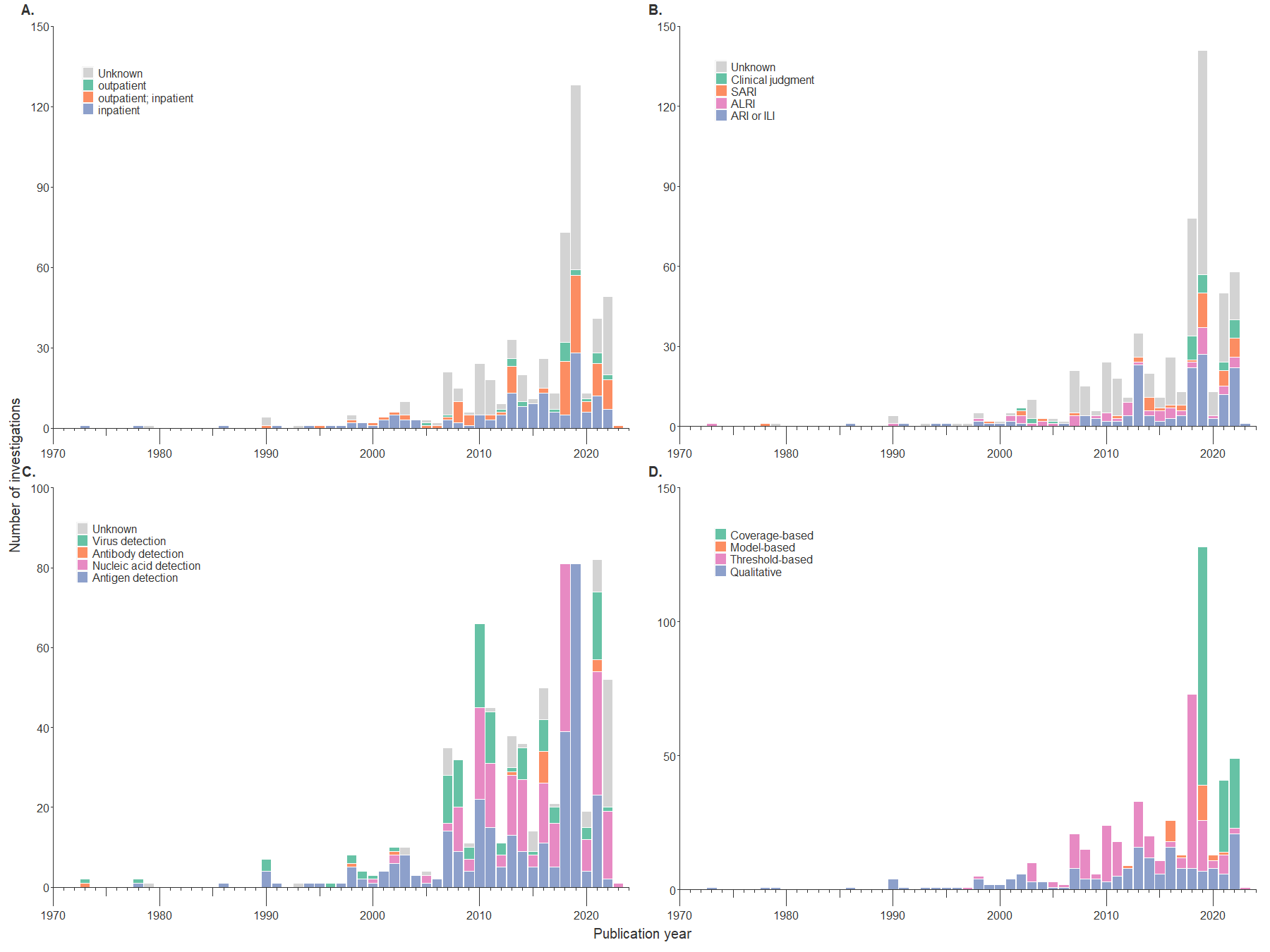
**

**Supplementary Figure 3.** The distribution of case sources, case definitions, testing methods and analysis methods adopted by the investigations in the selected publications by the publication year. (A) The distribution of case sources. (B) The distribution of case definitions.(C) The distribution of testing methods. (D) The distribution of analysis methods.

**
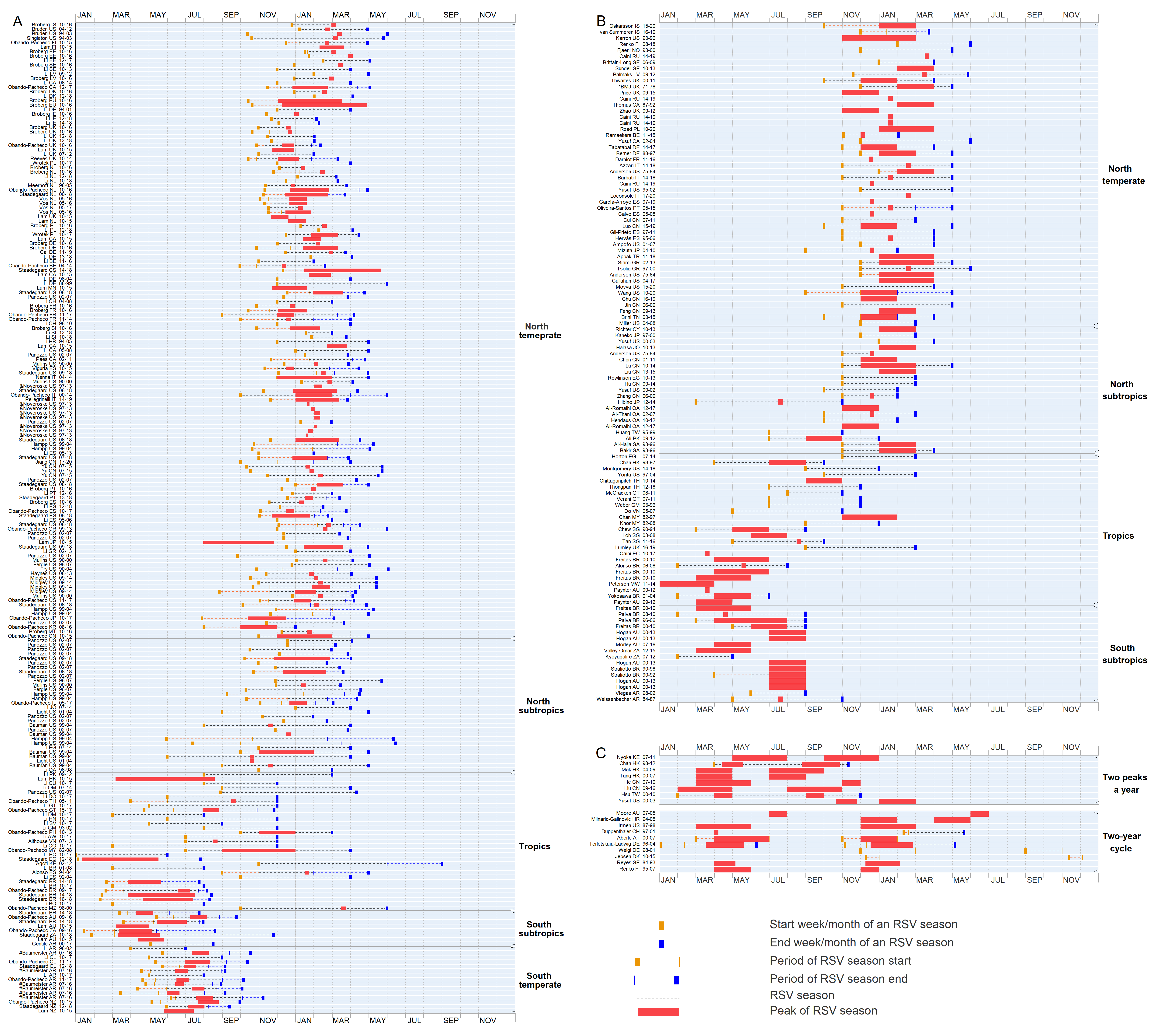
**

***** The title of this publication was not available, so we used the publisher instead.

& The 95% confidence interval of estimates of season timing were plotted.

# The IQR (interquatile range) of estimates of RSV season timing were plotted.

$ The estimate of RSV season was associated with multiple countries, only the first country was shown.

**Supplementary Figure 4.** The reported time of the start, peak and/or end of RSV seasons from the investigations with the study period for 3 years or longer. Black lines denote the duration of the RSV season, red bars represent the peak period of reported RSV seasons. Orange segments indicate the reported week/month of the RSV season start/end, with the extended dashed line indicating the season start (to the right) or end (to the left) reported as a time period. The studies were ordered according to the centre latitude of the study site from the north (top) to the south (bottom). Estimated timing of RSV seasons for investigations classified as “One RSV peak a year” by quantitative methods (A) or by qualitative methods (B), and investigations classified as “Two-year cycle” and “Two RSV peaks a year” by either quantitative or qualitative methods (C).

**
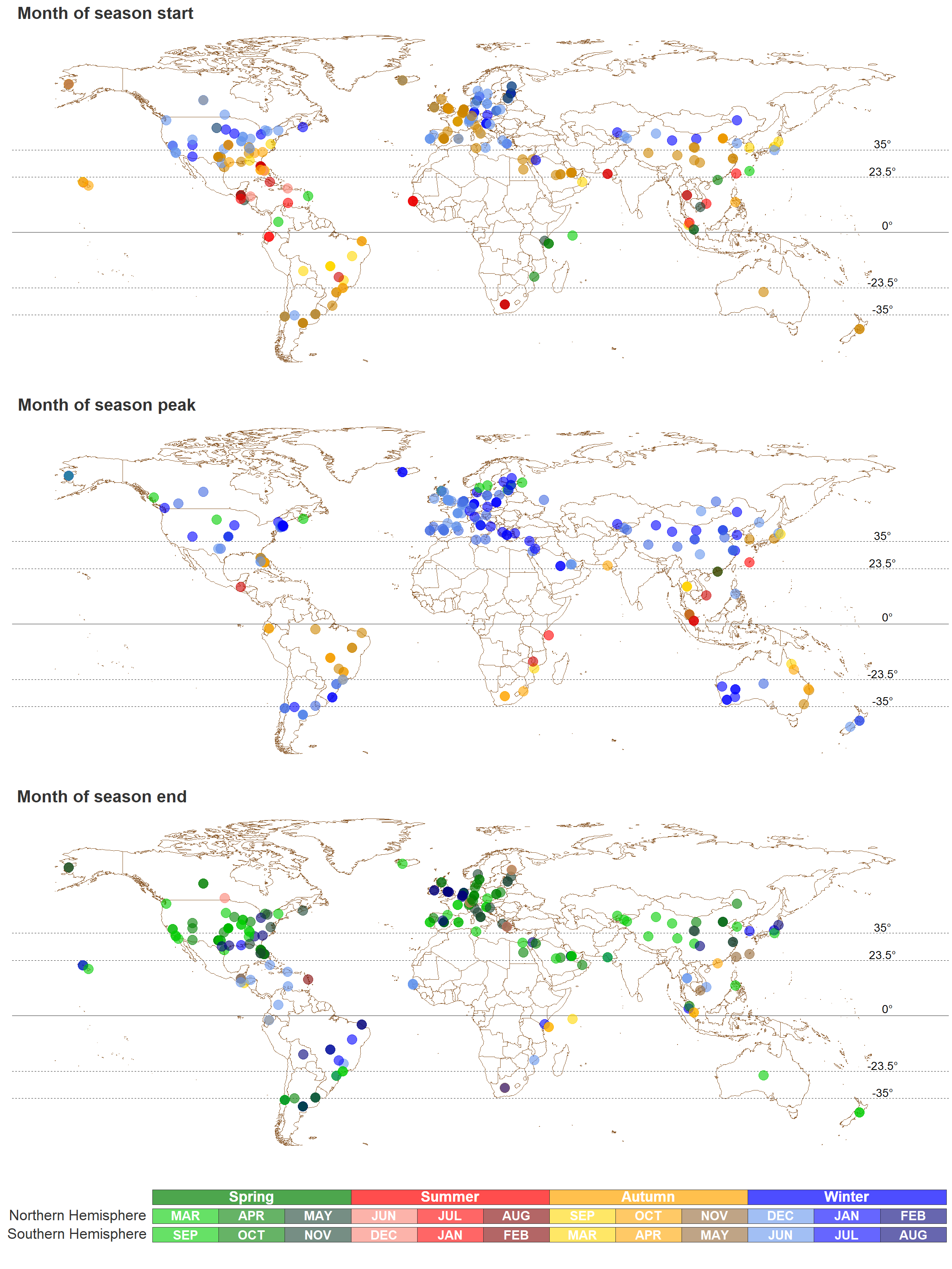
Supplementary Figure 5.** Global map of the estimates on the start, peak, and end of RSV season from the included publications. (A) The start of RSV seasons. (B) The peak of RSV seasons. (C) The end of RSV seasons.

**
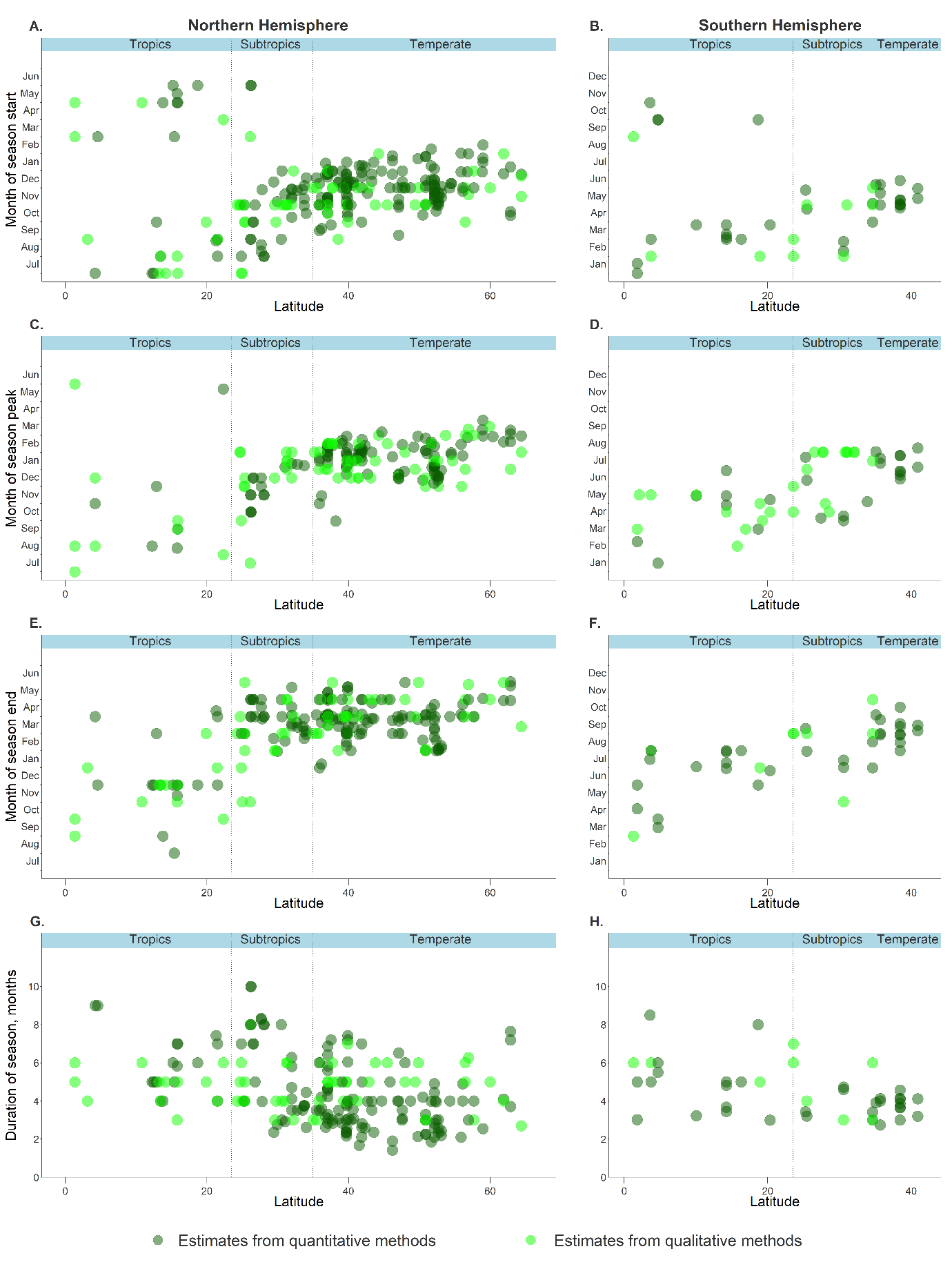
Supplementary Figure 6.** The timing (the start, peak, and end) and duration of RSV seasons extracted from the included publications with the latitudes of the study sites. The start of RSV seasons with the latitudes in the Northern Hemisphere (A) and Southern Hemisphere (B). The peak of RSV seasons with the latitudes in the Northern Hemisphere (C) and Southern Hemisphere (D). The end of RSV seasons against the latitudes in the Northern Hemisphere (E) and Southern Hemisphere (F). The duration of RSV seasons against the latitudes in the Northern Hemisphere (G) and Southern Hemisphere (H). The dark green points represent the estimates using quantitative methods, and the light green points are for the estimates from qualitative approaches.

**
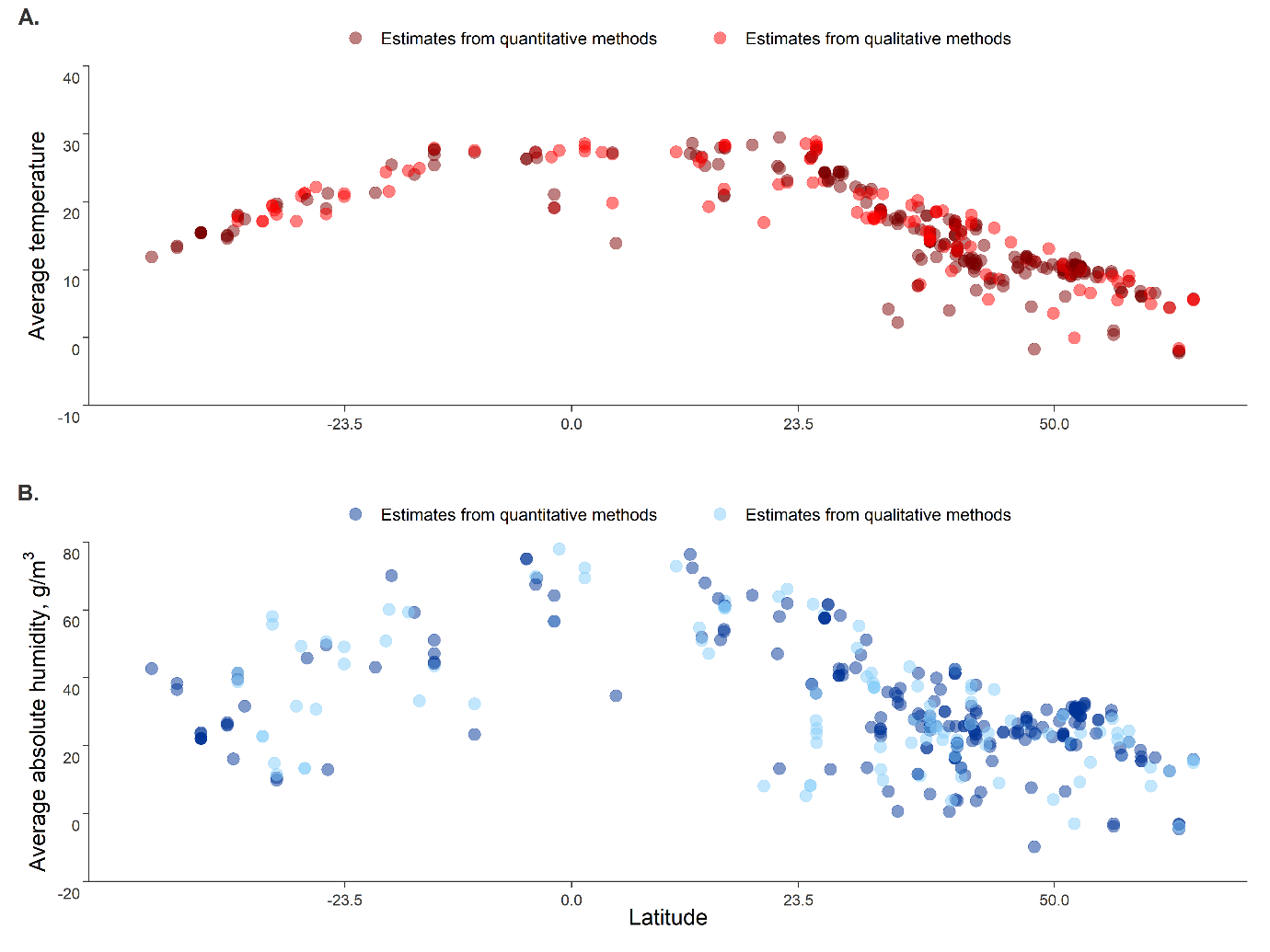
Supplementary Figure 7.** The daily average mean temperature and daily average mean absolute humidity of the study sites during the study period by the latitudes of the study sites.

**
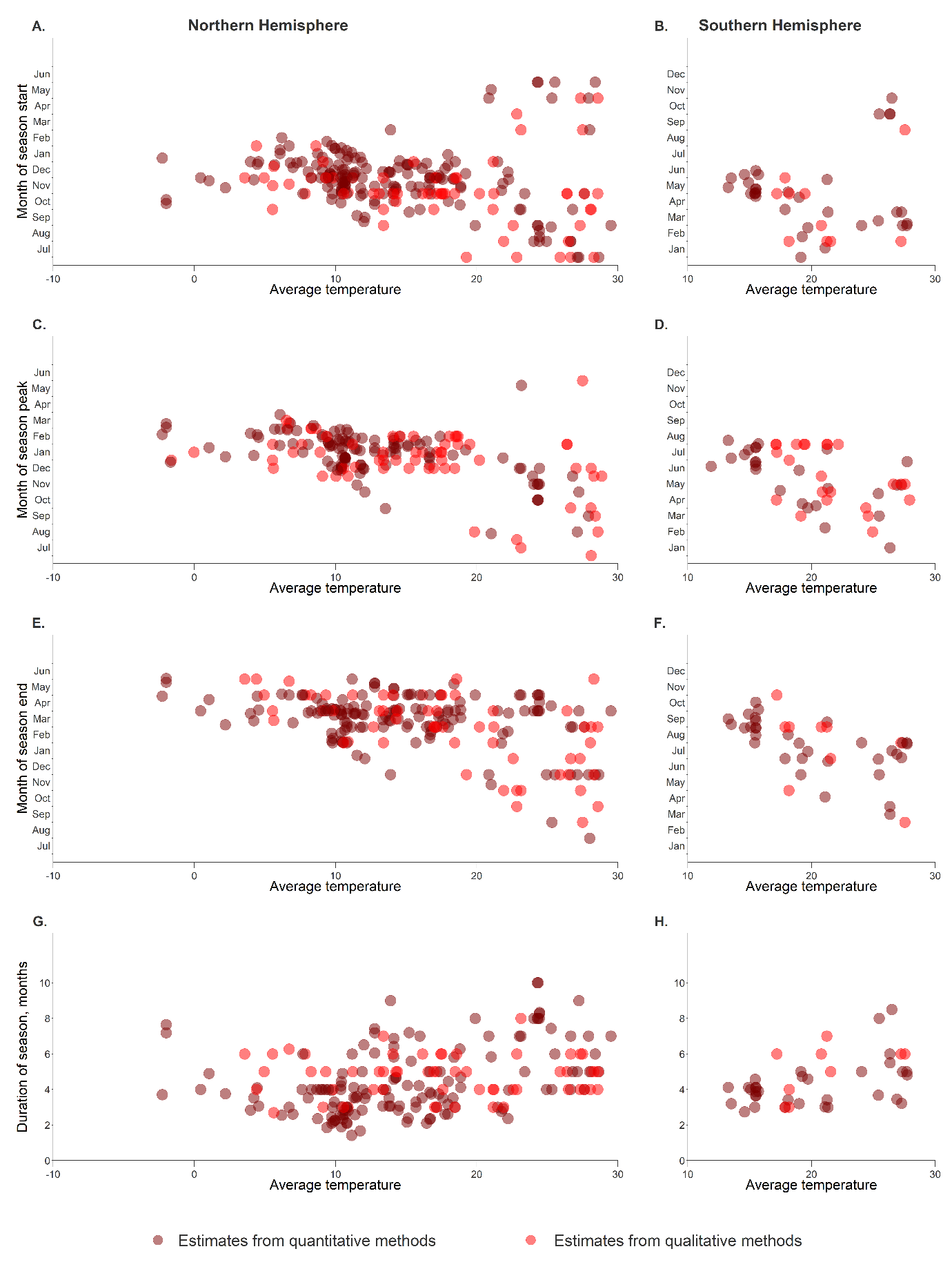
**

**Supplementary Figure 8.** The timing (the start, peak, and end) and duration of RSV seasons extracted from the included publications agaist the daily average mean temperature of the study sites during the study period. The start month of RSV seasons agaist the daily average mean temperature in the Northern Hemisphere (A) and Southern Hemisphere (B). The peak month of RSV seasons agaist the daily average mean temperature in the Northern Hemisphere (C) and Southern Hemisphere (D). The end month of RSV seasons agaist the daily average mean temperature in the Northern Hemisphere (E) and Southern Hemisphere (F). The duration of RSV seasons in months agaist the daily average mean temperature in the Northern Hemisphere (G) and Southern Hemisphere (H). The dark red points represent the estimates using quantitative methods, and the light red points are for the estimates from qualitative approaches.

**
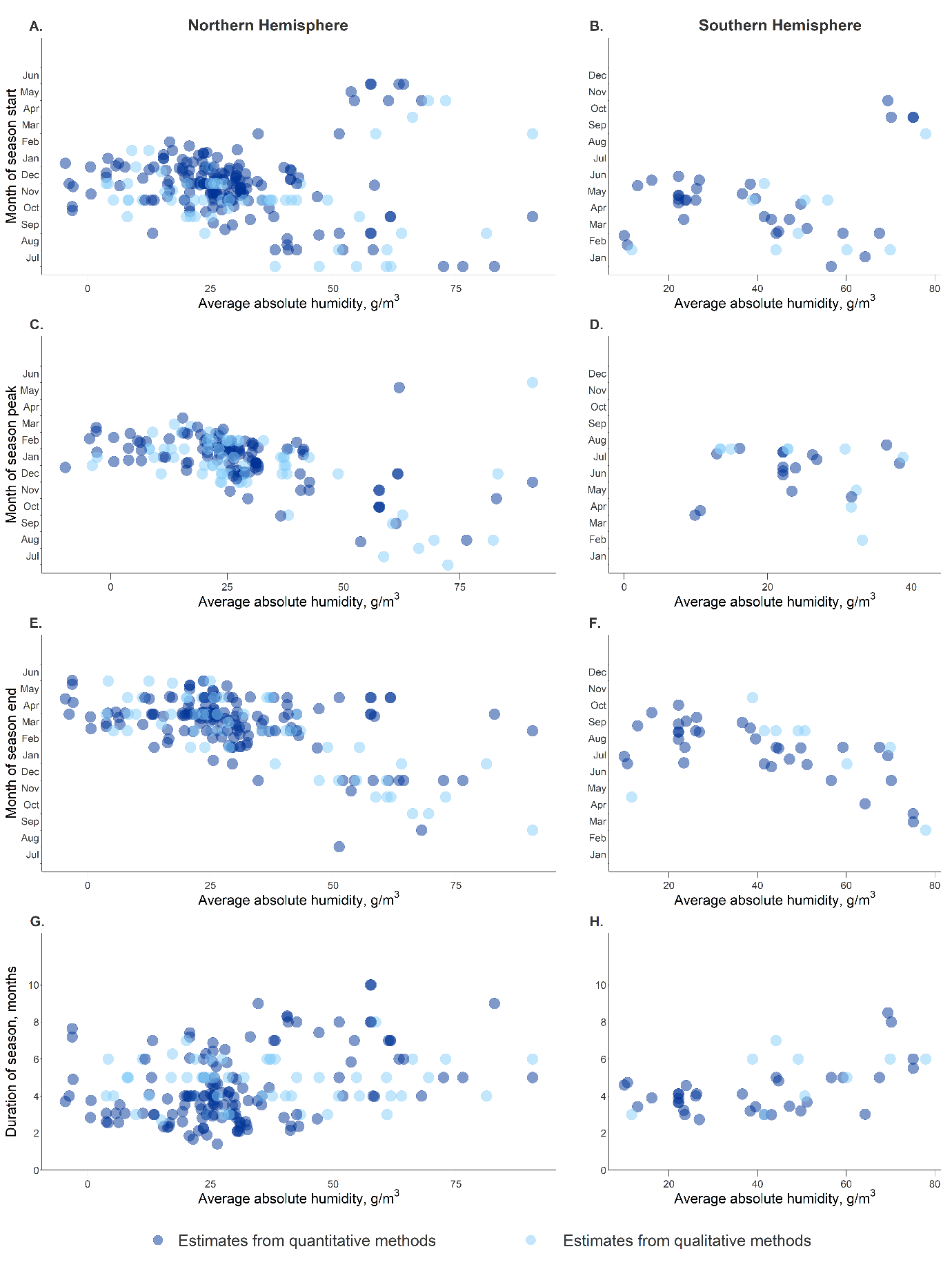
Supplementary Figure 9.** The timing (the start, peak, and end) and duration of RSV seasons extracted from the included publications against the daily average mean absolute humidity of the study sites during the study period. The start of RSV seasons agaist the average absolute humidity in the Northern Hemisphere (A) and Southern Hemisphere (B). The peak of RSV seasons agaist the daily average mean absolute humidity in the Northern Hemisphere (C) and Southern Hemisphere (D). The end of RSV seasons agaist the daily average mean absolute humidity in the Northern Hemisphere (E). and Southern Hemisphere (F). The duration of RSV seasons against the daily average mean absolute humidity in the Northern Hemisphere (G) and Southern Hemisphere (H). The dark blue points represent the estimates using quantitative methods , and the light blue points are for the estimates from qualitative approaches.

**SUPPLEMENTARY TABLES**

**Supplementary Table 1**. Description of the sites and study characteristics of the associated investigations included in the systematic review.

| **Publication** | **Country** | **Location** | **Longitude and latitude** | **Study period** | **Case source** | **Case definition** | **Testing method** | **Season method** | **Season scale** |
| --- | --- | --- | --- | --- | --- | --- | --- | --- | --- |
| Aberle, 2008 [[6]](https://paperpile.com/c/zPIKrB/xZQ74) | Austria | Vienna | 16·4, 48·2 | 2000 - 2007 | inpatient | ARI or ILI | Nucleic acid detection | Qualitative | Month |
| Agoti, 2015 [[7]](https://paperpile.com/c/zPIKrB/HZLo) | Kenya | Kilifi | 39·9, -3·6 | 2002 - 2012 | inpatient | ALRI | Antigen detection; Nucleic acid detection | Threshold-based | Month |
| AI-Assam, 2009 [[8]](https://paperpile.com/c/zPIKrB/tfn1D) | Canada | nationwide | -106·3, 56·1 | 2005 - 2008 | outpatient; inpatient | ARI or ILI | Virus detection; Antigen detection; Nucleic acid detection | Qualitative | Month |
| AI-Assam, 2009 [[8]](https://paperpile.com/c/zPIKrB/tfn1D) | Canada | Nova Scotia | -63·7, 44·7 | 2005 - 2008 | outpatient; inpatient | ARI or ILI | Virus detection; Antigen detection; Nucleic acid detection | Qualitative | Month |
| AI-Romaihi, 2019 [[9]](https://paperpile.com/c/zPIKrB/rqUb) | Qatar | Doha | 51·5, 25·3 | 2012 - 2017 | outpatient | ARI or ILI | Nucleic acid detection | Qualitative | Month |
| AI-Romaihi, 2020 [[10]](https://paperpile.com/c/zPIKrB/ohzb) | Qatar | nationwide | 51·2, 25·4 | 2012 - 2017 | outpatient | ARI or ILI | Nucleic acid detection | Qualitative | Month |
| AI-Thani, 2008 [[11]](https://paperpile.com/c/zPIKrB/x39d) | Qatar | nationwide | 51·2, 25·4 | 2002 - 2007 | outpatient; inpatient | ARI or ILI | Antigen detection | Qualitative | Month |
| Al-Hajja, 1998 [[12]](https://paperpile.com/c/zPIKrB/LxVi) | Saudi Arabia | Riyadh | 46·7, 24·7 | 1993 - 1996 | Unknown | ARI or ILI | Antigen detection | Qualitative | Month |
| Ali, 2017 [[13]](https://paperpile.com/c/zPIKrB/QU8n) | Pakistan | Karachi | 67, 24·9 | 2009 - 2012 | inpatient | ARI or ILI | Nucleic acid detection | Qualitative | Month |
| Alonso, 2007 [[14]](https://paperpile.com/c/zPIKrB/9fNn0) | Spain | Sacyl | 41·7, -4·7 | 1992 - 2004 | inpatient | ALRI | Antigen detection | Threshold-based | Month |
| Alonso, 2012 [[15]](https://paperpile.com/c/zPIKrB/YD3c) | Brazil | Fortaleza | -38·5, -3·7 | 2006 - 2008 | Unknown | ARI or ILI | Antigen detection | Qualitative | Month |
| Althouse, 2018 [[16]](https://paperpile.com/c/zPIKrB/tl6A9) | Vietnam | Nha Trang | 109·2, 12·3 | 2007 - 2012 | inpatient | ARI or ILI | Nucleic acid detection | Threshold-based | Month |
| Ambrose, 2019 [[17]](https://paperpile.com/c/zPIKrB/gWGAj) | USA | nationwide | -95·7, 37·1 | 2011 - 2016 | Unknown | Unknown | Virus detection; Antigen detection; Nucleic acid detection | Threshold-based | Week |
| Ampofo, 2008 [[18]](https://paperpile.com/c/zPIKrB/jlUM) | USA | Utah | -111·1, 39·3 | 2001 - 2007 | outpatient; inpatient | ARI or ILI | Virus detection; Antigen detection | Qualitative | Month |
| Anderson, 1990 [[19]](https://paperpile.com/c/zPIKrB/eub9) | USA | Colorado, Pennsylvania, and Washington | -100·7, 44·3 | 1975 - 1984 | Unknown | Unknown | Virus detection; Antigen detection | Qualitative | Month |
| Anderson, 1990 [[19]](https://paperpile.com/c/zPIKrB/eub9) | USA | nationwide | -95·7, 37·1 | 1975 - 1984 | Unknown | Unknown | Virus detection; Antigen detection | Qualitative | Month |
| Anderson, 1990 [[19]](https://paperpile.com/c/zPIKrB/eub9) | USA | Texas state | -99·9, 32 | 1975 - 1984 | Unknown | Unknown | Virus detection; Antigen detection | Qualitative | Month |
| Appak, 2019 [[20]](https://paperpile.com/c/zPIKrB/iCtM) | Turkey | Izmir | 27·1, 38·4 | 2011 - 2018 | outpatient; inpatient | ARI or ILI | Nucleic acid detection | Qualitative | Month |
| Arnott, 2011 [[21]](https://paperpile.com/c/zPIKrB/NNGa) | Cambodia | nationwide | 105, 12·6 | 2005 - 2009 | inpatient | ALRI | Nucleic acid detection | Qualitative | Month |
| Assink, 2009 [[22]](https://paperpile.com/c/zPIKrB/Z8Prv) | The Netherlands | nationwide | 5·3, 52·1 | 1998 - 2006 | Unknown | Unknown | Unknown | Threshold-based | Week |
| Azzari, 2021 [[23]](https://paperpile.com/c/zPIKrB/Ioh4) | Italy | Lombardy | 9·8, 45·5 | 2014 - 2018 | outpatient | ARI or ILI | Unknown | Qualitative | Month |
| Bakir, 1998 [[24]](https://paperpile.com/c/zPIKrB/PBuo) | Saudi Arabia | Riyadh | 46·7, 24·7 | 1993 - 1996 | inpatient | ARI or ILI | Virus detection; Antigen detection | Qualitative | Month |
| Balmaks, 2014 [[25]](https://paperpile.com/c/zPIKrB/Xgw6) | Latvia | Riga | 24·1, 56·9 | 2009 - 2012 | inpatient | ALRI | Nucleic acid detection | Qualitative | Week |
| Bandeira, 2022 [[26]](https://paperpile.com/c/zPIKrB/avkSI) | Portugal | nationwide | -9·2, 39·7 | 2015 - 2018 | inpatient | Unknown | Unknown | Qualitative | Week |
| Barbati, 2020 [[27]](https://paperpile.com/c/zPIKrB/uycl) | Italy | Tuscany | 11·2, 43·8 | 2014 - 2019 | inpatient | Unknown | Nucleic acid detection | Qualitative | Month |
| Bauman, 2007 [[28]](https://paperpile.com/c/zPIKrB/T5MU6) | USA | Central, Florida state | -81·9, 28·1 | 1999 - 2004 | Unknown | Unknown | Unknown | Threshold-based | Month |
| Bauman, 2007 [[28]](https://paperpile.com/c/zPIKrB/T5MU6) | USA | Florida state | -81·5, 27·7 | 1999 - 2004 | Unknown | Unknown | Unknown | Threshold-based | Month |
| Bauman, 2007 [[28]](https://paperpile.com/c/zPIKrB/T5MU6) | USA | North, Florida state | -80·2, 26·2 | 1999 - 2004 | Unknown | Unknown | Unknown | Threshold-based | Month |
| Bauman, 2007 [[28]](https://paperpile.com/c/zPIKrB/T5MU6) | USA | Southeast, Florida state | -80·4, 26·2 | 1999 - 2004 | Unknown | Unknown | Unknown | Threshold-based | Month |
| Bauman, 2007 [[28]](https://paperpile.com/c/zPIKrB/T5MU6) | USA | Southwest, Florida state | -81·9, 26·6 | 1999 - 2004 | Unknown | Unknown | Unknown | Threshold-based | Month |
| Baumeister, 2019 [[29]](https://paperpile.com/c/zPIKrB/StFDy) | Argentina | Central | -63·6, -38·4 | 2007 - 2016 | outpatient; inpatient | ARI or ILI; SARI; Clinical judgment | Antigen detection; Nucleic acid detection | Threshold-based | Week |
| Baumeister, 2019 [[29]](https://paperpile.com/c/zPIKrB/StFDy) | Argentina | Cuyo | -67·4, -35·1 | 2007 - 2016 | outpatient; inpatient | ARI or ILI; SARI; Clinical judgment | Antigen detection; Nucleic acid detection | Threshold-based | Week |
| Baumeister, 2019 [[29]](https://paperpile.com/c/zPIKrB/StFDy) | Argentina | nationwide | -63·6, -38·4 | 2007 - 2016 | outpatient; inpatient | ARI or ILI; SARI; Clinical judgment | Antigen detection; Nucleic acid detection | Threshold-based | Week |
| Baumeister, 2019 [[29]](https://paperpile.com/c/zPIKrB/StFDy) | Argentina | Northeast | -63·6, -38·4 | 2007 - 2016 | outpatient; inpatient | ARI or ILI; SARI; Clinical judgment | Antigen detection; Nucleic acid detection | Threshold-based | Week |
| Baumeister, 2019 [[29]](https://paperpile.com/c/zPIKrB/StFDy) | Argentina | Northwest | -63·6, -38·4 | 2007 - 2016 | outpatient; inpatient | ARI or ILI; SARI; Clinical judgment | Antigen detection; Nucleic acid detection | Threshold-based | Week |
| Baumeister, 2019 [[29]](https://paperpile.com/c/zPIKrB/StFDy) | Argentina | South | -63·6, -38·4 | 2007 - 2016 | outpatient; inpatient | ARI or ILI; SARI; Clinical judgment | Antigen detection; Nucleic acid detection | Threshold-based | Week |
| Berner, 2001 [[30]](https://paperpile.com/c/zPIKrB/EIBi) | Germany | Freiburg | 7·8, 48 | 1988 - 1998 | inpatient | ARI or ILI | Antigen detection | Qualitative | Month |
| Billard, 2022 [[31]](https://paperpile.com/c/zPIKrB/aUflC) | Brazil | nationwide | -51·9, -14·2 | 2017 - 2019 | Unknown | Unknown | Unknown | Coverage-based | Week |
| Billard, 2022 [[31]](https://paperpile.com/c/zPIKrB/aUflC) | Canada | nationwide | -106·3, 56·1 | 2017 - 2019 | Unknown | Unknown | Unknown | Coverage-based | Week |
| Billard, 2022 [[31]](https://paperpile.com/c/zPIKrB/aUflC) | Chile | nationwide | -71·5, -35·7 | 2017 - 2019 | Unknown | Unknown | Unknown | Coverage-based | Week |
| Billard, 2022 [[31]](https://paperpile.com/c/zPIKrB/aUflC) | France | nationwide | 2·7, 47·1 | 2017 - 2019 | Unknown | Unknown | Unknown | Coverage-based | Week |
| Billard, 2022 [[31]](https://paperpile.com/c/zPIKrB/aUflC) | Israel | nationwide | 34·9, 31 | 2017 - 2019 | Unknown | Unknown | Unknown | Coverage-based | Week |
| Billard, 2022 [[31]](https://paperpile.com/c/zPIKrB/aUflC) | Japan | nationwide | 138·3, 36·2 | 2017 - 2019 | Unknown | Unknown | Unknown | Coverage-based | Week |
| Billard, 2022 [[31]](https://paperpile.com/c/zPIKrB/aUflC) | South Africa | nationwide | 22·9, -30·6 | 2017 - 2019 | Unknown | Unknown | Unknown | Coverage-based | Week |
| Billard, 2022 [[31]](https://paperpile.com/c/zPIKrB/aUflC) | South Korea | nationwide | 127·8, 35·9 | 2017 - 2019 | Unknown | Unknown | Unknown | Coverage-based | Week |
| Billard, 2022 [[31]](https://paperpile.com/c/zPIKrB/aUflC) | Taiwan | nationwide | 121, 23·7 | 2017 - 2019 | Unknown | Unknown | Unknown | Coverage-based | Week |
| Billard, 2022 [[31]](https://paperpile.com/c/zPIKrB/aUflC) | The Netherlands | nationwide | 5·3, 52·1 | 2017 - 2019 | Unknown | Unknown | Unknown | Coverage-based | Week |
| Billard, 2022 [[31]](https://paperpile.com/c/zPIKrB/aUflC) | USA | nationwide | -95·7, 37·1 | 2017 - 2019 | Unknown | Unknown | Unknown | Coverage-based | Week |
| BMJ 1979* [[32]](https://paperpile.com/c/zPIKrB/qwlJ) | United Kingdom | Scotland | -4·2, 56·5 | 1971 - 1978 | Unknown | Unknown | Unknown | Qualitative | Month |
| Boron, 2008 [[33]](https://paperpile.com/c/zPIKrB/80EEw) | USA | Midwest | -93·1, 41·9 | 2004 - 2007 | outpatient; inpatient | Unknown | Virus detection; Antigen detection; Nucleic acid detection | Threshold-based | Week |
| Boron, 2008 [[33]](https://paperpile.com/c/zPIKrB/80EEw) | USA | nationwide | -95·7, 37·1 | 2004 - 2007 | outpatient; inpatient | Unknown | Virus detection; Antigen detection; Nucleic acid detection | Threshold-based | Week |
| Boron, 2008 [[33]](https://paperpile.com/c/zPIKrB/80EEw) | USA | Northeast | -74·2, 43·3 | 2004 - 2007 | outpatient; inpatient | Unknown | Virus detection; Antigen detection; Nucleic acid detection | Threshold-based | Week |
| Boron, 2008 [[33]](https://paperpile.com/c/zPIKrB/80EEw) | USA | South | -99, 32 | 2004 - 2007 | outpatient; inpatient | Unknown | Virus detection; Antigen detection; Nucleic acid detection | Threshold-based | Week |
| Boron, 2008 [[33]](https://paperpile.com/c/zPIKrB/80EEw) | USA | West | -111, 37·1 | 2004 - 2007 | outpatient; inpatient | Unknown | Virus detection; Antigen detection; Nucleic acid detection | Threshold-based | Week |
| Brini, 2020 [[34]](https://paperpile.com/c/zPIKrB/71Jr) | Tunisia | Sousse | 10·6, 35·8 | 2003 - 2015 | inpatient | ALRI | Antigen detection | Qualitative | Month |
| Brittain-Long, 2011 [[35]](https://paperpile.com/c/zPIKrB/jYh9) | Sweden | Gothenburg | 12, 57·7 | 2006 - 2009 | outpatient; inpatient | Unknown | Nucleic acid detection | Qualitative | Month |
| Broberg, 2018 [[36]](https://paperpile.com/c/zPIKrB/Alr49) | Denmark | nationwide | 10·9, 55·9 | 2010 - 2016 | outpatient; inpatient | ARI or ILI; Clinical judgment | Virus detection; Antibody detection; Antigen detection; Nucleic acid detection | Threshold-based | Week |
| Broberg, 2018 [[36]](https://paperpile.com/c/zPIKrB/Alr49) | Estonia | nationwide | 25·5, 59 | 2010 - 2016 | outpatient | ARI or ILI | Virus detection; Antibody detection; Antigen detection; Nucleic acid detection | Threshold-based | Week |
| Broberg, 2018 [[36]](https://paperpile.com/c/zPIKrB/Alr49) | Estonia | nationwide | 25·5, 59 | 2010 - 2016 | outpatient; inpatient | ARI or ILI | Virus detection; Antibody detection; Antigen detection; Nucleic acid detection | Threshold-based | Week |
| Broberg, 2018 [[36]](https://paperpile.com/c/zPIKrB/Alr49) | Europe | nationwide | 15·3, 54·5 | 2010 - 2016 | outpatient; inpatient | ARI or ILI; Clinical judgment | Virus detection; Antibody detection; Antigen detection; Nucleic acid detection | Threshold-based | Week |
| Broberg, 2018 [[36]](https://paperpile.com/c/zPIKrB/Alr49) | Europe | nationwide | 15·3, 54·5 | 2010 - 2016 | outpatient; inpatient | ARI or ILI; Clinical judgment | Virus detection; Antibody detection; Antigen detection; Nucleic acid detection | Threshold-based | Week |
| Broberg, 2018 [[36]](https://paperpile.com/c/zPIKrB/Alr49) | France | nationwide | 2·7, 47·1 | 2010 - 2016 | outpatient | ARI or ILI | Virus detection; Antibody detection; Antigen detection; Nucleic acid detection | Threshold-based | Week |
| Broberg, 2018 [[36]](https://paperpile.com/c/zPIKrB/Alr49) | France | nationwide | 2·7, 47·1 | 2010 - 2016 | outpatient; inpatient | ARI or ILI | Virus detection; Antibody detection; Antigen detection; Nucleic acid detection | Threshold-based | Week |
| Broberg, 2018 [[36]](https://paperpile.com/c/zPIKrB/Alr49) | Germany | nationwide | 9·7, 50·9 | 2010 - 2016 | outpatient | ARI or ILI | Virus detection; Antibody detection; Antigen detection; Nucleic acid detection | Threshold-based | Week |
| Broberg, 2018 [[36]](https://paperpile.com/c/zPIKrB/Alr49) | Germany | nationwide | 9·7, 50·9 | 2010 - 2016 | outpatient; inpatient | ARI or ILI | Virus detection; Antibody detection; Antigen detection; Nucleic acid detection | Threshold-based | Week |
| Broberg, 2018 [[36]](https://paperpile.com/c/zPIKrB/Alr49) | Iceland | nationwide | -21·1, 64·4 | 2010 - 2016 | outpatient; inpatient | Clinical judgment | Virus detection; Antibody detection; Antigen detection; Nucleic acid detection | Threshold-based | Week |
| Broberg, 2018 [[36]](https://paperpile.com/c/zPIKrB/Alr49) | Ireland | nationwide | -7·4, 53·1 | 2010 - 2016 | outpatient; inpatient | Clinical judgment | Virus detection; Antibody detection; Antigen detection; Nucleic acid detection | Threshold-based | Week |
| Broberg, 2018 [[36]](https://paperpile.com/c/zPIKrB/Alr49) | Latvia | nationwide | 24·4, 56·8 | 2010 - 2016 | outpatient; inpatient | Clinical judgment | Virus detection; Antibody detection; Antigen detection; Nucleic acid detection | Threshold-based | Week |
| Broberg, 2018 [[36]](https://paperpile.com/c/zPIKrB/Alr49) | Malta | nationwide | 14·5, 35·9 | 2010 - 2016 | outpatient; inpatient | ARI or ILI | Virus detection; Antibody detection; Antigen detection; Nucleic acid detection | Threshold-based | Week |
| Broberg, 2018 [[36]](https://paperpile.com/c/zPIKrB/Alr49) | Poland | nationwide | 19·3, 51·7 | 2010 - 2016 | outpatient; inpatient | ARI or ILI | Virus detection; Antibody detection; Antigen detection; Nucleic acid detection | Threshold-based | Week |
| Broberg, 2018 [[36]](https://paperpile.com/c/zPIKrB/Alr49) | Portugal | nationwide | -9·2, 39·7 | 2010 - 2016 | outpatient; inpatient | ARI or ILI | Virus detection; Antibody detection; Antigen detection; Nucleic acid detection | Threshold-based | Week |
| Broberg, 2018 [[36]](https://paperpile.com/c/zPIKrB/Alr49) | Slovenia | nationwide | 14·9, 46·2 | 2010 - 2016 | outpatient | ARI or ILI | Virus detection; Antibody detection; Antigen detection; Nucleic acid detection | Threshold-based | Week |
| Broberg, 2018 [[36]](https://paperpile.com/c/zPIKrB/Alr49) | Spain | nationwide | -3·3, 39·7 | 2010 - 2016 | outpatient; inpatient | Clinical judgment | Virus detection; Antibody detection; Antigen detection; Nucleic acid detection | Threshold-based | Week |
| Broberg, 2018 [[36]](https://paperpile.com/c/zPIKrB/Alr49) | Sweden | nationwide | 15·4, 58·9 | 2010 - 2016 | outpatient; inpatient | Clinical judgment | Virus detection; Antibody detection; Antigen detection; Nucleic acid detection | Threshold-based | Week |
| Broberg, 2018 [[36]](https://paperpile.com/c/zPIKrB/Alr49) | The Netherlands | nationwide | 5·3, 52·1 | 2010 - 2016 | outpatient | ARI or ILI | Virus detection; Antibody detection; Antigen detection; Nucleic acid detection | Threshold-based | Week |
| Broberg, 2018 [[36]](https://paperpile.com/c/zPIKrB/Alr49) | The Netherlands | nationwide | 5·3, 52·1 | 2010 - 2016 | outpatient; inpatient | ARI or ILI; Clinical judgment | Virus detection; Antibody detection; Antigen detection; Nucleic acid detection | Threshold-based | Week |
| Broberg, 2018 [[36]](https://paperpile.com/c/zPIKrB/Alr49) | United Kingdom | nationwide | -1·6, 52·7 | 2010 - 2016 | outpatient | ARI or ILI | Virus detection; Antibody detection; Antigen detection; Nucleic acid detection | Threshold-based | Week |
| Broberg, 2018 [[36]](https://paperpile.com/c/zPIKrB/Alr49) | United Kingdom | nationwide | -1·6, 52·7 | 2010 - 2016 | outpatient; inpatient | ARI or ILI | Virus detection; Antibody detection; Antigen detection; Nucleic acid detection | Threshold-based | Week |
| Bruden, 2015 [[37]](https://paperpile.com/c/zPIKrB/OpAug) | USA | YK Delta | -164·2, 62·9 | 1994 - 2012 | inpatient | ALRI | Virus detection; Antigen detection | Threshold-based | Week |
| Cai, 2022 [[38]](https://paperpile.com/c/zPIKrB/42Pvu) | Germany | nationwide | 9·7, 50·9 | 2011 - 2019 | outpatient | ARI or ILI | Nucleic acid detection | Threshold-based | Week |
| Caini, 2019 [[39]](https://paperpile.com/c/zPIKrB/WHKf) | Ecuador | nationwide | -78·2, -1·8 | 2010 - 2016 | outpatient; inpatient | ARI or ILI; SARI | Nucleic acid detection | Qualitative | Month |
| Caini, 2022 [[40]](https://paperpile.com/c/zPIKrB/DKVK) | Russia | Chita | 113·5, 52·1 | 2014 - 2019 | outpatient; inpatient | ARI or ILI; SARI | Nucleic acid detection | Qualitative | Month |
| Caini, 2022 [[40]](https://paperpile.com/c/zPIKrB/DKVK) | Russia | Kaliningrad | 20·5, 54·7 | 2014 - 2019 | outpatient; inpatient | ARI or ILI; SARI | Nucleic acid detection | Qualitative | Month |
| Caini, 2022 [[40]](https://paperpile.com/c/zPIKrB/DKVK) | Russia | Lipetsk | 39·6, 52·6 | 2014 - 2019 | outpatient; inpatient | ARI or ILI; SARI | Nucleic acid detection | Qualitative | Month |
| Caini, 2022 [[40]](https://paperpile.com/c/zPIKrB/DKVK) | Russia | St. Petersburg | 30·4, 59·9 | 2014 - 2019 | outpatient; inpatient | ARI or ILI; SARI | Nucleic acid detection | Qualitative | Month |
| Caini, 2022 [[40]](https://paperpile.com/c/zPIKrB/DKVK) | Russia | Vladivostok | 131·9, 43·1 | 2014 - 2019 | outpatient; inpatient | ARI or ILI; SARI | Nucleic acid detection | Qualitative | Month |
| Callahan, 2020 [[5]](https://paperpile.com/c/zPIKrB/o6J01) | USA | nationwide | -95·7, 37·1 | 2005 - 2018 | outpatient; inpatient | Unknown | Virus detection; Antigen detection; Nucleic acid detection | Model-based | Week |
| Callahan, 2020 [[5]](https://paperpile.com/c/zPIKrB/o6J01) | USA | nationwide | -95·7, 37·1 | 2005 - 2018 | outpatient; inpatient | Unknown | Virus detection; Antigen detection; Nucleic acid detection | Qualitative | Month |
| Callahan, 2020 [[5]](https://paperpile.com/c/zPIKrB/o6J01) | USA | Utah | -111·1, 39·3 | 2005 - 2018 | outpatient; inpatient | Unknown | Virus detection; Antigen detection; Nucleic acid detection | Model-based | Week |
| Calvo, 2010 [[41]](https://paperpile.com/c/zPIKrB/TCQq) | Spain | Leganés | -3·8, 40·3 | 2005 - 2008 | inpatient | ALRI | Nucleic acid detection | Qualitative | Month |
| Calvo, 2016 [[42]](https://paperpile.com/c/zPIKrB/PLZ0) | Spain | Leganés | -3·8, 40·3 | 2005 - 2013 | inpatient | ALRI | Nucleic acid detection | Qualitative | Month |
| Cattoir, 2018 [[43]](https://paperpile.com/c/zPIKrB/fNot) | Belgium | nationwide | 4·5, 50·5 | 2006 - 2016 | outpatient; inpatient | Unknown | Nucleic acid detection | Qualitative | Month |
| CDC, 2011 [[44]](https://paperpile.com/c/zPIKrB/u2aTv) | USA | Florida state | -81·5, 27·7 | 2007 - 2011 | Unknown | Unknown | Virus detection; Antigen detection; Nucleic acid detection | Threshold-based | Week |
| CDC, 2011 [[44]](https://paperpile.com/c/zPIKrB/u2aTv) | USA | HHS1 | 71·1, 42·4 | 2007 - 2011 | Unknown | Unknown | Virus detection; Antigen detection; Nucleic acid detection | Threshold-based | Week |
| CDC, 2011 [[44]](https://paperpile.com/c/zPIKrB/u2aTv) | USA | HHS10 | 122·3, 47·6 | 2007 - 2011 | Unknown | Unknown | Virus detection; Antigen detection; Nucleic acid detection | Threshold-based | Week |
| CDC, 2011 [[44]](https://paperpile.com/c/zPIKrB/u2aTv) | USA | HHS2 | 74, 40·7 | 2007 - 2011 | Unknown | Unknown | Virus detection; Antigen detection; Nucleic acid detection | Threshold-based | Week |
| CDC, 2011 [[44]](https://paperpile.com/c/zPIKrB/u2aTv) | USA | HHS3 | 75·2, 40 | 2007 - 2011 | Unknown | Unknown | Virus detection; Antigen detection; Nucleic acid detection | Threshold-based | Week |
| CDC, 2011 [[44]](https://paperpile.com/c/zPIKrB/u2aTv) | USA | HHS4 | 84·4, 33·8 | 2007 - 2011 | Unknown | Unknown | Virus detection; Antigen detection; Nucleic acid detection | Threshold-based | Week |
| CDC, 2011 [[44]](https://paperpile.com/c/zPIKrB/u2aTv) | USA | HHS5 | 87·6, 41·9 | 2007 - 2011 | Unknown | Unknown | Virus detection; Antigen detection; Nucleic acid detection | Threshold-based | Week |
| CDC, 2011 [[44]](https://paperpile.com/c/zPIKrB/u2aTv) | USA | HHS6 | 96·8, 32·8 | 2007 - 2011 | Unknown | Unknown | Virus detection; Antigen detection; Nucleic acid detection | Threshold-based | Week |
| CDC, 2011 [[44]](https://paperpile.com/c/zPIKrB/u2aTv) | USA | HHS7 | 94·6, 39·1 | 2007 - 2011 | Unknown | Unknown | Virus detection; Antigen detection; Nucleic acid detection | Threshold-based | Week |
| CDC, 2011 [[44]](https://paperpile.com/c/zPIKrB/u2aTv) | USA | HHS8 | 105, 39·7 | 2007 - 2011 | Unknown | Unknown | Virus detection; Antigen detection; Nucleic acid detection | Threshold-based | Week |
| CDC, 2011 [[44]](https://paperpile.com/c/zPIKrB/u2aTv) | USA | HHS9 | 122·4, 37·8 | 2007 - 2011 | Unknown | Unknown | Virus detection; Antigen detection; Nucleic acid detection | Threshold-based | Week |
| CDC, 2011 [[44]](https://paperpile.com/c/zPIKrB/u2aTv) | USA | nationwide | -95·7, 37·1 | 2007 - 2011 | Unknown | Unknown | Virus detection; Antigen detection; Nucleic acid detection | Threshold-based | Week |
| CDC, 2011 [[44]](https://paperpile.com/c/zPIKrB/u2aTv) | USA | nationwide | -95·7, 37·1 | 2007 - 2011 | Unknown | Unknown | Virus detection; Antigen detection; Nucleic acid detection | Threshold-based | Week |
| Chan, 1999 [[45]](https://paperpile.com/c/zPIKrB/o9nC) | Hong Kong | nationwide | 114·2, 22·3 | 1993 - 1997 | inpatient | SARI | Virus detection; Antigen detection | Qualitative | Month |
| Chan, 2002 [[46]](https://paperpile.com/c/zPIKrB/qofI) | Malaysia | nationwide | 102, 4·2 | 1982 - 1997 | inpatient | ALRI | Virus detection; Antigen detection | Qualitative | Month |
| Chan, 2015 [[47]](https://paperpile.com/c/zPIKrB/sdUN2) | Hong Kong | nationwide | 114·2, 22·3 | 1998 - 2012 | inpatient | ARI or ILI | Antigen detection | Threshold-based | Week |
| Chen, 2013 [[48]](https://paperpile.com/c/zPIKrB/A33W) | China | Suzhou | 120·6, 31·3 | 2001 - 2011 | inpatient | SARI | Nucleic acid detection | Qualitative | Month |
| Chew, 1998 [[49]](https://paperpile.com/c/zPIKrB/bzzz) | Singapore | nationwide | 103·8, 1·4 | 1990 - 1994 | outpatient; inpatient | Unknown | Virus detection; Antibody detection; Antigen detection | Qualitative | Month |
| Chi, 2011 [[50]](https://paperpile.com/c/zPIKrB/osdms) | Taiwan | nationwide | 121, 23·7 | 2004 - 2007 | inpatient | Unknown | Unknown | Qualitative | Month |
| Chittaganpitch, 2018 [[51]](https://paperpile.com/c/zPIKrB/RFay) | Thailand | nationwide | 101, 15·9 | 2010 - 2014 | outpatient; inpatient | ARI or ILI; SARI | Nucleic acid detection | Qualitative | Month |
| Choudhary, 2013 [[52]](https://paperpile.com/c/zPIKrB/H1R9) | India | nationwide | 79, 20·6 | 2009 - 2012 | outpatient; inpatient | ARI or ILI; SARI | Nucleic acid detection | Qualitative | Month |
| Choudhary, 2013 [[52]](https://paperpile.com/c/zPIKrB/H1R9) | India | Pune | 73·9, 18·5 | 2009 - 2012 | outpatient; inpatient | ARI or ILI; SARI | Nucleic acid detection | Qualitative | Month |
| Chu, 2022 [[53]](https://paperpile.com/c/zPIKrB/qC9rq) | China | Jinan | 117, 36·7 | 2016 - 2019 | inpatient | ALRI | Nucleic acid detection | Qualitative | Month |
| Cui, 2013 [[54]](https://paperpile.com/c/zPIKrB/IMnZ) | China | Beijing | 116·4, 39·9 | 2007 - 2012 | inpatient | ARI or ILI | Antigen detection | Qualitative | Month |
| Cui, 2016 [[55]](https://paperpile.com/c/zPIKrB/mcfA) | China | Eastern China | 104·2, 35·9 | 2009 - 2013 | inpatient | ALRI | Nucleic acid detection | Qualitative | Month |
| Cui, 2016 [[55]](https://paperpile.com/c/zPIKrB/mcfA) | China | nationwide | 104·2, 35·9 | 2009 - 2013 | inpatient | ALRI | Nucleic acid detection | Qualitative | Month |
| Darniot, 2018 [[56]](https://paperpile.com/c/zPIKrB/Jb43) | France | Burgundy | 4·4, 47·1 | 2011 - 2016 | inpatient | ARI or ILI | Antigen detection; Nucleic acid detection | Qualitative | Week |
| De Conto, 2019 [[57]](https://paperpile.com/c/zPIKrB/vNcVL) | Italy | Parma | 10·3, 44·8 | 2012 - 2015 | outpatient; inpatient | ARI or ILI | Antigen detection; Nucleic acid detection | Threshold-based | Month |
| De Silva, 1986 [[58]](https://paperpile.com/c/zPIKrB/zsbw) | Australia | Sydney | 151·2, -33·9 | 1979 - 1983 | inpatient | ARI or ILI | Antigen detection | Qualitative | Month |
| Dearden, 2018 [[59]](https://paperpile.com/c/zPIKrB/kuOZ) | South Africa | Pretoria | 28·2, -25·7 | 2013 - 2016 | outpatient; inpatient | ALRI | Antigen detection; Nucleic acid detection | Qualitative | Month |
| Do, 2011 [[60]](https://paperpile.com/c/zPIKrB/tFOs) | Vietnam | Ho Chi Minh | 106·6, 10·8 | 2004 - 2008 | inpatient | SARI | Nucleic acid detection | Qualitative | Month |
| Duppenthaler, 2003 [[61]](https://paperpile.com/c/zPIKrB/CpdqX) | Switzerland | Bern | 7·4, 46·9 | 1997 - 2001 | inpatient | Clinical judgment | Antigen detection | Qualitative | Week |
| Duppenthaler, 2003 [[61]](https://paperpile.com/c/zPIKrB/CpdqX) | Switzerland | Bern | 7·4, 46·9 | 1997 - 2001 | inpatient | Clinical judgment | Antigen detection | Qualitative | Week |
| Duppenthaler, 2003 [[61]](https://paperpile.com/c/zPIKrB/CpdqX) | Switzerland | nationwide | 8·2, 46·8 | 1988 - 1999 | outpatient; inpatient | Unknown | Unknown | Threshold-based | Week |
| Duppenthaler, 2003 [[61]](https://paperpile.com/c/zPIKrB/CpdqX) | Switzerland | nationwide | 8·2, 46·8 | 1988 - 1999 | outpatient; inpatient | Unknown | Unknown | Threshold-based | Week |
| Eidelman, 2009 [[62]](https://paperpile.com/c/zPIKrB/d1TO) | Israel | Jerusalem | 35·2, 31·8 | 2002 - 2007 | inpatient | ALRI | Antigen detection | Qualitative | Month |
| Eriksson, 2002 [[63]](https://paperpile.com/c/zPIKrB/j8Al7) | Sweden | Stockholm | 18·1, 59·3 | 1987 - 1998 | inpatient | Clinical judgment | Antigen detection | Qualitative | Month |
| Feng, 2014 [[64]](https://paperpile.com/c/zPIKrB/PY7d) | China | nationwide | 104·2, 35·9 | 2009 - 2013 | inpatient | ALRI | Nucleic acid detection | Qualitative | Month |
| Fergie, 2007 [[65]](https://paperpile.com/c/zPIKrB/o3kmc) | USA | nationwide | -95·7, 37·1 | 1996 - 2007 | Unknown | Unknown | Antigen detection;Virus detection | Threshold-based | Week |
| Fergie, 2007 [[65]](https://paperpile.com/c/zPIKrB/o3kmc) | USA | South | -99, 32 | 1996 - 2007 | Unknown | Unknown | Antigen detection;Virus detection | Threshold-based | Week |
| Fergie, 2007 [[65]](https://paperpile.com/c/zPIKrB/o3kmc) | USA | South Texas state | -99·9, 32 | 1996 - 2007 | Unknown | Unknown | Antigen detection;Virus detection | Threshold-based | Week |
| Fergie, 2007 [[65]](https://paperpile.com/c/zPIKrB/o3kmc) | USA | South Texas state | -99·9, 32 | 1996 - 2007 | Unknown | Unknown | Antigen detection;Virus detection | Threshold-based | Week |
| Ferrero, 2016 [[66]](https://paperpile.com/c/zPIKrB/YkTtU) | Argentina | Buenos Aires | -58·4, -34·6 | 1995 - 2014 | Unknown | Unknown | Unknown | Threshold-based | Week |
| Ferrero, 2016 [[66]](https://paperpile.com/c/zPIKrB/YkTtU) | Argentina | nationwide | -63·6, -38·4 | 1995 - 2014 | Unknown | Unknown | Unknown | Threshold-based | Week |
| Fjaerli, 2004 [[67]](https://paperpile.com/c/zPIKrB/NZeh) | Norway | Akershus | 11·4, 60 | 1993 - 2000 | inpatient | ALRI | Antigen detection | Qualitative | Month |
| Fleming, 1993 [[68]](https://paperpile.com/c/zPIKrB/4ENS) | United Kingdom | nationwide | -1·6, 52·7 | 1989 - 1992 | Unknown | Unknown | Unknown | Qualitative | Week |
| Fleming, 2005 [[69]](https://paperpile.com/c/zPIKrB/kKmQz) | United Kingdom | England | -1·2, 52·4 | 1989 - 2000 | Unknown | Unknown | Unknown | Threshold-based | Week |
| Freitas, 2013 [[70]](https://paperpile.com/c/zPIKrB/tQMa) | Brazil | Midwest | -51·9, -14·2 | 2000 - 2010 | Unknown | ARI or ILI | Antigen detection | Qualitative | Month |
| Freitas, 2013 [[70]](https://paperpile.com/c/zPIKrB/tQMa) | Brazil | North | -58·4, -2·1 | 2000 - 2010 | Unknown | ARI or ILI | Antigen detection | Qualitative | Month |
| Freitas, 2013 [[70]](https://paperpile.com/c/zPIKrB/tQMa) | Brazil | Northeast | -42·6, -10·1 | 2000 - 2010 | Unknown | ARI or ILI | Antigen detection | Qualitative | Month |
| Freitas, 2013 [[70]](https://paperpile.com/c/zPIKrB/tQMa) | Brazil | South | -49·3, -25·4 | 2000 - 2010 | Unknown | ARI or ILI | Antigen detection | Qualitative | Month |
| Freitas, 2013 [[70]](https://paperpile.com/c/zPIKrB/tQMa) | Brazil | Southeast | -46·2, -20·3 | 2000 - 2010 | Unknown | ARI or ILI | Antigen detection | Qualitative | Month |
| Fry, 2006 [[71]](https://paperpile.com/c/zPIKrB/rMNIq) | USA | nationwide | -95·7, 37·1 | 1990 - 2004 | Unknown | Unknown | Antigen detection | Threshold-based | Week |
| García-Arroyo, 2022 [[72]](https://paperpile.com/c/zPIKrB/WKCsT) | Spain | Barcelona | 2·2, 41·4 | 1997 - 2020 | Unknown | Unknown | Virus detection;Antigen detection;Nucleic acid detection | Qualitative | Month |
| Gentile, 2019 [[73]](https://paperpile.com/c/zPIKrB/23GWJ) | Argentina | Buenos Aires | -58·4, -34·6 | 2000 - 2017 | inpatient | ALRI | Antigen detection; Nucleic acid detection | Threshold-based | Week |
| Gil-Prieto, 2015 [[74]](https://paperpile.com/c/zPIKrB/LQaL) | Spain | nationwide | -3·3, 39·7 | 1997 - 2011 | inpatient | ALRI | Unknown | Qualitative | Month |
| Glatman-Freedman, 2020 [[75]](https://paperpile.com/c/zPIKrB/nwDvF) | Israel | nationwide | 34·9, 31 | 2000 - 2017 | inpatient | Unknown | Unknown | Threshold-based | Month |
| Goddard, 2007 [[76]](https://paperpile.com/c/zPIKrB/XvW5a) | United Kingdom | nationwide | -1·6, 52·7 | 1994 - 2004 | Unknown | Unknown | Antigen detection; Nucleic acid detection | Threshold-based | Week |
| Grilc, 2021 [[77]](https://paperpile.com/c/zPIKrB/1iYBm) | Slovenia | nationwide | 14·9, 46·2 | 2008 - 2018 | outpatient; inpatient | Unknown | Nucleic acid detection | Coverage-based | Week |
| Grilc, 2021 [[77]](https://paperpile.com/c/zPIKrB/1iYBm) | Slovenia | nationwide | 14·9, 46·2 | 2008 - 2018 | outpatient; inpatient | Unknown | Nucleic acid detection | Threshold-based | Week |
| Gunell, 2016 [[78]](https://paperpile.com/c/zPIKrB/1N0Q) | Finland | nationwide | 25·7, 61·9 | 2010 - 2014 | Unknown | ARI or ILI | Antigen detection | Qualitative | Week |
| Halasa, 2015 [[79]](https://paperpile.com/c/zPIKrB/JjTJ) | Hashemite Kingdom of Jordan | Amman | 35·9, 32 | 2010 - 2013 | inpatient | ARI or ILI | Nucleic acid detection | Qualitative | Month |
| Halstead, 1998 [[80]](https://paperpile.com/c/zPIKrB/Raleq) | USA | Florida state | -81·5, 27·7 | 1993 - 1996 | Unknown | Unknown | Antigen detection | Threshold-based | Month |
| Hampp, 2013 [[81]](https://paperpile.com/c/zPIKrB/7vWjg) | USA | California state | -119·4, 36·8 | 1999 - 2004 | inpatient | Unknown | Unknown | Threshold-based | Week |
| Hampp, 2013 [[81]](https://paperpile.com/c/zPIKrB/7vWjg) | USA | California state | -119·4, 36·8 | 1999 - 2004 | inpatient | Unknown | Unknown | Threshold-based | Week |
| Hampp, 2013 [[81]](https://paperpile.com/c/zPIKrB/7vWjg) | USA | Florida state | -81·5, 27·7 | 1999 - 2004 | inpatient | Unknown | Unknown | Threshold-based | Week |
| Hampp, 2013 [[81]](https://paperpile.com/c/zPIKrB/7vWjg) | USA | Florida state | -81·5, 27·7 | 1999 - 2004 | inpatient | Unknown | Unknown | Threshold-based | Week |
| Hampp, 2013 [[81]](https://paperpile.com/c/zPIKrB/7vWjg) | USA | Illinois state | -89·4, 40·6 | 1999 - 2004 | inpatient | Unknown | Unknown | Threshold-based | Week |
| Hampp, 2013 [[81]](https://paperpile.com/c/zPIKrB/7vWjg) | USA | Illinois state | -89·4, 40·6 | 1999 - 2004 | inpatient | Unknown | Unknown | Threshold-based | Week |
| Hampp, 2013 [[81]](https://paperpile.com/c/zPIKrB/7vWjg) | USA | Texas state | -99·9, 32 | 1999 - 2004 | inpatient | Unknown | Unknown | Threshold-based | Week |
| Hampp, 2013 [[81]](https://paperpile.com/c/zPIKrB/7vWjg) | USA | Texas state | -99·9, 32 | 1999 - 2004 | inpatient | Unknown | Unknown | Threshold-based | Week |
| Haynes, 2013 [[82]](https://paperpile.com/c/zPIKrB/GMmUc) | Bangladesh | Dhaka, Bogra, Barisal, Comilla and Kishoreganj | 90·4, 23·7 | 2004 - 2011 | outpatient; inpatient | ARI or ILI | Nucleic acid detection | Threshold-based | Month |
| Haynes, 2013 [[82]](https://paperpile.com/c/zPIKrB/GMmUc) | Egypt | Damanhour | 30·5, 31 | 2009 - 2012 | outpatient; inpatient | ARI or ILI | Nucleic acid detection | Threshold-based | Month |
| Haynes, 2013 [[82]](https://paperpile.com/c/zPIKrB/GMmUc) | Guatemala | Santa Rosa, Guateala and Quetzaltenango | -90·4, 14·2 | 2007 - 2011 | outpatient; inpatient | ARI or ILI | Nucleic acid detection | Threshold-based | Month |
| Haynes, 2013 [[82]](https://paperpile.com/c/zPIKrB/GMmUc) | Kenya | Lwak and Kibera | 36·8, -1·3 | 2007 - 2011 | outpatient; inpatient | ARI or ILI | Nucleic acid detection | Threshold-based | Month |
| Haynes, 2013 [[82]](https://paperpile.com/c/zPIKrB/GMmUc) | South Africa | Pretoria and Soweto | 27·9, -26·2 | 2006 - 2012 | inpatient | ARI or ILI | Nucleic acid detection | Threshold-based | Month |
| Haynes, 2013 [[82]](https://paperpile.com/c/zPIKrB/GMmUc) | Thailand | Nakhon Phanom and Sae Kaeo | 104·8, 17·4 | 2005 - 2011 | outpatient; inpatient | ARI or ILI | Nucleic acid detection | Threshold-based | Month |
| Haynes, 2016 [[83]](https://paperpile.com/c/zPIKrB/HrPoU) | USA | nationwide | -95·7, 37·1 | 2008 - 2014 | Unknown | Unknown | Antigen detection | Threshold-based | Week |
| He, 2014 [[84]](https://paperpile.com/c/zPIKrB/m7VoW) | China | Shenzhen | 114·1, 22·5 | 2007 - 2010 | inpatient | SARI | Nucleic acid detection | Qualitative | Month |
| Hendaus, 2018 [[85]](https://paperpile.com/c/zPIKrB/Lvqn) | Qatar | nationwide | 51·2, 25·4 | 2010 - 2012 | inpatient | ALRI | Nucleic acid detection | Qualitative | Month |
| Hervás, 2012 [[86]](https://paperpile.com/c/zPIKrB/e4Bx) | Spain | Mallorca | 3, 39·7 | 1995 - 2006 | inpatient | ALRI | Virus detection; Antigen detection | Qualitative | Month |
| Hibino, 2018 [[87]](https://paperpile.com/c/zPIKrB/Ii5N) | Japan | Okinawa | 127·7, 26·1 | 2012 - 2015 | outpatient | ARI or ILI | Antigen detection | Qualitative | Month |
| Hirsh, 2014 [[88]](https://paperpile.com/c/zPIKrB/CQ96) | Israel | nationwide | 34·9, 31 | 2005 - 2012 | inpatient | SARI | Antigen detection; Nucleic acid detection | Qualitative | Week |
| Hogan, 2016 [[89]](https://paperpile.com/c/zPIKrB/VsIIl) | Australia | Goldfields, Western Australia | 121·5, -30·8 | 2000 - 2013 | Unknown | Unknown | Virus detection; Antibody detection; Antigen detection; Nucleic acid detection | Qualitative | Month |
| Hogan, 2016 [[89]](https://paperpile.com/c/zPIKrB/VsIIl) | Australia | Great Southern, Western Australia | 121·6, -27·7 | 2000 - 2013 | Unknown | Unknown | Virus detection; Antibody detection; Antigen detection; Nucleic acid detection | Qualitative | Month |
| Hogan, 2016 [[89]](https://paperpile.com/c/zPIKrB/VsIIl) | Australia | Kimberley, Western Australia | 125·9, -17·3 | 2000 - 2013 | Unknown | Unknown | Virus detection; Antibody detection; Antigen detection; Nucleic acid detection | Qualitative | Month |
| Hogan, 2016 [[89]](https://paperpile.com/c/zPIKrB/VsIIl) | Australia | Metropoltan, Western Australia | 121·6, -27·7 | 2000 - 2013 | Unknown | Unknown | Virus detection; Antibody detection; Antigen detection; Nucleic acid detection | Qualitative | Month |
| Hogan, 2016 [[89]](https://paperpile.com/c/zPIKrB/VsIIl) | Australia | Midwest-Murchison, Western Australia | 116, -26·5 | 2000 - 2013 | Unknown | Unknown | Virus detection; Antibody detection; Antigen detection; Nucleic acid detection | Qualitative | Month |
| Hogan, 2016 [[89]](https://paperpile.com/c/zPIKrB/VsIIl) | Australia | Pilbara, Western Australia | 121·5, -21·6 | 2000 - 2013 | Unknown | Unknown | Virus detection; Antibody detection; Antigen detection; Nucleic acid detection | Qualitative | Month |
| Hogan, 2016 [[89]](https://paperpile.com/c/zPIKrB/VsIIl) | Australia | South West, Western Australia | 118, -32 | 2000 - 2013 | Unknown | Unknown | Virus detection; Antibody detection; Antigen detection; Nucleic acid detection | Qualitative | Month |
| Hogan, 2016 [[89]](https://paperpile.com/c/zPIKrB/VsIIl) | Australia | Wheatbelt, Western Australia | 118·1, -32 | 2000 - 2013 | Unknown | Unknown | Virus detection; Antibody detection; Antigen detection; Nucleic acid detection | Qualitative | Month |
| [[90]](https://paperpile.com/c/zPIKrB/ktSc), 2019 [[90]](https://paperpile.com/c/zPIKrB/ktSc) | South Africa | nationwide | 22·9, -30·6 | 2006 - 2008 | inpatient | Unknown | Virus detection; Antigen detection | Qualitative | Month |
| Horton, 2017 [[91]](https://paperpile.com/c/zPIKrB/T8rb) | Egypt, Jordan, Oman, Qatar and Yemen | nationwide | 44·8, 24·3 | 2007 - 2014 | inpatient | SARI | Nucleic acid detection | Qualitative | Month |
| Houspie, 2013 [[92]](https://paperpile.com/c/zPIKrB/hReV) | Belgium | nationwide | 4·5, 50·5 | 2006 - 2010 | Unknown | ARI or ILI | Antigen detection | Qualitative | Month |
| Hsu, 2014 [[93]](https://paperpile.com/c/zPIKrB/I3rwW) | Taiwan | greater Taipei metropolitan area | 121·5, 25·1 | 2000 - 2010 | inpatient | ALRI | Virus detection; Antigen detection | Threshold-based | Month |
| Hu, 2017 [[94]](https://paperpile.com/c/zPIKrB/8SKn) | China | Chengdu | 104·1, 30·6 | 2009 - 2014 | inpatient | ARI or ILI | Nucleic acid detection | Qualitative | Month |
| Huang, 2001 [[95]](https://paperpile.com/c/zPIKrB/3Gde) | Taiwan | Northern Taiwan | 122, 25 | 1995 - 1999 | inpatient | ALRI | Antigen detection | Qualitative | Month |
| Huang, 2020 [[96]](https://paperpile.com/c/zPIKrB/5Q3h) | China | Guangzhou | 113·3, 23·1 | 2009 - 2018 | outpatient; inpatient | ARI or ILI | Nucleic acid detection | Qualitative | Month |
| Irmen, 2000 [[97]](https://paperpile.com/c/zPIKrB/xcSo5) | USA | Bismarck | -101, 46·8 | 1987 - 1998 | outpatient; inpatient | Unknown | Virus detection; Antigen detection | Qualitative | Month |
| Jepsen, 2018 [[98]](https://paperpile.com/c/zPIKrB/EaElK) | Denmark | nationwide | 10·9, 55·9 | 2010 - 2015 | inpatient | Unknown | Unknown | Qualitative | Month |
| Jiang, 2023 [[99]](https://paperpile.com/c/zPIKrB/UJyei) | China | Beijing | 116·4, 39·9 | 4291 - 2021 | outpatient; inpatient | ARI or ILI | Nucleic acid detection | Threshold-based | Month |
| Jin, 2012 [[100]](https://paperpile.com/c/zPIKrB/9VLa) | China | Lanzhou | 103·8, 36·1 | 2006 - 2009 | inpatient | ALRI | Nucleic acid detection | Qualitative | Month |
| Kaneko, 2002 [[101]](https://paperpile.com/c/zPIKrB/KPEg) | Japan | Shizuoka | 138·4, 35 | 1997 - 2000 | inpatient | ALRI | Antibody detection; Antigen detection | Qualitative | Month |
| Karron, 1999 [[102]](https://paperpile.com/c/zPIKrB/YOac) | USA | YK Delta | -164·2, 62·9 | 1993 - 1996 | inpatient | ARI or ILI | Virus detection; Antigen detection | Qualitative | Month |
| Khor, 2012 [[103]](https://paperpile.com/c/zPIKrB/IIXB) | Malaysia | Kuala Lumpur | 101·7, 3·1 | 1982 - 2008 | inpatient | ARI or ILI | Virus detection; Antigen detection | Qualitative | Month |
| Korsun, 2019 [[104]](https://paperpile.com/c/zPIKrB/zVld) | Bulgaria | nationwide | 25·5, 42·7 | 2015 - 2018 | outpatient; inpatient | ALRI | Nucleic acid detection | Qualitative | Month |
| Kyeyagalire, 2014 [[105]](https://paperpile.com/c/zPIKrB/H2UB) | South Africa | nationwide | 22·9, -30·6 | 2007 - 2012 | Unknown | Unknown | Unknown | Qualitative | Month |
| Lagacé-Wiens, 2021 [[106]](https://paperpile.com/c/zPIKrB/vDVJf) | Canada | nationwide | -106·3, 56·1 | 2010 - 2020 | outpatient; inpatient | ARI or ILI | Virus detection; Antigen detection; Nucleic acid detection | Threshold-based | Week |
| Lam, 2019 [[107]](https://paperpile.com/c/zPIKrB/WnPPa) | Australia | Brisbane | 153, -27·5 | 2010 - 2015 | outpatient; inpatient | Unknown | Nucleic acid detection | Model-based | Month |
| Lam, 2019 [[107]](https://paperpile.com/c/zPIKrB/WnPPa) | Australia | Sydney | 151·2, -33·9 | 2010 - 2015 | outpatient; inpatient | Unknown | Nucleic acid detection | Model-based | Month |
| Lam, 2019 [[107]](https://paperpile.com/c/zPIKrB/WnPPa) | Canada | Edmonton | -113, 53·5 | 2010 - 2015 | inpatient | Unknown | Nucleic acid detection | Model-based | Month |
| Lam, 2019 [[107]](https://paperpile.com/c/zPIKrB/WnPPa) | Canada | Halifax | -63·1, 44·9 | 2010 - 2015 | outpatient; inpatient | Unknown | Nucleic acid detection | Model-based | Month |
| Lam, 2019 [[107]](https://paperpile.com/c/zPIKrB/WnPPa) | Canada | Vancouver | -123, 49·3 | 2010 - 2015 | outpatient; inpatient | Unknown | Nucleic acid detection | Model-based | Month |
| Lam, 2019 [[107]](https://paperpile.com/c/zPIKrB/WnPPa) | Finland | Turku | 22·3, 60·5 | 2010 - 2015 | outpatient; inpatient | Unknown | Nucleic acid detection | Model-based | Month |
| Lam, 2019 [[107]](https://paperpile.com/c/zPIKrB/WnPPa) | Hong Kong | nationwide | 114·2, 22·3 | 2010 - 2015 | outpatient; inpatient | Unknown | Antigen detection | Model-based | Month |
| Lam, 2019 [[107]](https://paperpile.com/c/zPIKrB/WnPPa) | Japan | Sendai | 141, 38·3 | 2010 - 2015 | outpatient | Unknown | Virus detection; Antigen detection | Model-based | Month |
| Lam, 2019 [[107]](https://paperpile.com/c/zPIKrB/WnPPa) | Mongolia | Ulaanbaatar | 107, 47·9 | 2010 - 2015 | outpatient; inpatient | Unknown | Nucleic acid detection | Model-based | Month |
| Lam, 2019 [[107]](https://paperpile.com/c/zPIKrB/WnPPa) | New Zealand | Canterbury | 171, -43·8 | 2010 - 2015 | outpatient; inpatient | Unknown | Nucleic acid detection | Model-based | Month |
| Lam, 2019 [[107]](https://paperpile.com/c/zPIKrB/WnPPa) | The Netherlands | Rotterdam | 4·5, 51·9 | 2010 - 2015 | outpatient; inpatient | Unknown | Nucleic acid detection | Model-based | Month |
| Lam, 2019 [[107]](https://paperpile.com/c/zPIKrB/WnPPa) | United Kingdom | Cambridge | 0·1, 52·2 | 2010 - 2015 | inpatient | Unknown | Nucleic acid detection | Model-based | Month |
| Lam, 2019 [[107]](https://paperpile.com/c/zPIKrB/WnPPa) | United Kingdom | Leicester | -1·1, 52·6 | 2010 - 2015 | inpatient | Unknown | Nucleic acid detection | Model-based | Month |
| Leecaster, 2011 [[108]](https://paperpile.com/c/zPIKrB/nwSk) | USA | Salt Lake County | -112, 40·6 | 2001 - 2008 | outpatient; inpatient | ARI or ILI | Antigen detection; Nucleic acid detection | Qualitative | Month |
| Li, 2019 [[3]](https://paperpile.com/c/zPIKrB/BQ74y) | Argentina | Buenos Aires | -58·4, -34·6 | 1998 - 2002 | inpatient | ALRI | Antigen detection | Coverage-based | Month |
| Li, 2019 [[3]](https://paperpile.com/c/zPIKrB/BQ74y) | Argentina | nationwide | -63·6, -38·4 | 2010 - 2017 | Unknown | Unknown | Unknown | Coverage-based | Month |
| Li, 2019 [[3]](https://paperpile.com/c/zPIKrB/BQ74y) | Aruba | nationwide | -70, 12·5 | 2010 - 2017 | Unknown | Unknown | Unknown | Coverage-based | Month |
| Li, 2019 [[3]](https://paperpile.com/c/zPIKrB/BQ74y) | Austria | Vienna | 16·4, 48·2 | 2000 - 2007 | inpatient | ARI or ILI | Nucleic acid detection | Coverage-based | Month |
| Li, 2019 [[3]](https://paperpile.com/c/zPIKrB/BQ74y) | Bardados | nationwide | -59·5, 13·2 | 2010 - 2017 | Unknown | Unknown | Unknown | Coverage-based | Month |
| Li, 2019 [[3]](https://paperpile.com/c/zPIKrB/BQ74y) | Belgium | Leuven | 4·7, 50·9 | 2011 - 2016 | inpatient | ARI or ILI | Nucleic acid detection | Coverage-based | Month |
| Li, 2019 [[3]](https://paperpile.com/c/zPIKrB/BQ74y) | Bolivia | nationwide | -63·6, -16·3 | 2010 - 2017 | Unknown | Unknown | Unknown | Coverage-based | Month |
| Li, 2019 [[3]](https://paperpile.com/c/zPIKrB/BQ74y) | Brazil | Fortaleza | -38·5, -3·7 | 2006 - 2008 | Unknown | ARI or ILI | Antigen detection | Coverage-based | Month |
| Li, 2019 [[3]](https://paperpile.com/c/zPIKrB/BQ74y) | Brazil | nationwide | -51·9, -14·2 | 2010 - 2017 | Unknown | Unknown | Unknown | Coverage-based | Month |
| Li, 2019 [[3]](https://paperpile.com/c/zPIKrB/BQ74y) | Brazil | Salvador | -38·5, -13 | 4005 - 4154 | outpatient; inpatient | ARI or ILI | Nucleic acid detection | Coverage-based | Month |
| Li, 2019 [[3]](https://paperpile.com/c/zPIKrB/BQ74y) | Canada | nationwide | -106·3, 56·1 | 2008 - 2014 | Unknown | Unknown | Unknown | Coverage-based | Month |
| Li, 2019 [[3]](https://paperpile.com/c/zPIKrB/BQ74y) | Canada | Nova Scotia | -63·7, 44·7 | 2005 - 2008 | outpatient; inpatient | ARI or ILI | Virus detection; Antigen detection; Nucleic acid detection | Coverage-based | Month |
| Li, 2019 [[3]](https://paperpile.com/c/zPIKrB/BQ74y) | Chile | nationwide | -71·5, -35·7 | 2010 - 2017 | Unknown | Unknown | Unknown | Coverage-based | Month |
| Li, 2019 [[3]](https://paperpile.com/c/zPIKrB/BQ74y) | Colombia | nationwide | -74·3, 4·6 | 2010 - 2017 | Unknown | Unknown | Unknown | Coverage-based | Month |
| Li, 2019 [[3]](https://paperpile.com/c/zPIKrB/BQ74y) | Croatia | Zagreb County | 16·4, 45·9 | 1994 - 2005 | inpatient | ARI or ILI | Virus detection; Antigen detection | Coverage-based | Month |
| Li, 2019 [[3]](https://paperpile.com/c/zPIKrB/BQ74y) | Cuba | nationwide | -77·8, 21·5 | 2010 - 2017 | Unknown | Unknown | Unknown | Coverage-based | Month |
| Li, 2019 [[3]](https://paperpile.com/c/zPIKrB/BQ74y) | Dominica | nationwide | -61·4, 15·4 | 2010 - 2017 | Unknown | Unknown | Unknown | Coverage-based | Month |
| Li, 2019 [[3]](https://paperpile.com/c/zPIKrB/BQ74y) | Dominican Republic | nationwide | -70·2, 18·7 | 2010 - 2017 | Unknown | Unknown | Unknown | Coverage-based | Month |
| Li, 2019 [[3]](https://paperpile.com/c/zPIKrB/BQ74y) | Ecuador | nationwide | -78·2, -1·8 | 2010 - 2017 | Unknown | Unknown | Unknown | Coverage-based | Month |
| Li, 2019 [[3]](https://paperpile.com/c/zPIKrB/BQ74y) | Egypt | nationwide | 30·8, 26·8 | 2007 - 2014 | inpatient | SARI | Nucleic acid detection | Coverage-based | Month |
| Li, 2019 [[3]](https://paperpile.com/c/zPIKrB/BQ74y) | EI Salvador | nationwide | -88·9, 13·8 | 2010 - 2017 | Unknown | Unknown | Unknown | Coverage-based | Month |
| Li, 2019 [[3]](https://paperpile.com/c/zPIKrB/BQ74y) | Gambia | Banjul | -16·6, 13·5 | 1993 - 2002 | inpatient | SARI | Antigen detection | Coverage-based | Month |
| Li, 2019 [[3]](https://paperpile.com/c/zPIKrB/BQ74y) | Germany | Freiburg | 7·8, 48 | 1988 - 1999 | inpatient | ARI or ILI | Antigen detection | Coverage-based | Month |
| Li, 2019 [[3]](https://paperpile.com/c/zPIKrB/BQ74y) | Germany | Kiel | 10·1, 54·3 | 1994 - 2001 | inpatient | SARI | Antigen detection; Nucleic acid detection | Coverage-based | Month |
| Li, 2019 [[3]](https://paperpile.com/c/zPIKrB/BQ74y) | Germany | Stuttgart | 9·2, 48·8 | 1996 - 2004 | outpatient; inpatient | Clinical judgment | Antigen detection; Nucleic acid detection | Coverage-based | Month |
| Li, 2019 [[3]](https://paperpile.com/c/zPIKrB/BQ74y) | Greece | Athens | 23·7, 37·7 | 2002 - 2013 | inpatient | SARI | Antigen detection | Coverage-based | Month |
| Li, 2019 [[3]](https://paperpile.com/c/zPIKrB/BQ74y) | Guatemala | nationwide | -90·2, 15·8 | 2010 - 2017 | Unknown | Unknown | Unknown | Coverage-based | Month |
| Li, 2019 [[3]](https://paperpile.com/c/zPIKrB/BQ74y) | Hashemite Kingdom of Jordan | nationwide | 36·2, 30·6 | 2007 - 2014 | inpatient | SARI | Nucleic acid detection | Coverage-based | Month |
| Li, 2019 [[3]](https://paperpile.com/c/zPIKrB/BQ74y) | Honduras | nationwide | -86·2, 15·2 | 2010 - 2017 | Unknown | Unknown | Unknown | Coverage-based | Month |
| Li, 2019 [[3]](https://paperpile.com/c/zPIKrB/BQ74y) | Latvia | Riga | 24·1, 56·9 | 2009 - 2012 | inpatient | ALRI | Nucleic acid detection | Coverage-based | Month |
| Li, 2019 [[3]](https://paperpile.com/c/zPIKrB/BQ74y) | Oman | nationwide | 56, 21·5 | 2007 - 2014 | inpatient | SARI | Nucleic acid detection | Coverage-based | Month |
| Li, 2019 [[3]](https://paperpile.com/c/zPIKrB/BQ74y) | Pakistan | Karachi | 67, 24·9 | 2009 - 2012 | inpatient | ARI or ILI | Nucleic acid detection | Coverage-based | Month |
| Li, 2019 [[3]](https://paperpile.com/c/zPIKrB/BQ74y) | Qatar | Doha | 51·5, 25·3 | 1996 - 1998 | inpatient | Unknown | Antigen detection | Coverage-based | Month |
| Li, 2019 [[3]](https://paperpile.com/c/zPIKrB/BQ74y) | Spain | Leganés | -3·8, 40·3 | 2005 - 2013 | inpatient | ALRI | Nucleic acid detection | Coverage-based | Month |
| Li, 2019 [[3]](https://paperpile.com/c/zPIKrB/BQ74y) | Spain | Mallorca | 3, 39·7 | 1995 - 2006 | inpatient | ALRI | Virus detection; Antigen detection | Coverage-based | Month |
| Li, 2019 [[3]](https://paperpile.com/c/zPIKrB/BQ74y) | Spain | Sacyl | 41·7, -4·7 | 1992 - 2004 | inpatient | ALRI | Antigen detection | Coverage-based | Month |
| Li, 2019 [[3]](https://paperpile.com/c/zPIKrB/BQ74y) | Sweden | Gothenburg | 12, 57·7 | 2010 - 2013 | outpatient; inpatient | ARI or ILI | Nucleic acid detection | Coverage-based | Month |
| Li, 2019 [[3]](https://paperpile.com/c/zPIKrB/BQ74y) | Switzerland | Basel | 7·6, 47·6 | 2004 - 2008 | outpatient; inpatient | ARI or ILI | Nucleic acid detection | Coverage-based | Month |
| Li, 2019 [[3]](https://paperpile.com/c/zPIKrB/BQ74y) | Switzerland | Bern | 7·4, 46·9 | 1998 - 2010 | inpatient | ARI or ILI | Antigen detection | Coverage-based | Month |
| Li, 2019 [[3]](https://paperpile.com/c/zPIKrB/BQ74y) | United Kingdom | England | -1·2, 52·4 | 2007 - 2012 | inpatient | Unknown | Unknown | Coverage-based | Month |
| Li, 2022 [[109]](https://paperpile.com/c/zPIKrB/68Em7) | Denmark | nationwide | 10·9, 55·9 | 2012 - 2019 | Unknown | ARI or ILI; Clinical judgment | Unknown | Coverage-based | Week |
| Li, 2022 [[109]](https://paperpile.com/c/zPIKrB/68Em7) | Estonia | nationwide | 25·5, 59 | 2012 - 2018 | Unknown | ARI or ILI | Unknown | Coverage-based | Week |
| Li, 2022 [[109]](https://paperpile.com/c/zPIKrB/68Em7) | Germany | nationwide | 9·7, 50·9 | 2013 - 2019 | Unknown | ARI or ILI | Unknown | Coverage-based | Week |
| Li, 2022 [[109]](https://paperpile.com/c/zPIKrB/68Em7) | Germany | nationwide | 9·7, 50·9 | 2013 - 2019 | Unknown | ARI or ILI | Unknown | Coverage-based | Week |
| Li, 2022 [[109]](https://paperpile.com/c/zPIKrB/68Em7) | Ireland | nationwide | -7·4, 53·1 | 2012 - 2019 | Unknown | Clinical judgment | Unknown | Coverage-based | Week |
| Li, 2022 [[109]](https://paperpile.com/c/zPIKrB/68Em7) | Ireland | nationwide | -7·4, 53·1 | 2014 - 2019 | Unknown | ARI or ILI | Unknown | Coverage-based | Week |
| Li, 2022 [[109]](https://paperpile.com/c/zPIKrB/68Em7) | Poland | nationwide | 19·3, 51·7 | 2012 - 2019 | Unknown | ARI or ILI | Unknown | Coverage-based | Week |
| Li, 2022 [[109]](https://paperpile.com/c/zPIKrB/68Em7) | Portugal | nationwide | -9·2, 39·7 | 2012 - 2017 | Unknown | ARI or ILI; Clinical judgment | Unknown | Coverage-based | Week |
| Li, 2022 [[109]](https://paperpile.com/c/zPIKrB/68Em7) | Slovenia | nationwide | 14·9, 46·2 | 2010 - 2019 | Unknown | Clinical judgment | Unknown | Coverage-based | Week |
| Li, 2022 [[109]](https://paperpile.com/c/zPIKrB/68Em7) | Slovenia | nationwide | 14·9, 46·2 | 2012 - 2019 | Unknown | ARI or ILI | Unknown | Coverage-based | Week |
| Li, 2022 [[109]](https://paperpile.com/c/zPIKrB/68Em7) | Spain | nationwide | -3·3, 39·7 | 2012 - 2019 | Unknown | Clinical judgment | Unknown | Coverage-based | Week |
| Li, 2022 [[109]](https://paperpile.com/c/zPIKrB/68Em7) | The Netherlands | nationwide | 5·3, 52·1 | 2010 - 2019 | Unknown | Clinical judgment | Unknown | Coverage-based | Week |
| Li, 2022 [[109]](https://paperpile.com/c/zPIKrB/68Em7) | The Netherlands | nationwide | 5·3, 52·1 | 2012 - 2019 | Unknown | ARI or ILI | Unknown | Coverage-based | Week |
| Li, 2022 [[109]](https://paperpile.com/c/zPIKrB/68Em7) | United Kingdom | nationwide | -1·6, 52·7 | 2012 - 2019 | Unknown | ARI or ILI | Unknown | Coverage-based | Week |
| Li, 2022 [[109]](https://paperpile.com/c/zPIKrB/68Em7) | United Kingdom | nationwide | -1·6, 52·7 | 2012 - 2019 | Unknown | ARI or ILI | Unknown | Coverage-based | Week |
| Light, 2007 [[110]](https://paperpile.com/c/zPIKrB/VCs9C) | USA | Southeast, Florida state | -80·4, 26·2 | 2003 - 2006 | outpatient | ALRI | Unknown | Threshold-based | Month |
| Light, 2008 [[111]](https://paperpile.com/c/zPIKrB/XkHEI) | USA | Central, Florida state | -81·9, 28·1 | 2001 - 2004 | Unknown | Unknown | Virus detection; Nucleic acid detection | Threshold-based | Month |
| Light, 2008 [[111]](https://paperpile.com/c/zPIKrB/XkHEI) | USA | North, Florida state | -80·2, 26·2 | 2001 - 2004 | Unknown | Unknown | Virus detection; Nucleic acid detection | Threshold-based | Month |
| Light, 2008 [[111]](https://paperpile.com/c/zPIKrB/XkHEI) | USA | Northwest, Florida state | -86·5, 30·5 | 2001 - 2004 | Unknown | Unknown | Virus detection; Nucleic acid detection | Threshold-based | Month |
| Light, 2008 [[111]](https://paperpile.com/c/zPIKrB/XkHEI) | USA | Southeast, Florida state | -80·4, 26·2 | 2001 - 2004 | Unknown | Unknown | Virus detection; Nucleic acid detection | Threshold-based | Month |
| Light, 2008 [[111]](https://paperpile.com/c/zPIKrB/XkHEI) | USA | Southwest, Florida state | -81·9, 26·6 | 2001 - 2004 | Unknown | Unknown | Virus detection; Nucleic acid detection | Threshold-based | Month |
| Liu, 2014 [[112]](https://paperpile.com/c/zPIKrB/CEqg) | China | nationwide | 104·2, 35·9 | 2009 - 2012 | outpatient | ARI or ILI | Nucleic acid detection | Qualitative | Month |
| Liu, 2014 [[112]](https://paperpile.com/c/zPIKrB/CEqg) | China | Shanghai | 121·5, 31·2 | 2009 - 2012 | outpatient | ARI or ILI | Nucleic acid detection | Qualitative | Month |
| Liu, 2018 [[113]](https://paperpile.com/c/zPIKrB/Sq1i) | China | Shanghai | 121·5, 31·2 | 2013 - 2015 | inpatient | ALRI | Antigen detection | Qualitative | Month |
| Liu, 2019 [[114]](https://paperpile.com/c/zPIKrB/rm9oD) | China | Guangzhou | 113·3, 23·1 | 2009 - 2016 | inpatient | ARI or ILI | Nucleic acid detection | Qualitative | Month |
| Loconsole, 2022 [[115]](https://paperpile.com/c/zPIKrB/1S5Pg) | Italy | Bari | 16·9, 41·4 | 2017 - 2020 | inpatient | Unknown | Nucleic acid detection | Qualitative | Month |
| Loh, 2011 [[116]](https://paperpile.com/c/zPIKrB/yLS8) | Singapore | nationwide | 103·8, 1·4 | 2003 - 2008 | outpatient; inpatient | ARI or ILI | Antigen detection | Qualitative | Month |
| Low, 2022 [[117]](https://paperpile.com/c/zPIKrB/8OXnW) | Malaysia | nationwide | 102, 4·2 | 2015 - 2019 | Unknown | ARI or ILI | Nucleic acid detection | Qualitative | Month |
| Lu, 2015 [[118]](https://paperpile.com/c/zPIKrB/UFDf) | China | Suzhou | 120·6, 31·3 | 2010 - 2014 | inpatient | ALRI | Antigen detection; Nucleic acid detection | Qualitative | Month |
| Lumley, 2022 [[119]](https://paperpile.com/c/zPIKrB/L8WR) | United Kingdom | Oxford and Banbury | -1·3, 52·1 | 2016 - 2019 | outpatient; inpatient | ARI or ILI; Clinical judgment | Nucleic acid detection | Qualitative | Month |
| Luo, 2022 [[120]](https://paperpile.com/c/zPIKrB/RCZsO) | China | Beijing | 116·4, 39·9 | 2015 - 2019 | outpatient; inpatient | ALRI;ARI or ILI | Nucleic acid detection | Qualitative | Month |
| Mak, 2012 [[121]](https://paperpile.com/c/zPIKrB/J6R1R) | Hong Kong | nationwide | 114·2, 22·3 | 2004 - 2011 | Unknown | Unknown | Virus detection | Qualitative | Month |
| Martin, 1978 [[122]](https://paperpile.com/c/zPIKrB/Fz7X) | United Kingdom | Newcastle | -1·6, 55 | 1971 - 1977 | inpatient | SARI | Virus detection; Antigen detection | Qualitative | Month |
| McCracken, 2014 [[123]](https://paperpile.com/c/zPIKrB/RKhU) | Guatemala | nationwide | -90·2, 15·8 | 2007 - 2012 | inpatient | ARI or ILI | Nucleic acid detection | Qualitative | Month |
| McGuiness, 2014 [[124]](https://paperpile.com/c/zPIKrB/aBsPn) | USA | Florida state | -81·5, 27·7 | 2007 - 2012 | Unknown | Unknown | Virus detection; Antigen detection; Nucleic acid detection | Threshold-based | Week |
| McGuiness, 2014 [[124]](https://paperpile.com/c/zPIKrB/aBsPn) | USA | Midwest | -93·1, 41·9 | 2007 - 2012 | Unknown | Unknown | Virus detection; Antigen detection; Nucleic acid detection | Threshold-based | Week |
| McGuiness, 2014 [[124]](https://paperpile.com/c/zPIKrB/aBsPn) | USA | nationwide | -95·7, 37·1 | 2007 - 2012 | Unknown | Unknown | Virus detection; Antigen detection; Nucleic acid detection | Threshold-based | Week |
| McGuiness, 2014 [[124]](https://paperpile.com/c/zPIKrB/aBsPn) | USA | nationwide | -95·7, 37·1 | 2007 - 2012 | Unknown | Unknown | Virus detection; Antigen detection; Nucleic acid detection | Threshold-based | Week |
| McGuiness, 2014 [[124]](https://paperpile.com/c/zPIKrB/aBsPn) | USA | Northeast | -74·2, 43·3 | 2007 - 2012 | Unknown | Unknown | Virus detection; Antigen detection; Nucleic acid detection | Threshold-based | Week |
| McGuiness, 2014 [[124]](https://paperpile.com/c/zPIKrB/aBsPn) | USA | South | -99, 32 | 2007 - 2012 | Unknown | Unknown | Virus detection; Antigen detection; Nucleic acid detection | Threshold-based | Week |
| McGuiness, 2014 [[124]](https://paperpile.com/c/zPIKrB/aBsPn) | USA | West | -111, 37·1 | 2007 - 2012 | Unknown | Unknown | Virus detection; Antigen detection; Nucleic acid detection | Threshold-based | Week |
| Meerhoff, 2009 [[125]](https://paperpile.com/c/zPIKrB/br0sv) | The Netherlands | nationwide | 5·3, 52·1 | 1998 - 2005 | inpatient | ALRI | Virus detection; Antigen detection; Nucleic acid detection | Threshold-based | Week |
| Meerhoff, 2009 [[125]](https://paperpile.com/c/zPIKrB/br0sv) | The Netherlands | nationwide | 5·3, 52·1 | 1998 - 2005 | inpatient | ALRI | Virus detection; Antigen detection; Nucleic acid detection | Threshold-based | Week |
| Meningher, 2014 [[126]](https://paperpile.com/c/zPIKrB/LCRZ) | Israel | nationwide | 34·9, 31 | 2007 - 2012 | Unknown | ARI or ILI | Nucleic acid detection | Qualitative | Week |
| Midgley, 2017 [[127]](https://paperpile.com/c/zPIKrB/LX59k) | USA | nationwide | -95·7, 37·1 | 2005 - 2015 | Unknown | Unknown | Virus detection; Antigen detection; Nucleic acid detection | Threshold-based | Week |
| Miller, 2013 [[128]](https://paperpile.com/c/zPIKrB/7hOJ) | USA | Tennessee | -86·6, 35·5 | 2004 - 2008 | outpatient; inpatient | ARI or ILI | Nucleic acid detection | Qualitative | Month |
| Miyama, 2021 [[129]](https://paperpile.com/c/zPIKrB/hD5ZO) | Japan | nationwide | 138·3, 36·2 | 2012 - 2019 | Unknown | Unknown | Antigen detection; Nucleic acid detection | Model-based | Week |
| Mizuta, 2013 [[130]](https://paperpile.com/c/zPIKrB/UQ2i) | Japan | Yamagata prefecture | 140·1, 38·5 | 2004 - 2011 | outpatient | ARI or ILI | Antibody detection; Antigen detection;Virus detection; Nucleic acid detection | Qualitative | Month |
| Mlinaric-Galinovic, 2008[[131]](https://paperpile.com/c/zPIKrB/otzRz) | Croatia | Zagreb County | 16·4, 45·9 | 1994 - 2005 | inpatient | ARI or ILI | Virus detection; Antigen detection | Qualitative | Month |
| Montgomery, 2021 [[132]](https://paperpile.com/c/zPIKrB/3KyN) | USA | Oahu | -158, 21·4 | 2014 - 2018 | outpatient; inpatient | Unknown | Virus detection; Antigen detection; Nucleic acid detection | Qualitative | Month |
| Moore, 2009 [[133]](https://paperpile.com/c/zPIKrB/mAtYr) | Australia | Perth | 115·9, -32 | 1997 - 2005 | outpatient; inpatient | Unknown | Virus detection; Antigen detection | Qualitative | Month |
| Morley, 2018 [[134]](https://paperpile.com/c/zPIKrB/5eVn) | Australia | Gold Coast region of South East Queensland | 153·4, -28 | 2007 - 2016 | outpatient; inpatient | ARI or ILI | Antigen detection; Nucleic acid detection | Qualitative | Month |
| Moura, 2013 [[135]](https://paperpile.com/c/zPIKrB/yqMda) | Brazil | Fortaleza | -38·5, -3·7 | 2004 - 2008 | Unknown | ARI or ILI | Antigen detection | Threshold-based | Week |
| Movva, 2022 [[136]](https://paperpile.com/c/zPIKrB/ddPlR) | USA | nationwide | -95·7, 37·1 | 2015 - 2020 | outpatient; inpatient | Unknown | Unknown | Qualitative | NA |
| Mufson, 1973 [[137]](https://paperpile.com/c/zPIKrB/ACqt) | USA | Chicago | -87·6, 41·9 | 1967 - 1971 | inpatient | ALRI | Virus detection; Antibody detection | Qualitative | Month |
| Mullins, 2003 [[138]](https://paperpile.com/c/zPIKrB/QzfFW) | USA | Midwest | -93·1, 41·9 | 1990 - 2000 | Unknown | Unknown | Antigen detection | Threshold-based | Week |
| Mullins, 2003 [[138]](https://paperpile.com/c/zPIKrB/QzfFW) | USA | nationwide | -95·7, 37·1 | 1990 - 2000 | Unknown | Unknown | Antigen detection | Threshold-based | Week |
| Mullins, 2003 [[138]](https://paperpile.com/c/zPIKrB/QzfFW) | USA | Northeast | -74·2, 43·3 | 1990 - 2000 | Unknown | Unknown | Antigen detection | Threshold-based | Week |
| Mullins, 2003 [[138]](https://paperpile.com/c/zPIKrB/QzfFW) | USA | South | -99, 32 | 1990 - 2000 | Unknown | Unknown | Antigen detection | Threshold-based | Week |
| Mullins, 2003 [[138]](https://paperpile.com/c/zPIKrB/QzfFW) | USA | West | -111, 37·1 | 1990 - 2000 | Unknown | Unknown | Antigen detection | Threshold-based | Week |
| Nenna, 2017 [[4]](https://paperpile.com/c/zPIKrB/JH4bW) | Italy | Rome | 12·5, 41·9 | 2004 - 2014 | inpatient | ALRI | Nucleic acid detection | Model-based | Month |
| Noveroske, 2016 [[139]](https://paperpile.com/c/zPIKrB/JiaW8) | USA | Fairfield, Connecticut state | -73·3, 41·1 | 1997 - 2013 | inpatient | Unknown | Unknown | Model-based | Week |
| Noveroske, 2016 [[139]](https://paperpile.com/c/zPIKrB/JiaW8) | USA | Hartford, Connecticut state | -72·7, 41·8 | 1997 - 2013 | inpatient | Unknown | Unknown | Model-based | Week |
| Noveroske, 2016 [[139]](https://paperpile.com/c/zPIKrB/JiaW8) | USA | Litchfield, Connecticut state | -73·2, 41·7 | 1997 - 2013 | inpatient | Unknown | Unknown | Model-based | Week |
| Noveroske, 2016 [[139]](https://paperpile.com/c/zPIKrB/JiaW8) | USA | Middlesex, Connecticut state | -72·5, 41·5 | 1997 - 2013 | inpatient | Unknown | Unknown | Model-based | Week |
| Noveroske, 2016 [[139]](https://paperpile.com/c/zPIKrB/JiaW8) | USA | New Haven, Connecticut state | -72·9, 41·3 | 1997 - 2013 | inpatient | Unknown | Unknown | Model-based | Week |
| Noveroske, 2016 [[139]](https://paperpile.com/c/zPIKrB/JiaW8) | USA | New London, Connecticut state | -72·1, 41·4 | 1997 - 2013 | inpatient | Unknown | Unknown | Model-based | Week |
| Noveroske, 2016 [[139]](https://paperpile.com/c/zPIKrB/JiaW8) | USA | Tolland, Connecticut state | -72·4, 41·9 | 1997 - 2013 | inpatient | Unknown | Unknown | Model-based | Week |
| Noveroske, 2016 [[139]](https://paperpile.com/c/zPIKrB/JiaW8) | USA | Windham, Connecticut state | -72·2, 41·7 | 1997 - 2013 | inpatient | Unknown | Unknown | Model-based | Week |
| Nyoka, 2017 [[140]](https://paperpile.com/c/zPIKrB/hdTg7) | Kenya | Dadaab | 40·3, 0·1 | 2007 - 2011 | outpatient | ARI or ILI | Nucleic acid detection | Qualitative | Month |
| O'Kelly, 1991 [[141]](https://paperpile.com/c/zPIKrB/o3vf) | Republic of Ireland | Dublin | -6·3, 53·3 | 1987 - 1990 | inpatient | ARI or ILI | Antigen detection | Qualitative | Month |
| Obando-Pacheco, 2018 [[142]](https://paperpile.com/c/zPIKrB/h4sVb) | Argentina | nationwide | -63·6, -38·4 | 2011 - 2017 | Unknown | Unknown | Unknown | Threshold-based | Week |
| Obando-Pacheco, 2018 [[142]](https://paperpile.com/c/zPIKrB/h4sVb) | Australia | nationwide | 133·8, -25·3 | 2009 - 2016 | Unknown | Unknown | Unknown | Threshold-based | Week |
| Obando-Pacheco, 2018 [[142]](https://paperpile.com/c/zPIKrB/h4sVb) | Belgium | nationwide | 4·5, 50·5 | 2004 - 2014 | Unknown | Unknown | Unknown | Threshold-based | Week |
| Obando-Pacheco, 2018 [[142]](https://paperpile.com/c/zPIKrB/h4sVb) | Brazil | nationwide | -51·9, -14·2 | 2009 - 2017 | Unknown | Unknown | Unknown | Threshold-based | Week |
| Obando-Pacheco, 2018 [[142]](https://paperpile.com/c/zPIKrB/h4sVb) | Canada | nationwide | -106·3, 56·1 | 2012 - 2017 | Unknown | Unknown | Unknown | Threshold-based | Week |
| Obando-Pacheco, 2018 [[142]](https://paperpile.com/c/zPIKrB/h4sVb) | Chile | nationwide | -71·5, -35·7 | 2011 - 2017 | Unknown | Unknown | Unknown | Threshold-based | Week |
| Obando-Pacheco, 2018 [[142]](https://paperpile.com/c/zPIKrB/h4sVb) | China | nationwide | 104·2, 35·9 | 2010 - 2015 | Unknown | Unknown | Unknown | Threshold-based | Month |
| Obando-Pacheco, 2018 [[142]](https://paperpile.com/c/zPIKrB/h4sVb) | Finland | nationwide | 25·7, 61·9 | 2010 - 2015 | Unknown | Unknown | Unknown | Threshold-based | Week |
| Obando-Pacheco, 2018 [[142]](https://paperpile.com/c/zPIKrB/h4sVb) | France | nationwide | 2·7, 47·1 | 2011 - 2017 | Unknown | Unknown | Unknown | Threshold-based | Week |
| Obando-Pacheco, 2018 [[142]](https://paperpile.com/c/zPIKrB/h4sVb) | France | nationwide | 2·7, 47·1 | 2011 - 2017 | Unknown | Unknown | Unknown | Threshold-based | Month |
| Obando-Pacheco, 2018 [[142]](https://paperpile.com/c/zPIKrB/h4sVb) | Germany | nationwide | 9·7, 50·9 | 2010 - 2017 | Unknown | Unknown | Unknown | Threshold-based | Week |
| Obando-Pacheco, 2018 [[142]](https://paperpile.com/c/zPIKrB/h4sVb) | Germany | nationwide | 9·7, 50·9 | 2010 - 2017 | Unknown | Unknown | Unknown | Threshold-based | Week |
| Obando-Pacheco, 2018 [[142]](https://paperpile.com/c/zPIKrB/h4sVb) | Greece | nationwide | 21·8, 39·1 | 1999 - 2013 | Unknown | Unknown | Unknown | Threshold-based | Month |
| Obando-Pacheco, 2018 [[142]](https://paperpile.com/c/zPIKrB/h4sVb) | Guatemala | nationwide | -90·2, 15·8 | 2015 - 2017 | Unknown | Unknown | Unknown | Threshold-based | Week |
| Obando-Pacheco, 2018 [[142]](https://paperpile.com/c/zPIKrB/h4sVb) | Israel | nationwide | 34·9, 31 | 2005 - 2017 | Unknown | Unknown | Unknown | Threshold-based | Week |
| Obando-Pacheco, 2018 [[142]](https://paperpile.com/c/zPIKrB/h4sVb) | Italy | nationwide | 12·6, 41·9 | 2000 - 2014 | Unknown | Unknown | Unknown | Threshold-based | Month |
| Obando-Pacheco, 2018 [[142]](https://paperpile.com/c/zPIKrB/h4sVb) | Japan | nationwide | 138·3, 36·2 | 2010 - 2017 | Unknown | Unknown | Unknown | Threshold-based | Week |
| Obando-Pacheco, 2018 [[142]](https://paperpile.com/c/zPIKrB/h4sVb) | Malaysia | nationwide | 102, 4·2 | 1982 - 2008 | Unknown | Unknown | Unknown | Threshold-based | Month |
| Obando-Pacheco, 2018 [[142]](https://paperpile.com/c/zPIKrB/h4sVb) | Mexico | nationwide | -102·6, 23·6 | 2012 - 2015 | Unknown | Unknown | Unknown | Threshold-based | Week |
| Obando-Pacheco, 2018 [[142]](https://paperpile.com/c/zPIKrB/h4sVb) | Mozambique | nationwide | 35·5, -18·7 | 1998 - 2000 | Unknown | Unknown | Unknown | Threshold-based | Month |
| Obando-Pacheco, 2018 [[142]](https://paperpile.com/c/zPIKrB/h4sVb) | New Zealand | nationwide | 174·9, -40·9 | 2010 - 2015 | Unknown | Unknown | Unknown | Threshold-based | Week |
| Obando-Pacheco, 2018 [[142]](https://paperpile.com/c/zPIKrB/h4sVb) | Philippines | nationwide | 121·8, 12·9 | 2010 - 2013 | Unknown | Unknown | Unknown | Threshold-based | Month |
| Obando-Pacheco, 2018 [[142]](https://paperpile.com/c/zPIKrB/h4sVb) | South Africa | nationwide | 22·9, -30·6 | 2009 - 2016 | Unknown | Unknown | Unknown | Threshold-based | Week |
| Obando-Pacheco, 2018 [[142]](https://paperpile.com/c/zPIKrB/h4sVb) | South Korea | nationwide | 127·8, 35·9 | 2008 - 2016 | Unknown | Unknown | Unknown | Threshold-based | Month |
| Obando-Pacheco, 2018 [[142]](https://paperpile.com/c/zPIKrB/h4sVb) | Spain | nationwide | -3·3, 39·7 | 2010 - 2017 | Unknown | Unknown | Unknown | Threshold-based | Week |
| Obando-Pacheco, 2018 [[142]](https://paperpile.com/c/zPIKrB/h4sVb) | Thailand | nationwide | 101, 15·9 | 2005 - 2013 | Unknown | Unknown | Unknown | Threshold-based | Month |
| Obando-Pacheco, 2018 [[142]](https://paperpile.com/c/zPIKrB/h4sVb) | The Netherlands | nationwide | 5·3, 52·1 | 2010 - 2016 | Unknown | Unknown | Unknown | Threshold-based | Week |
| Obando-Pacheco, 2018 [[142]](https://paperpile.com/c/zPIKrB/h4sVb) | United Kingdom | nationwide | -1·6, 52·7 | 2010 - 2016 | Unknown | Unknown | Unknown | Threshold-based | Week |
| Obando-Pacheco, 2018 [[142]](https://paperpile.com/c/zPIKrB/h4sVb) | USA | nationwide | -95·7, 37·1 | 2011 - 2017 | Unknown | Unknown | Unknown | Threshold-based | Week |
| Oliveira-Santos, 2016 [[143]](https://paperpile.com/c/zPIKrB/uLso) | Portugal | Vila Real district | -7·7, 41·3 | 2005 - 2015 | inpatient | ALRI | Antigen detection | Qualitative | Month |
| Oskarsson, 2022 [[144]](https://paperpile.com/c/zPIKrB/zY6Hw) | Iceland | nationwide | -21·1, 64·4 | 2015 - 2020 | outpatient; inpatient | Unknown | Nucleic acid detection | Qualitative | Month |
| Paes, 2013 [[145]](https://paperpile.com/c/zPIKrB/ChHdE) | Canada | Hamilton | -79·9, 43·3 | 2002 - 2011 | outpatient; inpatient | Unknown | Antigen detection; Nucleic acid detection | Threshold-based | Week |
| Paiva, 2012 [[146]](https://paperpile.com/c/zPIKrB/PfEIA) | Brazil | Sao Paulo | -46·6, -23·6 | 1996 - 2010 | inpatient | ARI or ILI; ALRI | Antigen detection | Model-based | Month |
| Paiva, 2012 [[146]](https://paperpile.com/c/zPIKrB/PfEIA) | Brazil | Sao Paulo | -46·6, -23·6 | 1996 - 2010 | inpatient | ARI or ILI; ALRI | Antigen detection | Qualitative | Month |
| Panozzo, 2010 [[147]](https://paperpile.com/c/zPIKrB/6twje) | USA | Atlanta | -84·4, 33·7 | 2002 - 2007 | Unknown | Unknown | Virus detection; Antigen detection; Nucleic acid detection | Threshold-based | Week |
| Panozzo, 2010 [[147]](https://paperpile.com/c/zPIKrB/6twje) | USA | Birmingham | -86·8, 33·5 | 2002 - 2007 | Unknown | Unknown | Virus detection; Antigen detection; Nucleic acid detection | Threshold-based | Week |
| Panozzo, 2010 [[147]](https://paperpile.com/c/zPIKrB/6twje) | USA | Cleveland | -81·7, 41·5 | 2002 - 2007 | Unknown | Unknown | Virus detection; Antigen detection; Nucleic acid detection | Threshold-based | Week |
| Panozzo, 2010 [[147]](https://paperpile.com/c/zPIKrB/6twje) | USA | Columbia | -81, 34 | 2002 - 2007 | Unknown | Unknown | Virus detection; Antigen detection; Nucleic acid detection | Threshold-based | Week |
| Panozzo, 2010 [[147]](https://paperpile.com/c/zPIKrB/6twje) | USA | Corpus Christi | -97·4, 27·8 | 2002 - 2007 | Unknown | Unknown | Virus detection; Antigen detection; Nucleic acid detection | Threshold-based | Week |
| Panozzo, 2010 [[147]](https://paperpile.com/c/zPIKrB/6twje) | USA | Honolulu | -157·9, 21·3 | 2002 - 2007 | Unknown | Unknown | Virus detection; Antigen detection; Nucleic acid detection | Threshold-based | Week |
| Panozzo, 2010 [[147]](https://paperpile.com/c/zPIKrB/6twje) | USA | Indianapolis | -86·2, 39·8 | 2002 - 2007 | Unknown | Unknown | Virus detection; Antigen detection; Nucleic acid detection | Threshold-based | Week |
| Panozzo, 2010 [[147]](https://paperpile.com/c/zPIKrB/6twje) | USA | Long Beach | -118·2, 33·8 | 2002 - 2007 | Unknown | Unknown | Virus detection; Antigen detection; Nucleic acid detection | Threshold-based | Week |
| Panozzo, 2010 [[147]](https://paperpile.com/c/zPIKrB/6twje) | USA | Los Angeles | -118·2, 34·1 | 2002 - 2007 | Unknown | Unknown | Virus detection; Antigen detection; Nucleic acid detection | Threshold-based | Week |
| Panozzo, 2010 [[147]](https://paperpile.com/c/zPIKrB/6twje) | USA | Nashville | -86·8, 36·2 | 2002 - 2007 | Unknown | Unknown | Virus detection; Antigen detection; Nucleic acid detection | Threshold-based | Week |
| Panozzo, 2010 [[147]](https://paperpile.com/c/zPIKrB/6twje) | USA | New Orleans | -90·1, 30 | 2002 - 2007 | Unknown | Unknown | Virus detection; Antigen detection; Nucleic acid detection | Threshold-based | Week |
| Panozzo, 2010 [[147]](https://paperpile.com/c/zPIKrB/6twje) | USA | Oklahoma City | -97·5, 35·5 | 2002 - 2007 | Unknown | Unknown | Virus detection; Antigen detection; Nucleic acid detection | Threshold-based | Week |
| Panozzo, 2010 [[147]](https://paperpile.com/c/zPIKrB/6twje) | USA | Richmond | -77·4, 37·5 | 2002 - 2007 | Unknown | Unknown | Virus detection; Antigen detection; Nucleic acid detection | Threshold-based | Week |
| Panozzo, 2010 [[147]](https://paperpile.com/c/zPIKrB/6twje) | USA | San Antonio | -98·5, 29·4 | 2002 - 2007 | Unknown | Unknown | Virus detection; Antigen detection; Nucleic acid detection | Threshold-based | Week |
| Panozzo, 2010 [[147]](https://paperpile.com/c/zPIKrB/6twje) | USA | San Diego | -117·2, 32·7 | 2002 - 2007 | Unknown | Unknown | Virus detection; Antigen detection; Nucleic acid detection | Threshold-based | Week |
| Panozzo, 2010 [[147]](https://paperpile.com/c/zPIKrB/6twje) | USA | Seattle | -122·3, 47·6 | 2002 - 2007 | Unknown | Unknown | Virus detection; Antigen detection; Nucleic acid detection | Threshold-based | Week |
| Panozzo, 2010 [[147]](https://paperpile.com/c/zPIKrB/6twje) | USA | Sioux Falls | -96·7, 43·5 | 2002 - 2007 | Unknown | Unknown | Virus detection; Antigen detection; Nucleic acid detection | Threshold-based | Week |
| Panozzo, 2010 [[147]](https://paperpile.com/c/zPIKrB/6twje) | USA | St Louis | -90·2, 38·6 | 2002 - 2007 | Unknown | Unknown | Virus detection; Antigen detection; Nucleic acid detection | Threshold-based | Week |
| Panozzo, 2010 [[147]](https://paperpile.com/c/zPIKrB/6twje) | USA | St Louis | -90·2, 38·6 | 2002 - 2007 | Unknown | Unknown | Virus detection; Antigen detection; Nucleic acid detection | Threshold-based | Week |
| Paynter, 2015 [[148]](https://paperpile.com/c/zPIKrB/2gJR) | Australia | Cairns | 145·8, -16·9 | 1999 - 2012 | inpatient | Unknown | Unknown | Qualitative | Month |
| Paynter, 2015 [[148]](https://paperpile.com/c/zPIKrB/2gJR) | Australia | Townsville | 146·8, -19·3 | 1999 - 2012 | inpatient | Unknown | Unknown | Qualitative | Month |
| Pellegrinelli, 2022 [[149]](https://paperpile.com/c/zPIKrB/Ox5th) | Italy | nationwide | 12·6, 41·9 | 2014 - 2019 | outpatient | ARI or ILI | Unknown | Threshold-based | Week |
| Peterson, 2016 [[150]](https://paperpile.com/c/zPIKrB/2IZU) | Malawi | Blantyre | 35, -15·8 | 2011 - 2014 | Unknown | SARI | Nucleic acid detection | Qualitative | Month |
| Pierangeli, 2014 [[151]](https://paperpile.com/c/zPIKrB/5UR0) | Italy | Ancona | 13·5, 43·6 | 2010 - 2013 | inpatient | SARI | Nucleic acid detection | Qualitative | Month |
| Pierangeli, 2014 [[151]](https://paperpile.com/c/zPIKrB/5UR0) | Italy | Rome | 12·5, 41·9 | 2010 - 2013 | inpatient | SARI | Nucleic acid detection | Qualitative | Month |
| Price, 2019 [[152]](https://paperpile.com/c/zPIKrB/b012) | United Kingdom | Edinburgh | -3·2, 56 | 2009 - 2015 | Unknown | Unknown | Nucleic acid detection | Qualitative | Month |
| Ramaekers, 2017 [[153]](https://paperpile.com/c/zPIKrB/eCEh) | Belgium | Leuven | 4·7, 50·9 | 2011 - 2016 | Unknown | ARI or ILI | Nucleic acid detection | Qualitative | Week |
| Reeves, 2016 [[154]](https://paperpile.com/c/zPIKrB/3NLPe) | United Kingdom | England | -1·2, 52·4 | 2011 - 2014 | outpatient; inpatient | Unknown | Nucleic acid detection | Threshold-based | Week |
| Reiche, 2009 [[155]](https://paperpile.com/c/zPIKrB/lp0rv) | Germany | nationwide | 9·7, 50·9 | 1998 - 2007 | outpatient; inpatient | ARI or ILI | Nucleic acid detection | Threshold-based | Week |
| Renko, 2019 [[156]](https://paperpile.com/c/zPIKrB/pBuC) | Finland | nationwide | 25·7, 61·9 | 1995 - 2018 | Unknown | Unknown | Nucleic acid detection | Qualitative | Month |
| Reyes, 1997 [[157]](https://paperpile.com/c/zPIKrB/uo77Z) | Sweden | Stockholm | 18·1, 59·3 | 1984 - 1993 | inpatient | Unknown | Antigen detection | Threshold-based | Week |
| Richter, 2016 [[158]](https://paperpile.com/c/zPIKrB/NkWB) | Cyprus | Nicosia | 33·4, 35·2 | 2010 - 2013 | inpatient | ARI or ILI | Nucleic acid detection | Qualitative | Month |
| Rose, 2018 [[159]](https://paperpile.com/c/zPIKrB/l3Uth) | USA | Atlanta | -84·4, 33·7 | 2014 - 2017 | Unknown | Unknown | Virus detection; Antigen detection; Nucleic acid detection | Threshold-based | Week |
| Rose, 2018 [[159]](https://paperpile.com/c/zPIKrB/l3Uth) | USA | Boston | -71·1, 42·4 | 2014 - 2017 | Unknown | Unknown | Virus detection; Antigen detection; Nucleic acid detection | Threshold-based | Week |
| Rose, 2018 [[159]](https://paperpile.com/c/zPIKrB/l3Uth) | USA | Chicago | -87·6, 41·9 | 2014 - 2017 | Unknown | Unknown | Virus detection; Antigen detection; Nucleic acid detection | Threshold-based | Week |
| Rose, 2018 [[159]](https://paperpile.com/c/zPIKrB/l3Uth) | USA | Dellas | -96·8, 32·8 | 2014 - 2017 | Unknown | Unknown | Virus detection; Antigen detection; Nucleic acid detection | Threshold-based | Week |
| Rose, 2018 [[159]](https://paperpile.com/c/zPIKrB/l3Uth) | USA | Denver | -105, 39·7 | 2014 - 2017 | Unknown | Unknown | Virus detection; Antigen detection; Nucleic acid detection | Threshold-based | Week |
| Rose, 2018 [[159]](https://paperpile.com/c/zPIKrB/l3Uth) | USA | Kansas City | -94·6, 39·1 | 2014 - 2017 | Unknown | Unknown | Virus detection; Antigen detection; Nucleic acid detection | Threshold-based | Week |
| Rose, 2018 [[159]](https://paperpile.com/c/zPIKrB/l3Uth) | USA | nationwide | -95·7, 37·1 | 2014 - 2017 | Unknown | Unknown | Virus detection; Antigen detection; Nucleic acid detection | Threshold-based | Week |
| Rose, 2018 [[159]](https://paperpile.com/c/zPIKrB/l3Uth) | USA | nationwide | -95·7, 37·1 | 2014 - 2017 | Unknown | Unknown | Virus detection; Antigen detection; Nucleic acid detection | Threshold-based | Week |
| Rose, 2018 [[159]](https://paperpile.com/c/zPIKrB/l3Uth) | USA | New York | -74, 40·7 | 2014 - 2017 | Unknown | Unknown | Virus detection; Antigen detection; Nucleic acid detection | Threshold-based | Week |
| Rose, 2018 [[159]](https://paperpile.com/c/zPIKrB/l3Uth) | USA | Philadelphia | -75·2, 40 | 2014 - 2017 | Unknown | Unknown | Virus detection; Antigen detection; Nucleic acid detection | Threshold-based | Week |
| Rose, 2018 [[159]](https://paperpile.com/c/zPIKrB/l3Uth) | USA | San Francisco | -122·4, 37·8 | 2014 - 2017 | Unknown | Unknown | Virus detection; Antigen detection; Nucleic acid detection | Threshold-based | Week |
| Rose, 2018 [[159]](https://paperpile.com/c/zPIKrB/l3Uth) | USA | Seattle | -122·3, 47·6 | 2014 - 2017 | Unknown | Unknown | Virus detection; Antigen detection; Nucleic acid detection | Threshold-based | Week |
| Rose, 2020 [[160]](https://paperpile.com/c/zPIKrB/i2Xbo) | Kenya | Kilifi | 39·9, -3·6 | 2006 - 2018 | outpatient; inpatient | ARI or ILI; SARI; ALRI | Nucleic acid detection | Threshold-based | Week |
| Rose, 2020 [[160]](https://paperpile.com/c/zPIKrB/i2Xbo) | Kenya | Nairobi | 36·8, -1·3 | 2006 - 2016 | outpatient; inpatient | ARI or ILI; SARI; ALRI | Nucleic acid detection | Threshold-based | Week |
| Rose, 2020 [[160]](https://paperpile.com/c/zPIKrB/i2Xbo) | Kenya | Siaya | 34·3, 0·1 | 2006 - 2018 | outpatient; inpatient | ARI or ILI; SARI; ALRI | Nucleic acid detection | Threshold-based | Week |
| Rowlinson, 2017 [[161]](https://paperpile.com/c/zPIKrB/99ho) | Egypt | Damanhour | 30·5, 31 | 2009 - 2013 | inpatient | SARI | Nucleic acid detection | Qualitative | Month |
| Rzad, 2022 [[162]](https://paperpile.com/c/zPIKrB/R9I6x) | Poland | nationwide | 19·3, 51·7 | 2010 - 2020 | inpatient | Unknown | Unknown | Qualitative | Month |
| Sato, 2005 [[163]](https://paperpile.com/c/zPIKrB/yDbN) | Japan | Niigata city | 139, 37·9 | 2001 - 2004 | outpatient | ALRI | Nucleic acid detection | Qualitative | Month |
| Shobugawa, 2017 [[164]](https://paperpile.com/c/zPIKrB/dlg8O) | Japan | nationwide | 138·3, 36·2 | 2007 - 2014 | Unknown | Unknown | Unknown | Qualitative | Month |
| Singleton, 2007 [[165]](https://paperpile.com/c/zPIKrB/VnTAx) | USA | YK Delta | -164·2, 62·9 | 1996 - 2004 | inpatient | ALRI | Virus detection; Antigen detection | Threshold-based | Month |
| Sirimi, 2016 [[166]](https://paperpile.com/c/zPIKrB/65sk) | Greece | Athens | 23·7, 37·7 | 2002 - 2013 | inpatient | SARI | Antigen detection | Qualitative | Month |
| Sitthikarnkha, 2022 [[167]](https://paperpile.com/c/zPIKrB/snhh4) | Thailand | nationwide | 101, 15·9 | 2015 - 2020 | outpatient; inpatient | ALRI | Unknown | Qualitative | Month |
| Staadegaard, 2021 [[2]](https://paperpile.com/c/zPIKrB/aIPfL) | Brazil | Midwest | -51·9, -14·2 | 2016 - 2018 | inpatient | Unknown | Unknown | Coverage-based | Week |
| Staadegaard, 2021 [[2]](https://paperpile.com/c/zPIKrB/aIPfL) | Brazil | nationwide | -51·9, -14·2 | 2014 - 2018 | inpatient | Unknown | Unknown | Coverage-based | Week |
| Staadegaard, 2021 [[2]](https://paperpile.com/c/zPIKrB/aIPfL) | Brazil | Northeast | -42·6, -10·1 | 2014 - 2018 | inpatient | Unknown | Unknown | Coverage-based | Week |
| Staadegaard, 2021 [[2]](https://paperpile.com/c/zPIKrB/aIPfL) | Brazil | South | -49·3, -25·4 | 2014 - 2018 | inpatient | Unknown | Unknown | Coverage-based | Week |
| Staadegaard, 2021 [[2]](https://paperpile.com/c/zPIKrB/aIPfL) | Brazil | Southeast | -46·2, -20·3 | 2014 - 2018 | inpatient | Unknown | Unknown | Coverage-based | Week |
| Staadegaard, 2021 [[2]](https://paperpile.com/c/zPIKrB/aIPfL) | Chile | nationwide | -71·5, -35·7 | 2012 - 2018 | inpatient | SARI | Nucleic acid detection; Antigen detection | Coverage-based | Week |
| Staadegaard, 2021 [[2]](https://paperpile.com/c/zPIKrB/aIPfL) | Czech Republic | nationwide | 15·5, 49·8 | 2014 - 2018 | inpatient | Unknown | Virus detection; Antigen detection; Nucleic acid detection | Coverage-based | Week |
| Staadegaard, 2021 [[2]](https://paperpile.com/c/zPIKrB/aIPfL) | Ecuador | nationwide | -78·2, -1·8 | 2012 - 2018 | inpatient | SARI | Antigen detection | Coverage-based | Week |
| Staadegaard, 2021 [[2]](https://paperpile.com/c/zPIKrB/aIPfL) | New Zealand | nationwide | 174·9, -40·9 | 2012 - 2018 | outpatient | ARI or ILI | Nucleic acid detection | Coverage-based | Week |
| Staadegaard, 2021 [[2]](https://paperpile.com/c/zPIKrB/aIPfL) | Portugal | nationwide | -9·2, 39·7 | 2013 - 2018 | outpatient | ARI or ILI | Antigen detection; Nucleic acid detection | Coverage-based | Week |
| Staadegaard, 2021 [[2]](https://paperpile.com/c/zPIKrB/aIPfL) | Singapore | nationwide | 103·8, 1·4 | 2011 - 2018 | inpatient | SARI | Antigen detection; Nucleic acid detection | Coverage-based | Week |
| Staadegaard, 2021 [[2]](https://paperpile.com/c/zPIKrB/aIPfL) | South Africa | nationwide | 22·9, -30·6 | 2010 - 2018 | outpatient | ARI or ILI | Nucleic acid detection | Coverage-based | Week |
| Staadegaard, 2021 [[2]](https://paperpile.com/c/zPIKrB/aIPfL) | Spain | nationwide | -3·3, 39·7 | 2006 - 2018 | inpatient | Unknown | Unknown | Coverage-based | Week |
| Staadegaard, 2021 [[2]](https://paperpile.com/c/zPIKrB/aIPfL) | The Netherlands | nationwide | 5·3, 52·1 | 2000 - 2018 | inpatient | Unknown | Nucleic acid detection | Coverage-based | Week |
| Staadegaard, 2021 [[2]](https://paperpile.com/c/zPIKrB/aIPfL) | USA | HHS1 | 71·1, 42·4 | 2009 - 2018 | Unknown | Unknown | Virus detection; Antigen detection; Nucleic acid detection | Coverage-based | Week |
| Staadegaard, 2021 [[2]](https://paperpile.com/c/zPIKrB/aIPfL) | USA | HHS10 | 122·3, 47·6 | 2008 - 2018 | Unknown | Unknown | Virus detection; Antigen detection; Nucleic acid detection | Coverage-based | Week |
| Staadegaard, 2021 [[2]](https://paperpile.com/c/zPIKrB/aIPfL) | USA | HHS2 | 74, 40·7 | 2008 - 2018 | Unknown | Unknown | Virus detection; Antigen detection; Nucleic acid detection | Coverage-based | Week |
| Staadegaard, 2021 [[2]](https://paperpile.com/c/zPIKrB/aIPfL) | USA | HHS3 | 75·2, 40 | 2007 - 2018 | Unknown | Unknown | Virus detection; Antigen detection; Nucleic acid detection | Coverage-based | Week |
| Staadegaard, 2021 [[2]](https://paperpile.com/c/zPIKrB/aIPfL) | USA | HHS4 | 84·4, 33·8 | 2009 - 2018 | Unknown | Unknown | Virus detection; Antigen detection; Nucleic acid detection | Coverage-based | Week |
| Staadegaard, 2021 [[2]](https://paperpile.com/c/zPIKrB/aIPfL) | USA | HHS5 | 87·6, 41·9 | 2006 - 2018 | Unknown | Unknown | Virus detection; Antigen detection; Nucleic acid detection | Coverage-based | Week |
| Staadegaard, 2021 [[2]](https://paperpile.com/c/zPIKrB/aIPfL) | USA | HHS6 | 96·8, 32·8 | 2008 - 2018 | Unknown | Unknown | Virus detection; Antigen detection; Nucleic acid detection | Coverage-based | Week |
| Staadegaard, 2021 [[2]](https://paperpile.com/c/zPIKrB/aIPfL) | USA | HHS7 | 94·6, 39·1 | 2008 - 2018 | Unknown | Unknown | Virus detection; Antigen detection; Nucleic acid detection | Coverage-based | Week |
| Staadegaard, 2021 [[2]](https://paperpile.com/c/zPIKrB/aIPfL) | USA | HHS8 | 105, 39·7 | 2008 - 2018 | Unknown | Unknown | Virus detection; Antigen detection; Nucleic acid detection | Coverage-based | Week |
| Staadegaard, 2021 [[2]](https://paperpile.com/c/zPIKrB/aIPfL) | USA | HHS9 | 122·4, 37·8 | 2009 - 2018 | Unknown | Unknown | Virus detection; Antigen detection; Nucleic acid detection | Coverage-based | Week |
| Staadegaard, 2021 [[2]](https://paperpile.com/c/zPIKrB/aIPfL) | USA | nationwide | -95·7, 37·1 | 2006 - 2018 | Unknown | Unknown | Virus detection; Antigen detection; Nucleic acid detection | Coverage-based | Week |
| Stockman, 2013 [[168]](https://paperpile.com/c/zPIKrB/9Yq61) | Bangladesh | Dhaka | 90·4, 23·8 | 2004 - 2008 | outpatient | ARI or ILI | Nucleic acid detection | Qualitative | Month |
| Straliotto, 2001 [[169]](https://paperpile.com/c/zPIKrB/KWRS) | Brazil | Porto Alegre | -51·1, -31 | 1990 - 1998 | outpatient; inpatient | ARI or ILI; ALRI | Antigen detection | Qualitative | Month |
| Straliotto, 2002 [[170]](https://paperpile.com/c/zPIKrB/OHWs) | Brazil | Porto Alegre | -51·1, -31 | 1990 - 1992 | outpatient; inpatient | ARI or ILI;ALRI | Antigen detection | Qualitative | Month |
| Sundell, 2016 [[171]](https://paperpile.com/c/zPIKrB/f3Cy) | Sweden | Gothenburg | 12, 57·7 | 2010 - 2013 | outpatient; inpatient | ARI or ILI | Nucleic acid detection | Qualitative | Month |
| Sutmoller, 1995 [[172]](https://paperpile.com/c/zPIKrB/f9vki) | Brazil | Rio de Janeiro | -43·2, -22·9 | 1987 - 1989 | outpatient; inpatient | ARI or ILI | Antigen detection | Qualitative | Month |
| Tabatabai, 2022 [[173]](https://paperpile.com/c/zPIKrB/A1UTM) | Germany | Heidelberg | 8·7, 49·4 | 2014 - 2017 | inpatient | ARI or ILI | Nucleic acid detection | Qualitative | Month |
| Tan, 2021 [[174]](https://paperpile.com/c/zPIKrB/vTj3) | Singapore | nationwide | 103·8, 1·4 | 2011 - 2016 | inpatient | ARI or ILI | Antigen detection | Qualitative | Month |
| Tang, 2010 [[175]](https://paperpile.com/c/zPIKrB/wB8zW) | Hong Kong | nationwide | 114·2, 22·3 | 2000 - 2007 | inpatient | ARI or ILI | Antigen detection | Qualitative | Month |
| Terletskaia-Ladwig, 2005 [[176]](https://paperpile.com/c/zPIKrB/KGT1J) | Germany | Stuttgart | 9·2, 48·8 | 1996 - 2004 | outpatient; inpatient | Clinical judgment | Antigen detection; Nucleic acid detection | Threshold-based | Week |
| Thomas, 1994 [[177]](https://paperpile.com/c/zPIKrB/vvvR) | Canada | British Columbia | -127·6, 53·7 | 1987 - 1992 | inpatient | ARI or ILI | Antigen detection | Qualitative | Month |
| Thongpan, 2020 [[178]](https://paperpile.com/c/zPIKrB/tlHj) | Thailand | nationwide | 101, 15·9 | 2012 - 2018 | Unknown | ARI or ILI | Nucleic acid detection | Qualitative | Month |
| Thwaites, 2020 [[179]](https://paperpile.com/c/zPIKrB/DTr5) | United Kingdom | Scoland | -4·2, 56·5 | 2000 - 2011 | inpatient | Unknown | Unknown | Qualitative | Month |
| Tsolia, 2003 [[180]](https://paperpile.com/c/zPIKrB/TMXA) | Greece | Athens | 23·7, 37·7 | 1997 - 2000 | inpatient | ALRI | Antigen detection | Qualitative | Month |
| Turner, 2012 [[181]](https://paperpile.com/c/zPIKrB/Y1v0) | Thailand | Maela camp | 98·4, 17·2 | 2007 - 2010 | outpatient | ALRI | Nucleic acid detection | Qualitative | Month |
| Ucakar, 2013 [[182]](https://paperpile.com/c/zPIKrB/dkxqZ) | Slovenia | nationwide | 14·9, 46·2 | 2006 - 2011 | outpatient; inpatient | ARI or ILI | Antigen detection; Nucleic acid detection | Threshold-based | Week |
| Valley-Omar, 2022* [[183]](https://paperpile.com/c/zPIKrB/1Q5Yl) | South Africa | KwaZulu-Natal province | 30·9, -28·5 | 2012 - 2015 | inpatient | SARI | Nucleic acid detection | Qualitative | Month |
| Valley-Omar, 2022* [[183]](https://paperpile.com/c/zPIKrB/1Q5Yl) | South Africa | North West Province | 25·3, -26·7 | 2012 - 2015 | inpatient | SARI | Nucleic acid detection | Qualitative | Month |
| van der Sande, 2004 [[184]](https://paperpile.com/c/zPIKrB/YMZz) | Gambia | Banjul | -16·6, 13·5 | 1993 - 2002 | inpatient | SARI | Antigen detection | Qualitative | Month |
| van Summeren, 2021 [[185]](https://paperpile.com/c/zPIKrB/HoDc) | France | France | 2·7, 47·1 | 2016 - 2021 | outpatient; inpatient | ARI or ILI; Clinical judgment | Virus detection; Antibody detection; Antigen detection; Nucleic acid detection | Qualitative | Week |
| van Summeren, 2021 [[185]](https://paperpile.com/c/zPIKrB/HoDc) | Iceland | Iceland | -21·1, 64·4 | 2016 - 2021 | outpatient; inpatient | ARI or ILI; Clinical judgment | Virus detection; Antibody detection; Antigen detection; Nucleic acid detection | Qualitative | Week |
| van Summeren, 2021 [[185]](https://paperpile.com/c/zPIKrB/HoDc) | The Netherlands | The Netherlands | 5·3, 52·1 | 2016 - 2021 | outpatient; inpatient | ARI or ILI; Clinical judgment | Virus detection; Antibody detection; Antigen detection; Nucleic acid detection | Qualitative | Week |
| Vandini, 2013 [[186]](https://paperpile.com/c/zPIKrB/4NIp) | Italy | Bologna | 11·3, 44·5 | 2007 - 2010 | outpatient | ARI or ILI | Antigen detection | Qualitative | Week |
| Verani, 2013 [[187]](https://paperpile.com/c/zPIKrB/el7n) | Guatemala | Santa Rosa, Guateala and Quetzaltenango | -90·4, 14·2 | 2007 - 2011 | inpatient | ARI or ILI | Nucleic acid detection | Qualitative | Month |
| Viegas, 2004 [[188]](https://paperpile.com/c/zPIKrB/OHDD) | Argentina | Buenos Aires city and Greater Buenos Aires | -58·4, -34·6 | 1998 - 2002 | inpatient | ALRI | Antigen detection | Qualitative | Month |
| Viguria, 2018 [[189]](https://paperpile.com/c/zPIKrB/vwxwK) | Spain | Navarra | -1·7, 42·7 | 2010 - 2015 | inpatient | Unknown | Antigen detection; Nucleic acid detection | Threshold-based | Week |
| Vila, 2022 [[190]](https://paperpile.com/c/zPIKrB/QTdsL) | Spain | Barcelona | 2·2, 41·4 | 2012 - 2020 | outpatient; inpatient | ALRI | Antigen detection; Nucleic acid detection | Qualitative | Month |
| Vos, 2019 [[191]](https://paperpile.com/c/zPIKrB/6BC5G) | The Netherlands | nationwide | 5·3, 52·1 | 2005 - 2017 | outpatient; inpatient | ARI or ILI | Virus detection; Antibody detection; Antigen detection; Nucleic acid detection | Coverage-based | Week |
| Vos, 2019 [[191]](https://paperpile.com/c/zPIKrB/6BC5G) | The Netherlands | nationwide | 5·3, 52·1 | 2005 - 2017 | outpatient; inpatient | ARI or ILI | Virus detection; Antibody detection; Antigen detection; Nucleic acid detection | Threshold-based | Week |
| Vos, 2019 [[191]](https://paperpile.com/c/zPIKrB/6BC5G) | The Netherlands | nationwide | 5·3, 52·1 | 2005 - 2017 | outpatient; inpatient | ARI or ILI | Virus detection; Antibody detection; Antigen detection; Nucleic acid detection | Coverage-based | Week |
| Vos, 2019 [[191]](https://paperpile.com/c/zPIKrB/6BC5G) | The Netherlands | nationwide | 5·3, 52·1 | 2005 - 2017 | outpatient; inpatient | ARI or ILI | Virus detection; Antibody detection; Antigen detection; Nucleic acid detection | Threshold-based | Week |
| Wagatsuma, 2021 [[192]](https://paperpile.com/c/zPIKrB/Yt6m7) | Japan | nationwide | 138·3, 36·2 | 2014 - 2017 | Unknown | Unknown | Unknown | Coverage-based | Month |
| Wahab, 2001 [[193]](https://paperpile.com/c/zPIKrB/g9zZ) | Qatar | Doha | 51·5, 25·3 | 1996 - 1998 | inpatient | Unknown | Antigen detection | Qualitative | Month |
| Wang, 2022 [[194]](https://paperpile.com/c/zPIKrB/an6hH) | USA | nationwide | -95·7, 37·1 | 2010 - 2022 | Unknown | Unknown | Unknown | Qualitative | Month |
| Weber, 1998 [[195]](https://paperpile.com/c/zPIKrB/4DDq) | Gambia | Western Region | -16·6, 13·2 | 1993 - 1996 | inpatient | ALRI | Antigen detection | Qualitative | Month |
| Weigl, 2000 [[196]](https://paperpile.com/c/zPIKrB/n9ew) | Germany | Kiel | 10·1, 54·3 | 1995 - 1999 | inpatient | ARI or ILI | Nucleic acid detection | Qualitative | Month |
| Weigl, 2002 [[197]](https://paperpile.com/c/zPIKrB/7fCuW) | Germany | Kiel | 10·1, 54·3 | 1994 - 2001 | inpatient | SARI | Antigen detection; Nucleic acid detection | Qualitative | Month |
| Weigl, 2002 [[197]](https://paperpile.com/c/zPIKrB/7fCuW) | Germany | Kiel | 10·1, 54·3 | 1994 - 2001 | inpatient | SARI | Antigen detection; Nucleic acid detection | Qualitative | Month |
| Weissenbacher, 1990 [[198]](https://paperpile.com/c/zPIKrB/xcs0) | Argentina | Buenos Aires | -58·4, -34·6 | 1984 - 1987 | outpatient; inpatient | ALRI | Antigen detection | Qualitative | Month |
| Wilfret, 2008 [[199]](https://paperpile.com/c/zPIKrB/pdOs) | USA | North Carolina | -79, 35·8 | 2003 - 2006 | outpatient; inpatient | Unknown | Antigen detection | Threshold-based | Month |
| Winter, 1996 [[200]](https://paperpile.com/c/zPIKrB/rzNg) | United Kingdom | Edinburgh | -3·2, 56 | 1985 - 1994 | inpatient | Unknown | Virus detection | Qualitative | Month |
| Wrotek, 2020 [[201]](https://paperpile.com/c/zPIKrB/u8hZN) | Poland | nationwide | 19·3, 51·7 | 2010 - 2017 | inpatient | Unknown | Unknown | Threshold-based | Week |
| Wrotek, 2020 [[201]](https://paperpile.com/c/zPIKrB/u8hZN) | Poland | Warsaw | 21, 52·2 | 2010 - 2017 | inpatient | Unknown | Unknown | Threshold-based | Month |
| Yamagami, 2019 [[202]](https://paperpile.com/c/zPIKrB/hfrAP) | Japan | Aichi prefecture | 137·3, 35 | 2012 - 2018 | Unknown | Unknown | Antigen detection; Nucleic acid detection | Coverage-based | Week |
| Yamagami, 2019 [[202]](https://paperpile.com/c/zPIKrB/hfrAP) | Japan | Akita prefecture | 140·3, 40·1 | 2012 - 2018 | Unknown | Unknown | Antigen detection; Nucleic acid detection | Coverage-based | Week |
| Yamagami, 2019 [[202]](https://paperpile.com/c/zPIKrB/hfrAP) | Japan | Aomori prefecture | 140·9, 40·8 | 2012 - 2018 | Unknown | Unknown | Antigen detection; Nucleic acid detection | Coverage-based | Week |
| Yamagami, 2019 [[202]](https://paperpile.com/c/zPIKrB/hfrAP) | Japan | Chiba prefecture | 140·2, 35·3 | 2012 - 2018 | Unknown | Unknown | Antigen detection; Nucleic acid detection | Coverage-based | Week |
| Yamagami, 2019 [[202]](https://paperpile.com/c/zPIKrB/hfrAP) | Japan | Ehime prefecture | 132·8, 33·6 | 2012 - 2018 | Unknown | Unknown | Antigen detection; Nucleic acid detection | Coverage-based | Week |
| Yamagami, 2019 [[202]](https://paperpile.com/c/zPIKrB/hfrAP) | Japan | Fukui prefecture | 136·2, 35·9 | 2012 - 2018 | Unknown | Unknown | Antigen detection; Nucleic acid detection | Coverage-based | Week |
| Yamagami, 2019 [[202]](https://paperpile.com/c/zPIKrB/hfrAP) | Japan | Fukuoka prefecture | 130·7, 33·6 | 2012 - 2018 | Unknown | Unknown | Antigen detection; Nucleic acid detection | Coverage-based | Week |
| Yamagami, 2019 [[202]](https://paperpile.com/c/zPIKrB/hfrAP) | Japan | Fukushima prefecture | 140·2, 37·4 | 2012 - 2018 | Unknown | Unknown | Antigen detection; Nucleic acid detection | Coverage-based | Week |
| Yamagami, 2019 [[202]](https://paperpile.com/c/zPIKrB/hfrAP) | Japan | Gifu prefecture | 137, 35·7 | 2012 - 2018 | Unknown | Unknown | Antigen detection; Nucleic acid detection | Coverage-based | Week |
| Yamagami, 2019 [[202]](https://paperpile.com/c/zPIKrB/hfrAP) | Japan | Gunma prefecture | 138·9, 36·6 | 2012 - 2018 | Unknown | Unknown | Antigen detection; Nucleic acid detection | Coverage-based | Week |
| Yamagami, 2019 [[202]](https://paperpile.com/c/zPIKrB/hfrAP) | Japan | Hiroshima prefecture | 133, 34·9 | 2012 - 2018 | Unknown | Unknown | Antigen detection; Nucleic acid detection | Coverage-based | Week |
| Yamagami, 2019 [[202]](https://paperpile.com/c/zPIKrB/hfrAP) | Japan | Hokkaido prefecture | 142·9, 43·2 | 2012 - 2018 | Unknown | Unknown | Antigen detection; Nucleic acid detection | Coverage-based | Week |
| Yamagami, 2019 [[202]](https://paperpile.com/c/zPIKrB/hfrAP) | Japan | Hyogo prefecture | 134·5, 34·9 | 2012 - 2018 | Unknown | Unknown | Antigen detection; Nucleic acid detection | Coverage-based | Week |
| Yamagami, 2019 [[202]](https://paperpile.com/c/zPIKrB/hfrAP) | Japan | Ibaraki prefecture | 140·2, 36·2 | 2012 - 2018 | Unknown | Unknown | Antigen detection; Nucleic acid detection | Coverage-based | Week |
| Yamagami, 2019 [[202]](https://paperpile.com/c/zPIKrB/hfrAP) | Japan | Ishikawa prefecture | 136·5, 36·3 | 2012 - 2018 | Unknown | Unknown | Antigen detection; Nucleic acid detection | Coverage-based | Week |
| Yamagami, 2019 [[202]](https://paperpile.com/c/zPIKrB/hfrAP) | Japan | Iwate prefecture | 141·3, 39·6 | 2012 - 2018 | Unknown | Unknown | Antigen detection; Nucleic acid detection | Coverage-based | Week |
| Yamagami, 2019 [[202]](https://paperpile.com/c/zPIKrB/hfrAP) | Japan | Kagawa prefecture | 134, 34·2 | 2012 - 2018 | Unknown | Unknown | Antigen detection; Nucleic acid detection | Coverage-based | Week |
| Yamagami, 2019 [[202]](https://paperpile.com/c/zPIKrB/hfrAP) | Japan | Kagoshima prefecture | 130·9, 31·4 | 2012 - 2018 | Unknown | Unknown | Antigen detection; Nucleic acid detection | Coverage-based | Week |
| Yamagami, 2019 [[202]](https://paperpile.com/c/zPIKrB/hfrAP) | Japan | Kanagawa prefecture | 139·3, 35·5 | 2012 - 2018 | Unknown | Unknown | Antigen detection; Nucleic acid detection | Coverage-based | Week |
| Yamagami, 2019 [[202]](https://paperpile.com/c/zPIKrB/hfrAP) | Japan | Kochi prefecture | 133·3, 33·5 | 2012 - 2018 | Unknown | Unknown | Antigen detection; Nucleic acid detection | Coverage-based | Week |
| Yamagami, 2019 [[202]](https://paperpile.com/c/zPIKrB/hfrAP) | Japan | Kumamoto prefecture | 130·8, 32·9 | 2012 - 2018 | Unknown | Unknown | Antigen detection; Nucleic acid detection | Coverage-based | Week |
| Yamagami, 2019 [[202]](https://paperpile.com/c/zPIKrB/hfrAP) | Japan | kyoto prefecture | 135·5, 35·2 | 2012 - 2018 | Unknown | Unknown | Antigen detection; Nucleic acid detection | Coverage-based | Week |
| Yamagami, 2019 [[202]](https://paperpile.com/c/zPIKrB/hfrAP) | Japan | Mie prefecture | 136, 33·8 | 2012 - 2018 | Unknown | Unknown | Antigen detection; Nucleic acid detection | Coverage-based | Week |
| Yamagami, 2019 [[202]](https://paperpile.com/c/zPIKrB/hfrAP) | Japan | Miyagi prefecture | 141·1, 38·6 | 2012 - 2018 | Unknown | Unknown | Antigen detection; Nucleic acid detection | Coverage-based | Week |
| Yamagami, 2019 [[202]](https://paperpile.com/c/zPIKrB/hfrAP) | Japan | Miyazaki prefecture | 131·4, 32·6 | 2012 - 2018 | Unknown | Unknown | Antigen detection; Nucleic acid detection | Coverage-based | Week |
| Yamagami, 2019 [[202]](https://paperpile.com/c/zPIKrB/hfrAP) | Japan | Nagano prefecture | 137·9, 36·2 | 2012 - 2018 | Unknown | Unknown | Antigen detection; Nucleic acid detection | Coverage-based | Week |
| Yamagami, 2019 [[202]](https://paperpile.com/c/zPIKrB/hfrAP) | Japan | Nagasaki prefecture | 129·7, 33·2 | 2012 - 2018 | Unknown | Unknown | Antigen detection; Nucleic acid detection | Coverage-based | Week |
| Yamagami, 2019 [[202]](https://paperpile.com/c/zPIKrB/hfrAP) | Japan | Nara prefecture | 135·8, 34·3 | 2012 - 2018 | Unknown | Unknown | Antigen detection; Nucleic acid detection | Coverage-based | Week |
| Yamagami, 2019 [[202]](https://paperpile.com/c/zPIKrB/hfrAP) | Japan | Niigata prefecture | 138·9, 37·5 | 2012 - 2018 | Unknown | Unknown | Antigen detection; Nucleic acid detection | Coverage-based | Week |
| Yamagami, 2019 [[202]](https://paperpile.com/c/zPIKrB/hfrAP) | Japan | Oita prefecture | 131·4, 33·2 | 2012 - 2018 | Unknown | Unknown | Antigen detection; Nucleic acid detection | Coverage-based | Week |
| Yamagami, 2019 [[202]](https://paperpile.com/c/zPIKrB/hfrAP) | Japan | Okayama prefecture | 133·6, 34·9 | 2012 - 2018 | Unknown | Unknown | Antigen detection; Nucleic acid detection | Coverage-based | Week |
| Yamagami, 2019 [[202]](https://paperpile.com/c/zPIKrB/hfrAP) | Japan | Okinawa prefecture | 127·7, 26·1 | 2012 - 2018 | Unknown | Unknown | Antigen detection; Nucleic acid detection | Coverage-based | Week |
| Yamagami, 2019 [[202]](https://paperpile.com/c/zPIKrB/hfrAP) | Japan | Osaka prefecture | 135·6, 34·6 | 2012 - 2018 | Unknown | Unknown | Antigen detection; Nucleic acid detection | Coverage-based | Week |
| Yamagami, 2019 [[202]](https://paperpile.com/c/zPIKrB/hfrAP) | Japan | Saga prefecture | 130·2, 33·3 | 2012 - 2018 | Unknown | Unknown | Antigen detection; Nucleic acid detection | Coverage-based | Week |
| Yamagami, 2019 [[202]](https://paperpile.com/c/zPIKrB/hfrAP) | Japan | Saitama prefecture | 139·4, 36 | 2012 - 2018 | Unknown | Unknown | Antigen detection; Nucleic acid detection | Coverage-based | Week |
| Yamagami, 2019 [[202]](https://paperpile.com/c/zPIKrB/hfrAP) | Japan | Shiga prefecture | 136·1, 35·3 | 2012 - 2018 | Unknown | Unknown | Antigen detection; Nucleic acid detection | Coverage-based | Week |
| Yamagami, 2019 [[202]](https://paperpile.com/c/zPIKrB/hfrAP) | Japan | Shimane prefecture | 132·6, 35·1 | 2012 - 2018 | Unknown | Unknown | Antigen detection; Nucleic acid detection | Coverage-based | Week |
| Yamagami, 2019 [[202]](https://paperpile.com/c/zPIKrB/hfrAP) | Japan | Shizuoka prefecture | 138·3, 35·1 | 2012 - 2018 | Unknown | Unknown | Antigen detection; Nucleic acid detection | Coverage-based | Week |
| Yamagami, 2019 [[202]](https://paperpile.com/c/zPIKrB/hfrAP) | Japan | Tochigi prefecture | 139·9, 36·7 | 2012 - 2018 | Unknown | Unknown | Antigen detection; Nucleic acid detection | Coverage-based | Week |
| Yamagami, 2019 [[202]](https://paperpile.com/c/zPIKrB/hfrAP) | Japan | Tokushima prefecture | 134·3, 33·9 | 2012 - 2018 | Unknown | Unknown | Antigen detection; Nucleic acid detection | Coverage-based | Week |
| Yamagami, 2019 [[202]](https://paperpile.com/c/zPIKrB/hfrAP) | Japan | Tokyo | 139·7, 35·7 | 2012 - 2018 | Unknown | Unknown | Antigen detection; Nucleic acid detection | Coverage-based | Week |
| Yamagami, 2019 [[202]](https://paperpile.com/c/zPIKrB/hfrAP) | Japan | Tottori prefecture | 133·4, 35·4 | 2012 - 2018 | Unknown | Unknown | Antigen detection; Nucleic acid detection | Coverage-based | Week |
| Yamagami, 2019 [[202]](https://paperpile.com/c/zPIKrB/hfrAP) | Japan | Toyama prefecture | 137·2, 36·7 | 2012 - 2018 | Unknown | Unknown | Antigen detection; Nucleic acid detection | Coverage-based | Week |
| Yamagami, 2019 [[202]](https://paperpile.com/c/zPIKrB/hfrAP) | Japan | Wakayama prefecture | 135·4, 33·9 | 2012 - 2018 | Unknown | Unknown | Antigen detection; Nucleic acid detection | Coverage-based | Week |
| Yamagami, 2019 [[202]](https://paperpile.com/c/zPIKrB/hfrAP) | Japan | Yamagata prefecture | 140·1, 38·5 | 2012 - 2018 | Unknown | Unknown | Antigen detection; Nucleic acid detection | Coverage-based | Week |
| Yamagami, 2019 [[202]](https://paperpile.com/c/zPIKrB/hfrAP) | Japan | Yamaguchi prefecture | 131·5, 34·3 | 2012 - 2018 | Unknown | Unknown | Antigen detection; Nucleic acid detection | Coverage-based | Week |
| Yamagami, 2019 [[202]](https://paperpile.com/c/zPIKrB/hfrAP) | Japan | Yamanashi prefecture | 138·6, 35·7 | 2012 - 2018 | Unknown | Unknown | Antigen detection; Nucleic acid detection | Coverage-based | Week |
| Yokosawa, 2006 [[203]](https://paperpile.com/c/zPIKrB/k2Xb) | Brazil | Uberlândia | -48·3, -18·9 | 2001 - 2004 | outpatient; inpatient | ARI or ILI | Antigen detection | Qualitative | Month |
| Yorita, 2007 [[204]](https://paperpile.com/c/zPIKrB/29BE) | USA | Hawaii | -155·6, 19·9 | 1997 - 2004 | inpatient | ALRI | Unknown | Qualitative | Month |
| Yu, 2019 [[205]](https://paperpile.com/c/zPIKrB/5XvIE) | China | Beijing | 116·4, 39·9 | 2007 - 2015 | inpatient | ALRI | Nucleic acid detection | Threshold-based | Week |
| Yu, 2019 [[205]](https://paperpile.com/c/zPIKrB/5XvIE) | China | Beijing | 116·4, 39·9 | 2007 - 2015 | inpatient | ALRI | Nucleic acid detection | Threshold-based | Week |
| Yu, 2019 [[205]](https://paperpile.com/c/zPIKrB/5XvIE) | China | Beijing | 116·4, 39·9 | 2007 - 2015 | inpatient | ALRI | Nucleic acid detection | Threshold-based | Week |
| Yusuf, 2007 [[206]](https://paperpile.com/c/zPIKrB/riwp) | Canada | Winnipeg | -97·1, 49·9 | 2002 - 2004 | Unknown | Unknown | Virus detection; Antigen detection | Qualitative | Month |
| Yusuf, 2007 [[206]](https://paperpile.com/c/zPIKrB/riwp) | Chile | Santiago | -70·7, -33·4 | 1999 - 2003 | Unknown | Unknown | Virus detection; Antigen detection | Qualitative | Month |
| Yusuf, 2007 [[206]](https://paperpile.com/c/zPIKrB/riwp) | USA | Buffalo | -78·9, 42·9 | 1995 - 2002 | Unknown | Unknown | Virus detection; Antigen detection | Qualitative | Month |
| Yusuf, 2007 [[206]](https://paperpile.com/c/zPIKrB/riwp) | USA | Houston | -95·4, 29·8 | 1999 - 2002 | Unknown | Unknown | Virus detection; Antigen detection | Qualitative | Month |
| Yusuf, 2007 [[206]](https://paperpile.com/c/zPIKrB/riwp) | USA | Miami | -80·2, 25·8 | 2000 - 2003 | Unknown | Unknown | Virus detection; Antigen detection | Qualitative | Month |
| Yusuf, 2007 [[206]](https://paperpile.com/c/zPIKrB/riwp) | USA | Tucson | -111, 32·2 | 1999 - 2003 | Unknown | Unknown | Virus detection; Antigen detection | Qualitative | Month |
| Zhang, 2010 [[207]](https://paperpile.com/c/zPIKrB/6P2a) | China | Chongqing | 106·6, 29·6 | 2006 - 2009 | inpatient | ARI or ILI | Nucleic acid detection | Qualitative | Month |
| Zhang, 2013 [[208]](https://paperpile.com/c/zPIKrB/K43M) | China | Suzhou | 120·6, 31·3 | 2001 - 2011 | inpatient | ARI or ILI | Antigen detection | Qualitative | Month |
| Zhao, 2014 [[209]](https://paperpile.com/c/zPIKrB/rGdI) | United Kingdom | nationwide | -1·6, 52·7 | 2009 - 2012 | Unknown | Unknown | Nucleic acid detection | Qualitative | Month |
| Zlateva, 2007 [[210]](https://paperpile.com/c/zPIKrB/vjMv) | Belgium | Leuven | 4·7, 50·9 | 1996 - 2006 | outpatient; inpatient | SARI | Virus detection; Antigen detection; Nucleic acid detection | Qualitative | Month |

Note: ARI, acute respiratory infection; ILI, influenza-like illness; SARI, severe acute respiratory infection; ALRI, acute lower respiratory infection

[**Supplementary Table**](https://docs.google.com/document/d/1I4dWqDo4EhvkiX1s2J-0H5fuGgVyt68VTcB-H1RzDxY/edit#stabl_3) **2.** Methods used to determine the timing (the start, peak and end) of RSV seasons in the included publications.

| **Method/Method Category** | **Number of studies (n=59)** | **References** |
| --- | --- | --- |
| Threshold-based method | 51 | [7,14,16,17,22,28,29,33,36–38,44,47,57,61,65,66,69,71,73, 75–77,80–83,93,99,106,110,111,124,125,127,135,138,142, 145,147,149,154,155,157,159,160,165,176,182,189,191,199,  201,205] |
| Coverage-based methods  (AAP, Search Index, MEM) | 8 | [2,3,31,77,109,191,192,202] |
| Model-based methods  (Change point model, Over-dispersed Poisson regression, Expectation-based Poisson scan statistics, Time series methods) | 6 | [4,5,107,129,139,146] |

[**Supplementary Table**](https://docs.google.com/document/d/1I4dWqDo4EhvkiX1s2J-0H5fuGgVyt68VTcB-H1RzDxY/edit#stabl_4) **3**. Characteristics of the threshold-based methods used to determine the season of RSV activity.

| **Method description** | | | **Number of studies (n=59)** | **References** |
| --- | --- | --- | --- | --- |
| **Prime indicator** | **Threshold** | **Other requirements** |  |  |
| Positive percentage | 10% | None | 6 | [[7,28,99,106,111,205]](https://paperpile.com/c/zPIKrB/T5MU6+UJyei+XkHEI+vDVJf+5XvIE+HZLo) |
|  |  | continuity^a^ ≥ 2 | 10 | [[33,44,81–83,110,135,142,149,154]](https://paperpile.com/c/zPIKrB/GMmUc+yqMda+VCs9C+80EEw+u2aTv+7vWjg+HrPoU+h4sVb+3NLPe+Ox5th) |
|  |  | continuity ≥ 2, positive samples ≥ 2 | 5 | [[65,73,80,138,165]](https://paperpile.com/c/zPIKrB/QzfFW+o3kmc+23GWJ+Raleq+VnTAx) |
|  |  | continuity ≥ 2, tests ≥ 11 | 2 | [[17,124]](https://paperpile.com/c/zPIKrB/aBsPn+gWGAj) |
|  |  | tests ≥ 10 | 2 | [[125,199]](https://paperpile.com/c/zPIKrB/br0sv+pdOs) |
|  |  | tests ≥ 20 | 3 | [[145,147,182]](https://paperpile.com/c/zPIKrB/6twje+ChHdE+dkxqZ) |
|  | 1% | None | 1 | [[61]](https://paperpile.com/c/zPIKrB/CpdqX) |
|  | 3% | continuity ≥ 2 | 3 | [[17,77,127]](https://paperpile.com/c/zPIKrB/gWGAj+1iYBm+LX59k) |
|  | 5% | None | 1 | [[77]](https://paperpile.com/c/zPIKrB/1iYBm) |
|  |  | continuity ≥ 2 | 1 | [[38]](https://paperpile.com/c/zPIKrB/42Pvu) |
|  | 7% | None | 1 | [[77]](https://paperpile.com/c/zPIKrB/1iYBm) |
|  | mean of the 5-week moving average | continuity >=3 | 1 | [[160]](https://paperpile.com/c/zPIKrB/i2Xbo) |
|  | threefold of the median | None | 1 | [[57]](https://paperpile.com/c/zPIKrB/vNcVL) |
|  | mean | continuity ≥ 2 | 1 | [[29]](https://paperpile.com/c/zPIKrB/StFDy) |
| RSV cases | 0 | continuity ≥ 3 | 1 | [[155]](https://paperpile.com/c/zPIKrB/lp0rv) |
|  | 2 (per week)  5 (per month) | None | 2 | [[157,176]](https://paperpile.com/c/zPIKrB/uo77Z+KGT1J) |
|  | 20 | None | 1 | [[191]](https://paperpile.com/c/zPIKrB/6BC5G) |
|  | 100 | None | 2 | [[69,76]](https://paperpile.com/c/zPIKrB/XvW5a+kKmQz) |
|  | 200 | None | 2 | [[69,71]](https://paperpile.com/c/zPIKrB/rMNIq+kKmQz) |
|  | 10% of peak | continuity ≥ 2 | 1 | [[82]](https://paperpile.com/c/zPIKrB/GMmUc) |
|  | 5% of the total number | continuity ≥ 2 | 1 | [[22]](https://paperpile.com/c/zPIKrB/Z8Prv) |
|  | 1·2% of the total cases of that year | None | 3 | [[36,77,191]](https://paperpile.com/c/zPIKrB/6BC5G+1iYBm+Alr49) |
|  | 60% of weekly average cases of that year | None | 2 | [[66,77]](https://paperpile.com/c/zPIKrB/YkTtU+1iYBm) |
|  | two times the average weekly number | None | 1 | [[22]](https://paperpile.com/c/zPIKrB/Z8Prv) |
|  | two times the average weekly number | cases ≥ 5 | 1 | [[47]](https://paperpile.com/c/zPIKrB/sdUN2) |
|  | 10 times the 4-week  moving average at week 29 | continuity ≥ 2 | 1 | [[127]](https://paperpile.com/c/zPIKrB/LX59k) |
|  | 1.24-fold mean of previous 5 months | None | 1 | [[14]](https://paperpile.com/c/zPIKrB/9fNn0) |
| Normalized increase | 10 | continuity ≥ 2 | 2 | [[127,159]](https://paperpile.com/c/zPIKrB/LX59k+l3Uth) |
| RSV-positive hospitalization rate | 10% | continuity ≥ 2, tests > 5 | 1 | [[37]](https://paperpile.com/c/zPIKrB/OpAug) |
|  | monthly average hospitalization rate | None | 1 | [[93]](https://paperpile.com/c/zPIKrB/I3rwW) |
| RSV-associated hospitalization | 2 | continuity ≥ 2 | 1 | [[189]](https://paperpile.com/c/zPIKrB/vwxwK) |
|  | 2% (week) or 8% (month) of annual hospitalization | None | 1 | [[201]](https://paperpile.com/c/zPIKrB/u8hZN) |
|  | the baseline (mean of June, July, and August during the period of study) plus 2 standard deviations (SD). | None | 1 | [[75]](https://paperpile.com/c/zPIKrB/nwDvF) |
| IRR or OR | 1  (statistically significant) | continuity ≥ 3 | 1 | [[16]](https://paperpile.com/c/zPIKrB/tl6A9) |

^a^ continuity means the minimum consecutive weeks/months meeting certain requirements were necessary to determine a season start.

**Supplementary Table 4.** Descriptions of RSV seasons of the pattern “two peaks a year” reported by the included publications.

| **Site** | **Study** | **Years** | **Peak1** | **Peak2** |
| --- | --- | --- | --- | --- |
| Hong Kong | Mak, 2012 [[121]](https://paperpile.com/c/zPIKrB/J6R1R) | 2004 - 2011 | March - April | July - September |
|  | Tang, 2010 [[175]](https://paperpile.com/c/zPIKrB/wB8zW) | 2000 - 2007 | March - May | July - August |
|  | Chan, 2015 [[47]](https://paperpile.com/c/zPIKrB/sdUN2) | 1998 - 2012 | Week 10-15 | Week 29-38 |
| Shenzhen, China | He, 2014 [[84]](https://paperpile.com/c/zPIKrB/m7VoW) | 2007 - 2010 | March - May | November - December |
| Guangzhou, China | Liu, 2019 [[114]](https://paperpile.com/c/zPIKrB/rm9oD) | 2009 - 2016 | February - April | August - October |
| Dadaab, Kenya | Nyoka, 2017 [[140]](https://paperpile.com/c/zPIKrB/hdTg7) | 2007 - 2011 | October - January | May - July |
| Taiwan | Hsu, 2014 [[93]](https://paperpile.com/c/zPIKrB/I3rwW) | 2000 - 2010 | April | September |
|  | Chi, 2011 [[50]](https://paperpile.com/c/zPIKrB/osdms) | 2004-2007 | Spring | Autumn |
| Miami | Yusuf, 2007 [[206]](https://paperpile.com/c/zPIKrB/riwp) | 2000-2003 | Week 38-42 | January and February |
| Bardados | Li, 2019 [[3]](https://paperpile.com/c/zPIKrB/BQ74y) | 2010-2017 | February - March | July - October |
| Salvador, Brazil | Li, 2019 [[3]](https://paperpile.com/c/zPIKrB/BQ74y) | 2009-2013 | March - May | October - December |

**Supplementary Table 5.** Description of RSV seasons of the pattern “two-year cycle” reported by the included publications.

| **Site** | **Study** | **Years** | **Description** |
| --- | --- | --- | --- |
| Finland | Waris, 1991 [[211]](https://paperpile.com/c/zPIKrB/aDK44) | 1981-1990 | A 2-year cycle beginning in December or January: a minor peak in the spring and a major peak in the next winter. |
|  | Renko, 2019 [[129]](https://paperpile.com/c/zPIKrB/hD5ZO) | 1995-2006 | Every odd year a small spring epidemic was followed by a large epidemic peaking around December. |
| Vienna, Austria | Aberle, 2008 [[6]](https://paperpile.com/c/zPIKrB/xZQ74) | 2000 - 2007 | Early RSV seasons with peak activity in January and December were followed by late seasons with peak activity in February and March. |
| Stockholm, Sweden | Eriksson, 2002 [[63]](https://paperpile.com/c/zPIKrB/j8Al7) | 1987 - 1998 | There was a pattern of early large and late small epidemic seasons alternating biannually during the entire 12-y period. |
|  | Reyes, 1997 [[157]](https://paperpile.com/c/zPIKrB/uo77Z) | 1984-1994 | The seasonal pattern varied every other year, with late (peaks in weeks 13-18) epidemics followed by early (peaks in weeks 49-5) ones. The number of detected cases was significantly greater, around twice as many, during early than during late epidemics. |
| Kiel, Germany | Weigl, 2002 [[197]](https://paperpile.com/c/zPIKrB/7fCuW) | 1998 - 2001 | From 1997/98 onwards, a 2-year pattern with a late season starting between December and February followed by an early season starting in September to October was ob-served. Taking data from 1994 onwards into consideration the season regularly started late until 1998/99. |
|  | Weigl, 2007 [[212]](https://paperpile.com/c/zPIKrB/G5GoC) | 1996-2006 | In uneven ey, the RSV season started late at the end of December to January and was less severe than in even ey when the season started early at the end of September to October with a high incidence. |
| Stuttgart, Germany | Terletskaia-Ladwig, 2005 [[176]](https://paperpile.com/c/zPIKrB/KGT1J) | 1996-2001 | An early season with strong RSV activity (early-high phase) was followed by a weaker late season (late-low phase) in a regular biennial rhythm. |
| Germany | Reiche, 2009 [[155]](https://paperpile.com/c/zPIKrB/lp0rv) | 1998 - 2007 | A regular 2-year cyclic pattern was observed for two consecutive late and early seasons. |
|  | Obando-Pacheco, 2018 [[142]](https://paperpile.com/c/zPIKrB/h4sVb) | 2010-2017 | An early season starting in October–November and finishing in March–April and a late season starting in December and finishing in May, with both seasons having a similar duration. |
| Denmark | Jepsen, 2018 [[98]](https://paperpile.com/c/zPIKrB/EaElK) | 2010 - 2015 | Every other year the RSV season had an early start in weeks 46 to 48, and was rather mild in terms of hospitalisations. Every alternating year the RSV-season would start a few weeks later (week 50–52) and be characterized with a markedly higher RSV-hospitalization incidence. |
| Zagreb County, Croatia | [Mlinaric-Galinovic](https://pubmed.ncbi.nlm.nih.gov/?term=Mlinaric-Galinovic+G&cauthor_id=18226194), 2008 [[131]](https://paperpile.com/c/zPIKrB/otzRz) | 1994-2004 | RSV epidemics peaked in December/January of years 1994/95, 1996/97, 1998/99, 2000/01, 2002/ 03, and 2004/05 ("large seasons"), but in March/April of years 1996, 1998, 2000, 2002, and 2004 ("small seasons") |
| Switzerland | Duppenthaler,2003 [[61]](https://paperpile.com/c/zPIKrB/CpdqX) | 1997-2001 | The two minor epidemics were characterized by late onset, late peak , late end and low hospitalization rates. The major epidemics began early, peaked early, ended by week 14 and caused two to fourfold higher hospitalization rates. |
| Bismarck, USA | Irmen, 2000 [[97]](https://paperpile.com/c/zPIKrB/xcSo5) | 1987 - 1998 | The incidence of RSV tended to be higher and the month of peak RSV activity seemed to occur earlier (December through February) during the epidemic following a short interval (2- to 5-month), compared with the incidence and peak activity occurring later (March through May) following a long interval (7- to 9- month). The long interval and short interval were alternating. |
| Salt Lake County, USA | Leecaster, 2011 [[108]](https://paperpile.com/c/zPIKrB/nwSk) | 2001-2008 | The biennial variation in our seasonal epidemic data was seen in the early exponential growth rates (slope of the cumulative case curves[)](https://www.ncbi.nlm.nih.gov/pmc/articles/PMC3094225/figure/F1/) as well as total epidemic size. |
| Perth, Australia | Moore, 2009 [[133]](https://paperpile.com/c/zPIKrB/mAtYr) | 1997-2005 | The RSV identification rate showed consistent biennial peaks in even-numbered years. |

**Supplementary Table 6.** Descriptions of RSV of “unclear pattern” reported by the included publications.

| **Site** | **Study** | **Years** | **Description** |
| --- | --- | --- | --- |
| Rio de Janeiro, Brazil | Sutmoller, 1995 [[172]](https://paperpile.com/c/zPIKrB/f9vki) | 1987-1989 | During the 3-year period, infection with RSV was clearly seasonal: increases in the late fall and winter were observed, except in 1989, when two peaks oc- curred-one in the first quarter (January-March), and one in the third quarter (July-September) |
| Nova Scotia, Canada | AI-Assam, 2009 [[8]](https://paperpile.com/c/zPIKrB/tfn1D) | 2005 - 2008 | The onset and peak periods of RSV activity varied over time, but the duration of each year’s outbreak was similar, last-ing five to six months. In 2005-2006, the RSV season started in February, peaked in April and ended in July. In 2006-2007, the onset was in December, with a peak in February and ending in late May. During 2007-2008 there were two peak periods (February/March) and (May/June) with the epidemic ending in late June. |
| Hong Kong | Liu, 2019[[114]](https://paperpile.com/c/zPIKrB/rm9oD) | 2006-2008 | There was no definite seasonality,but incidence was lowest between October and January. |
| Dhaka, Bangladesh | Stockman, 2013[[168]](https://paperpile.com/c/zPIKrB/9Yq61) | 2004 - 2008 | Annual data suggests RSV activity occurred during defined periods lasting approximately three months with no clear seasonal pattern. During the 4 study years there was one peak in January. 2006, July 2006 and October 2007. There were at least 2 months with low RSV detections between peaks. |
| Kimberley, Australia | Hogan, 2016 [[89]](https://paperpile.com/c/zPIKrB/VsIIl) | 2000-2013 | There is a less identifiable seasonal pattern in the Pilbara region in the state’s north, and no seasonal peak evident in the Kimberley region. |
| Pilbara, Australia |  |  |  |
| Nairobi, Kenya | Rose, 2020 [[160]](https://paperpile.com/c/zPIKrB/i2Xbo) | 2006-2018 | RSV did not have a clear pattern of circulation in Nairobi, and we could not define sea- son onset, offset, or peak for that region. |
| Mexico | Obando-Pacheco, 2018 [[142]](https://paperpile.com/c/zPIKrB/h4sVb) | 2012-2015 | A 2-season year is followed by a milder year, where the outbreak starts in spring and activity is maintained almost all year round with no clear peaks. |

[**Supplementary Table**](https://docs.google.com/document/d/1PIbnf9Bu1RZLooXKvEq9IuGhpqv_UyyLBUtGsyH_5B0/edit#stabl_2) **7.** Correlation analysis on the association between duration of RSV season and latitude, climatic zone, and daily average mean temperature and the absolute humidity.

| **Variable** | **Hemisphere** | **Climatic**  **zone**^b^ | **All estimates** | | **Estimates**  **from qualitative methods** | | **Estimates**  **from quantitative methods** | |
| --- | --- | --- | --- | --- | --- | --- | --- | --- |
|  |  |  | **Correlation coefficient** | **p-value** | **Correlation coefficient** | **p-value** | **Correlation coefficient** | **p-value** |
| **Latitude** ^a^ | **Northern**  **Hemisphere** | **All** | -0·38 (-0·49, -0·25) | <0·001 | -0·08 (-0·33, 0·19) | 0·60 | -0·47 (-0·59, -0·33) | <0·001 |
|  |  | **Temperate** | -0·31 (-0·44, -0·17) | <0·001 | -0·06 (-0·35, 0·24) | 0·81 | -0·39 (-0·52, -0·23) | <0·001 |
|  | **Southern**  **Hemisphere** | **All** | -0·46 (-0·67, -0·18) | 0·0020 | -0·48 (-0·27, 0·87) | 0·87 | -0·45 (-0·69, -0·13) | <0·0083 |
|  |  | **Temperate** | -0·39 (-0·68, -0·01) | 0·056 | -0·48 (-0·54, 0·93) | 0·93 | 0·037 (-0·43, 0·48) | 0·88 |
|  | **Both** | **All** | -0·36 (-0·46, -0·24) | <0·001 | -0·15 (-0·38, 0·09) | 0·22 | -0·41 (-0·53, -0·28) | <0·001 |
|  |  | **Temperate** | -0·29 (-0·41, -0·16) | <0·001 | -0·09 (-0·36, 0·19) | 0·51 | -0·35 (-0·48, -0·20) | <0·001 |
| **Daily average mean temperature** | **Northern**  **Hemisphere** | **All** | 0·33 (0·20, 0·45) | <0·001 | 0·008 (-0·26, 0·27) | 0·95 | 0·42 (0·27, 0·54) | <0·001 |
|  |  | **Temperate** | 0·29 (0·14, 0·42) | <0·001 | -0·036 (-0·33, 0·26) | 0·82 | 0·36 (0·20, 0·50) | <0·001 |
|  | **Southern**  **Hemisphere** | **All** | 0·49 (0·22, 0·69) | 0·001 | 0·53 (-0·21, 0·88) | 0·1 | 0·48 (0·17, 0·71) | 0·0046 |
|  |  | **Temperate** | 0·40 (0·005, 0·69) | 0·048 | 0·63 (-0·37, 0·95) | 0·18 | 0·071 (-0·40, 0·51) | 0·77 |
|  | **Both** | **All** | 0·33 (0·22, 0·44) | <0·001 | 0·07 (-0·177, 0·308) | 0·58 | 0·39 (0·26, 0·51) | <0·001 |
|  |  | **Temperate** | 0·28 (0·14, 0·40) | <0·001 | -0·005 (-0·28, 0·27) | 0·97 | 0·34 (0·19, 0·47) | <0·001 |
| **Daily average mean absolute humidity** | **Northern**  **Hemisphere** | **All** | 0·31 (0·18, 0·43) | <0·001 | 0·068 (-0·20, 0·33) | 0·62 | 0·39 (0·24, 0·52) | <0·001 |
|  |  | **Temperate** | 0·29 (0·14, 0·42) | <0·001 | 0·014 (-0·28, 0·31) | 0·93 | 0·35 (0·19, 0·50) | <0·001 |
|  | **Southern**  **Hemisphere** | **All** | 0·55 (0·30, 0·73) | <0·001 | 0·53 (-0·21, 0·88) | 0·15 | 0·53 (0·23, 0·74) | 0·0016 |
|  |  | **Temperate** | 0·20 (-0·21, 0·55) | 0·34 | 0·48 (-0·55, 0·93) | 0·34 | -0·55 (-0·80, -0·12) | 0·016 |
|  | **Both** | **All** | 0·34 (0·22, 0·45) | <0·001 | 0·16 (-0·092, 0·39) | 0·22 | 0·39 (0·26, 0·51) | <0·001 |
|  |  | **Temperate** | 0·272 (0·14, 0·40) | <0·001 | 0·085 (-0·20, 0·35) | 0·55 | 0·32 (0·16, 0·46) | <0·001 |
| **Climatic zone**^c^ | **Northern**  **Hemisphere** | **All** | 0·29 | <0·001 | -0·034 | 0·77 | 0·36 | <0·001 |
|  |  | **Temperate** | 0·21 | <0·001 | -0·13 | 0·33 | 0·28 | <0·001 |
|  | **Southern**  **Hemisphere** | **All** | 0·29 | 0·018 | 0·18 | 0·59 | 0·29 | 0·042 |
|  |  | **Temperate** | 0·075 | 0·66 | NA | NA | -0·043 | 0·83 |
|  | **Both** | **All** | 0·28 | <0·001 | 0·024 | 0·82 | 0·33 | <0·001 |
|  |  | **Temperate** | 0·19 | 0·0014 | -0·094 | 0·46 | 0·24 | <0·001 |
| **Analytic methods**^d^ | **Northern**  **Hemisphere** | **All** | 0·24 | <0·001 | NA | NA | 0·34 | <0·001 |
|  |  | **Temperate** | 0·31 | <0·001 | NA | NA | 0·43 | <0·001 |
|  | **Southern**  **Hemisphere** | **All** | 0·26 | 0·12 | NA | NA | 0·25 | 0·090 |
|  |  | **Temperate** | 0·32 | 0·15 | NA | NA | 0·42 | 0·035 |
|  | **Both** | **All** | 0·25 | <0·001 | NA | NA | 0·32 | <0·001 |
|  |  | **Temperate** | 0·31 | <0·001 | NA | NA | 0·42 | <0·001 |

^a^ The absolute value of the latitude coordinate was used in the correlation analysis.

^b^ In the column of Climatic zone, Temperate includes the estimates from regions where the latitude > 23·5 or <-23·5.

^c^ Climate zone includes tropics (-23·5 ° – 23·5 °), subtropics, (-35 ° – -23·5 °, 23·5 ° – 35 °), and temperate zone (> 35 °, <-35 °) Kendall's tau statistic was used to evaluate the correlations.

^d^ Analytic methods used for estimating RSV seasonality were categorized into four groups, including qualitative methods, and three groups of quantitative methods, i.e., threshold-based methods, coverage-based methods, and model-based methods (details see Supplementary material). Kendall's tau statistic was used to evaluate the correlations.

[**Supplementary Table**](https://docs.google.com/document/d/1PIbnf9Bu1RZLooXKvEq9IuGhpqv_UyyLBUtGsyH_5B0/edit#stabl_2) **8**. Associations identified in the linear regression analysis between durations of RSV seasons and study characteristics from all investigations included in the review.

| **Variable** | **Change in the duration of RSV season（months）per unit change or compared to the reference group** | **95% confidence interval** | **p-value** | **Adjusted R squared** |
| --- | --- | --- | --- | --- |
| **Climatic zone** |  |  |  |  |
| Temperate zone  (<-35° or >35°) | 0 | Referent |  |  |
| Subtropical region  (-35°- -23·5° or 23·5°- 35°) | 0·69 | 0·21, 1·18 |  |  |
| Tropical region (-23·5°– 23·5°) | 1·15 | 0·42, 1·88 |  |  |
| **Analysis Method** |  |  |  |  |
| Coverage-based method | 0 | Referent |  |  |
| Qualitative method | 1·02 | 0·52, 1·51 | <0·001 |  |
| Threshold-based method | 1·41 | 0·96, 1·86 | <0·001 |  |
| Model-based method | 1·80 | -1·10, 4·70 | 0·20 |  |
| **Meteorological Factor** |  |  |  |  |
| Daily average mean absolute humidity | 0·011 | -0·0028, 0·026 | 0·19 |  |

^a^ The absolute value of the latitude coordinate was used in the regression analysis.

**Supplementary Table 9.** Associations identified in the linear regression analysis with the alternatrive model (using latitude instead of climatic zone) between durations of RSV seasons estimated from quantitative approaches and characteristics of the studies.

| **Variable** | **Change in the duration of RSV season (months) per unit change or compared to the reference group** | **95% confidence Interval** | **p-value** | **Adjusted R^2^** |
| --- | --- | --- | --- | --- |
| **Geocode** |  |  |  | 0·31 |
| Latitude^a^ | -0·041 | -0·064, -0·017 | <0·001 |  |
| **Analysis method** |  |  |  |  |
| Coverage-based method | 0 | Referent |  |  |
| Threshold-based method | 1·35 | 0·90, 1·81 | <0·001 |  |
| Model-based method | 1·68 | -1·33, 4·69 | 0·27 |  |
| **Meteorological Factor** |  |  |  |  |
| Daily average mean absolute humidity | 0·016 | -0·0012, 0·034 | 0·067 |  |

^a^ The absolute value of the latitude coordinate was used in the regression analysis.

[**Supplementary Table**](https://docs.google.com/document/d/1PIbnf9Bu1RZLooXKvEq9IuGhpqv_UyyLBUtGsyH_5B0/edit#stabl_2) **10**. Associations identified in the linear regression analysis with alternative model (using latitude instead of climatic zone) between durations of RSV seasons and study characteristics from all investigations included in the review.

| **Variable** | **Change in the duration of RSV season（months）per unit change or compared to the reference group** | **95% confidence interval** | **p-value** | **Adjusted R squared** |
| --- | --- | --- | --- | --- |
| **Geocode** |  |  |  | 0·24 |
| Latitude | -0·030 | -0·049, -0·011 | 0·0018 |  |
| **Analysis Method** |  |  |  |  |
| Coverage-based method | 0 | Referent |  |  |
| Qualitative method | 1·02 | 0·54, 1·51 | <0·001 |  |
| Threshold-based method | 1·35 | 0·91, 1·79 | <0·001 |  |
| Model-based method | 1·61 | -1·29, 4·51 | 0·28 |  |
| **Meteorological Factor** |  |  |  |  |
| Daily average mean absolute humidity | 0·012 | -0·0019, 0·026 | 0·089 |  |

^a^ The absolute value of the latitude coordinate was used in the regression analysis.

**Supplementary Table 11.** Associations identified in the linear regression analysis with the alternatrive model (using daily average mean temperature instead of climatic zone) between durations of RSV seasons estimated from quantitative approaches and characteristics of the studies.

| **Variable** | **Change in the duration of RSV season (months) per unit change or compared to the reference group** | **95% confidence Interval** | **p-value** | **Adjusted R^2^** |
| --- | --- | --- | --- | --- |
| **Analysis method** |  |  |  | 0.28 |
| Coverage-based method | 0 | Referent |  |  |
| Threshold-based method | 1·30 | 0·83, 1·76 | <0·001 |  |
| Model-based method | 1·37 | -1·69, 4·43 | 0·38 |  |
| **Meteorological Factor** |  |  |  |  |
| Daily average mean absolute humidity | 0·022 | 0.0028, 0.041 | 0·025 |  |
| Daily average mean temperature | 0.058 | 0.0077, 0.11 | 0.024 |  |

[**Supplementary Table**](https://docs.google.com/document/d/1PIbnf9Bu1RZLooXKvEq9IuGhpqv_UyyLBUtGsyH_5B0/edit#stabl_2) **12**. Associations identified in the linear regression analysis with alternative model (using daily average mean temperature instead of climatic zone) between durations of RSV seasons and study characteristics from all investigations included in the review.

| **Variable** | **Change in the duration of RSV season（months）per unit change or compared to the reference group** | **95% confidence interval** | **p-value** | **Adjusted R squared** |
| --- | --- | --- | --- | --- |
| **Analysis Method** |  |  |  | 0.23 |
| Coverage-based method | 0 | Referent |  |  |
| Qualitative method | 0·99 | 0·50, 1·49 | <0·001 |  |
| Threshold-based method | 1·31 | 0·87, 1·75 | <0·001 |  |
| Model-based method | 1·38 | -1·55, 4·31 | 0·36 |  |
| **Meteorological Factor** |  |  |  |  |
| Daily average mean absolute humidity | 0·018 | 0·0034, 0·032 | 0·015 |  |
| Daily average mean temperature | 0.042 | 0·0031, 0·080 | 0.034 |  |

**References**

1. Google Maps Platform. [Google Geocode API. [cited 14 Nov 2022]. Available:](file:///C:\Users\ss3030081632\SPH%20Dropbox\Song%20Wei%20Shan\shared%20RSV%20Projects\RSV%20Seasonality%20Review\output\manuscript\OFID_proofreading\Google%20Geocode%20API.%20%5bcited%2014%20Nov%202022%5d.%20Available:) <https://developers.google.com/maps/documentation/geocoding>

2. [Staadegaard L, Caini S, Wangchuk S, Thapa B, de Almeida WAF, de Carvalho FC, et al. Defining the seasonality of respiratory syncytial virus around the world: National and subnational surveillance data from 12 countries. Influenza Other Respi Viruses. 2021;15: 732–741. doi:](http://paperpile.com/b/zPIKrB/aIPfL)[10.1111/irv.12885](http://dx.doi.org/10.1111/irv.12885)

3. [Li Y, Reeves RM, Wang X, Bassat Q, Brooks WA, Cohen C, et al. Global patterns in monthly activity of influenza virus, respiratory syncytial virus, parainfluenza virus, and metapneumovirus: a systematic analysis. Lancet Glob Health. 2019;7: e1031–e1045. doi:](http://paperpile.com/b/zPIKrB/BQ74y)[10.1016/S2214-109X(19)30264-5](http://dx.doi.org/10.1016/S2214-109X(19)30264-5)

4. [Nenna R, Evangelisti M, Frassanito A, Scagnolari C, Pierangeli A, Antonelli G, et al. Respiratory syncytial virus bronchiolitis, weather conditions and air pollution in an Italian urban area: An observational study. Environ Res. 2017;158: 188–193. doi:](http://paperpile.com/b/zPIKrB/JH4bW)[10.1016/j.envres.2017.06.014](http://dx.doi.org/10.1016/j.envres.2017.06.014)

5. [Callahan ZY, Smith TK, Ingersoll C, Gardner R, Korgenski EK, Sloan CD. Comparative Seasonal Respiratory Virus Epidemic Timing in Utah. Viruses. 2020;12. doi:](http://paperpile.com/b/zPIKrB/o6J01)[10.3390/v12030275](http://dx.doi.org/10.3390/v12030275)

6. [Aberle SW, Aberle JH, Sandhofer MJ, Pracher E, Popow-Kraupp T. Biennial spring activity of human metapneumovirus in Austria. Pediatr Infect Dis J. 2008;27: 1065–1068. doi:](http://paperpile.com/b/zPIKrB/xZQ74)[10.1097/INF.0b013e31817ef4fd](http://dx.doi.org/10.1097/INF.0b013e31817ef4fd)

7. [Agoti CN, Otieno JR, Ngama M, Mwihuri AG, Medley GF, Cane PA, et al. Successive Respiratory Syncytial Virus Epidemics in Local Populations Arise from Multiple Variant Introductions, Providing Insights into Virus Persistence. J Virol. 2015;89: 11630–11642. doi:](http://paperpile.com/b/zPIKrB/HZLo)[10.1128/JVI.01972-15](http://dx.doi.org/10.1128/JVI.01972-15)

8. [Al-Assam A, Langley JM, Sarwal S. Respiratory Watch: Development of a provincial system for respiratory syncytial virus surveillance in Nova Scotia, 2005-2008. Can J Infect Dis Med Microbiol. 2009;20: e153–6. doi:](http://paperpile.com/b/zPIKrB/tfn1D)[10.1155/2009/361948](http://dx.doi.org/10.1155/2009/361948)

9. [Al-Romaihi HE, Smatti MK, Ganesan N, Nadeem S, Farag E, Coyle PV, et al. Epidemiology of respiratory infections among adults in Qatar (2012-2017). PLoS One. 2019;14: e0218097. doi:](http://paperpile.com/b/zPIKrB/rqUb)[10.1371/journal.pone.0218097](http://dx.doi.org/10.1371/journal.pone.0218097)

10. [Al-Romaihi HE, Smatti MK, Al-Khatib HA, Coyle PV, Ganesan N, Nadeem S, et al. Molecular epidemiology of influenza, RSV, and other respiratory infections among children in Qatar: A six years report (2012-2017). Int J Infect Dis. 2020;95: 133–141. doi:](http://paperpile.com/b/zPIKrB/ohzb)[10.1016/j.ijid.2020.04.008](http://dx.doi.org/10.1016/j.ijid.2020.04.008)

11. [Al-Thani A, Elsheikh M, Janahi M, Al-Marri A, Caksen H, Bener A. Seasonality and epidemiology of respiratory syncytial virus in Qatar. Pediatr Infect Dis J. 2008;3: 41–45. Available:](http://paperpile.com/b/zPIKrB/x39d) <https://content.iospress.com/articles/journal-of-pediatric-infectious-diseases/jpi00102>

12. [al-Hajjar S, Akhter J, al Jumaah S, Hussain Qadri SM. Respiratory viruses in children attending a major referral centre in Saudi Arabia. Ann Trop Paediatr. 1998;18: 87–92. doi:](http://paperpile.com/b/zPIKrB/LxVi)[10.1080/02724936.1998.11747933](http://dx.doi.org/10.1080/02724936.1998.11747933)

13. [Ali A, Yousafzai MT, Waris R, Jafri F, Aziz F, Abbasi IN, et al. RSV associated hospitalizations in children in Karachi, Pakistan: Implications for vaccine prevention strategies. J Med Virol. 2017;89: 1151–1157. doi:](http://paperpile.com/b/zPIKrB/QU8n)[10.1002/jmv.24768](http://dx.doi.org/10.1002/jmv.24768)

14. [Alonso A, Andres JM, Garmendia JR, Diez I, Gil JM, Ardura J. Bronchiolitis due to respiratory syncytial virus in hospitalized children: a study of seasonal rhythm. Acta Paediatr. 2007;96: 731–735. doi:](http://paperpile.com/b/zPIKrB/9fNn0)[10.1111/j.1651-2227.2007.00266.x](http://dx.doi.org/10.1111/j.1651-2227.2007.00266.x)

15. [Alonso WJ, Laranjeira BJ, Pereira SAR, Florencio CMGD, Moreno EC, Miller MA, et al. Comparative dynamics, morbidity and mortality burden of pediatric viral respiratory infections in an equatorial city. Pediatr Infect Dis J. 2012;31: e9–14. doi:](http://paperpile.com/b/zPIKrB/YD3c)[10.1097/INF.0b013e31823883be](http://dx.doi.org/10.1097/INF.0b013e31823883be)

16. [Althouse BM, Flasche S, Minh LN, Thiem VD, Hashizume M, Ariyoshi K, et al. Seasonality of respiratory viruses causing hospitalizations for acute respiratory infections in children in Nha Trang, Vietnam. Int J Infect Dis. 2018;75: 18–25. doi:](http://paperpile.com/b/zPIKrB/tl6A9)[10.1016/j.ijid.2018.08.001](http://dx.doi.org/10.1016/j.ijid.2018.08.001)

17. [Ambrose CS, Steed LL, Brandon M, Frye K, Olajide IR, Thomson G. National and regional modeling of distinct RSV seasonality thresholds for antigen and PCR testing in the United States. J Clin Virol. 2019;120: 68–77. doi:](http://paperpile.com/b/zPIKrB/gWGAj)[10.1016/j.jcv.2019.09.010](http://dx.doi.org/10.1016/j.jcv.2019.09.010)

18. [Ampofo K, Bender J, Sheng X, Korgenski K, Daly J, Pavia AT, et al. Seasonal invasive pneumococcal disease in children: role of preceding respiratory viral infection. Pediatrics. 2008;122: 229–237. doi:](http://paperpile.com/b/zPIKrB/jlUM)[10.1542/peds.2007-3192](http://dx.doi.org/10.1542/peds.2007-3192)

19. [Anderson LJ, Parker RA, Strikas RL. Association between respiratory syncytial virus outbreaks and lower respiratory tract deaths of infants and young children. J Infect Dis. 1990;161: 640–646. doi:](http://paperpile.com/b/zPIKrB/eub9)[10.1093/infdis/161.4.640](http://dx.doi.org/10.1093/infdis/161.4.640)

20. [Appak Ö, Duman M, Belet N, Sayiner AA. Viral respiratory infections diagnosed by multiplex polymerase chain reaction in pediatric patients. J Med Virol. 2019;91: 731–737. doi:](http://paperpile.com/b/zPIKrB/iCtM)[10.1002/jmv.25379](http://dx.doi.org/10.1002/jmv.25379)

21. [Arnott A, Vong S, Mardy S, Chu S, Naughtin M, Sovann L, et al. A study of the genetic variability of human respiratory syncytial virus (HRSV) in Cambodia reveals the existence of a new HRSV group B genotype. J Clin Microbiol. 2011;49: 3504–3513. doi:](http://paperpile.com/b/zPIKrB/NNGa)[10.1128/JCM.01131-11](http://dx.doi.org/10.1128/JCM.01131-11)

22. [Assink MDM, Kiewiet JP, Rozenbaum MH, Van den Berg PB, Hak E, Buskens EJ, et al. Excess drug prescriptions during influenza and RSV seasons in the Netherlands: potential implications for extended influenza vaccination. Vaccine. 2008;27: 1119–1126. doi:](http://paperpile.com/b/zPIKrB/Z8Prv)[10.1016/j.vaccine.2008.11.070](http://dx.doi.org/10.1016/j.vaccine.2008.11.070)

23. [Azzari C, Baraldi E, Bonanni P, Bozzola E, Coscia A, Lanari M, et al. Epidemiology and prevention of respiratory syncytial virus infections in children in Italy. Ital J Pediatr. 2021;47: 198. doi:](http://paperpile.com/b/zPIKrB/Ioh4)[10.1186/s13052-021-01148-8](http://dx.doi.org/10.1186/s13052-021-01148-8)

24. [Bakir TM, Halawani M, Ramia S. Viral aetiology and epidemiology of acute respiratory infections in hospitalized Saudi children. J Trop Pediatr. 1998;44: 100–103. doi:](http://paperpile.com/b/zPIKrB/PBuo)[10.1093/tropej/44.2.100](http://dx.doi.org/10.1093/tropej/44.2.100)

25. [Balmaks R, Ribakova I, Gardovska D, Kazaks A. Molecular epidemiology of human respiratory syncytial virus over three consecutive seasons in Latvia. J Med Virol. 2013;86: 1971–1982. doi:](http://paperpile.com/b/zPIKrB/Xgw6)[10.1002/jmv.23855](http://dx.doi.org/10.1002/jmv.23855)

26. [Bandeira T, Carmo M, Lopes H, Gomes C, Martins M, Guzman C, et al. Burden and severity of children’s hospitalizations by respiratory syncytial virus in Portugal, 2015-2018. Influenza Other Respi Viruses. 2022;17. doi:](http://paperpile.com/b/zPIKrB/avkSI)[10.1111/irv.13066](http://dx.doi.org/10.1111/irv.13066)

27. [Barbati F, Moriondo M, Pisano L, Calistri E, Lodi L, Ricci S, et al. Epidemiology of Respiratory Syncytial Virus-Related Hospitalization Over a 5-Year Period in Italy: Evaluation of Seasonality and Age Distribution Before Vaccine Introduction. Vaccines (Basel). 2020;8. doi:](http://paperpile.com/b/zPIKrB/uycl)[10.3390/vaccines8010015](http://dx.doi.org/10.3390/vaccines8010015)

28. [Bauman J, Eggleston M, Oquist N, Malinoski F. Respiratory syncytial virus: seasonal data for regions of Florida and implications for palivizumab. South Med J. 2007;100: 669–676. doi:](http://paperpile.com/b/zPIKrB/T5MU6)[10.1097/SMJ.0b013e318048589e](http://dx.doi.org/10.1097/SMJ.0b013e318048589e)

29. [Baumeister E, Duque J, Varela T, Palekar R, Couto P, Savy V, et al. Timing of respiratory syncytial virus and influenza epidemic activity in five regions of Argentina, 2007-2016. Influenza Other Respi Viruses. 2018;13: 10–17. doi:](http://paperpile.com/b/zPIKrB/StFDy)[10.1111/irv.12596](http://dx.doi.org/10.1111/irv.12596)

30. [Berner R, Schwoerer F, Schumacher RF, Meder M, Forster J. Community and nosocomially acquired respiratory syncytial virus infection in a German paediatric hospital from 1988 to 1999. Eur J Pediatr. 2001;160: 541–547. doi:](http://paperpile.com/b/zPIKrB/EIBi)[10.1007/s004310100801](http://dx.doi.org/10.1007/s004310100801)

31. [Billard M-N, van de Ven PM, Baraldi B, Kragten-Tabatabaie L, Bont LJ, Wildenbeest JG. International changes in respiratory syncytial virus (RSV) epidemiology during the COVID-19 pandemic: Association with school closures. Influenza Other Respi Viruses. 2022;16: 926–936. doi:](http://paperpile.com/b/zPIKrB/aUflC)[10.1111/irv.12998](http://dx.doi.org/10.1111/irv.12998)

32. [Respiratory syncytial virus, 1979: Scotland. Br Med J. 1979;2: 1518. Available:](http://paperpile.com/b/zPIKrB/qwlJ) <https://www.ncbi.nlm.nih.gov/pubmed/526842>

33. [Boron ML, Edelman L, Groothuis JR, Malinoski FJ. A novel active respiratory syncytial virus surveillance system in the United States: variability in the local and regional incidence of infection. Pediatr Infect Dis J. 2008;27: 1095–1098. doi:](http://paperpile.com/b/zPIKrB/80EEw)[10.1097/INF.0b013e3181812c8e](http://dx.doi.org/10.1097/INF.0b013e3181812c8e)

34. [Brini I, Bhiri S, Ijaz M, Bouguila J, Nouri-Merchaoui S, Boughammoura L, et al. Temporal and climate characteristics of respiratory syncytial virus bronchiolitis in neonates and children in Sousse, Tunisia, during a 13-year surveillance. Environ Sci Pollut Res Int. 2020;27: 23379–23389. doi:](http://paperpile.com/b/zPIKrB/71Jr)[10.1007/s11356-018-3922-x](http://dx.doi.org/10.1007/s11356-018-3922-x)

35. [Brittain-Long R, Andersson L-M, Olofsson S, Lindh M, Westin J. Seasonal variations of 15 respiratory agents illustrated by the application of a multiplex polymerase chain reaction assay. Scand J Infect Dis. 2011;44: 9–17. doi:](http://paperpile.com/b/zPIKrB/jYh9)[10.3109/00365548.2011.598876](http://dx.doi.org/10.3109/00365548.2011.598876)

36. [Broberg EK, Waris M, Johansen K, Snacken R, Penttinen P, European Influenza Surveillance Network. Seasonality and geographical spread of respiratory syncytial virus epidemics in 15 European countries, 2010 to 2016. Euro Surveill. 2018;23. doi:](http://paperpile.com/b/zPIKrB/Alr49)[10.2807/1560-7917.ES.2018.23.5.17-00284](http://dx.doi.org/10.2807/1560-7917.ES.2018.23.5.17-00284)

37. [Bruden DJT, Singleton R, Hawk CS, Bulkow LR, Bentley S, Anderson LJ, et al. Eighteen Years of Respiratory Syncytial Virus Surveillance: Changes in Seasonality and Hospitalization Rates in Southwestern Alaska Native Children. Pediatr Infect Dis J. 2015;34: 945–950. doi:](http://paperpile.com/b/zPIKrB/OpAug)[10.1097/INF.0000000000000772](http://dx.doi.org/10.1097/INF.0000000000000772)

38. [Cai W, Dürrwald R, Biere B, Schweiger B, Haas W, Wolff T, et al. Determination of respiratory syncytial virus epidemic seasons by using 95% confidence interval of positivity rates, 2011-2021, Germany. Influenza Other Respi Viruses. 2022;16: 854–857. doi:](http://paperpile.com/b/zPIKrB/42Pvu)[10.1111/irv.12996](http://dx.doi.org/10.1111/irv.12996)

39. [Caini S, de Mora D, Olmedo M, Portugal D, Becerra MA, Mejía M, et al. The epidemiology and severity of respiratory viral infections in a tropical country: Ecuador, 2009–2016. J Infect Public Health. 2019;12: 357–363. doi:](http://paperpile.com/b/zPIKrB/WHKf)[10.1016/j.jiph.2018.12.003](http://dx.doi.org/10.1016/j.jiph.2018.12.003)

40. [Caini S, Stolyarov K, Sominina A, Smorodintseva E, Staadegaard L, Paget J, et al. A comparative analysis of the epidemiology of influenza and respiratory syncytial virus in Russia, 2013/14 to 2018/19. J Glob Health. 2022;12: 04009. doi:](http://paperpile.com/b/zPIKrB/DKVK)[10.7189/jogh.12.04009](http://dx.doi.org/10.7189/jogh.12.04009)

41. [Calvo C, Pozo F, García-García ML, Sanchez M, Lopez-Valero M, Pérez-Breña P, et al. Detection of new respiratory viruses in hospitalized infants with bronchiolitis: a three-year prospective study. Acta Paediatr. 2010;99: 883–887. doi:](http://paperpile.com/b/zPIKrB/TCQq)[10.1111/j.1651-2227.2010.01714.x](http://dx.doi.org/10.1111/j.1651-2227.2010.01714.x)

42. [Calvo C, García-García ML, Pozo F, Carballo D, Martínez-Monteserín E, Casas I. Infections and coinfections by respiratory human bocavirus during eight seasons in hospitalized children. J Med Virol. 2016;88: 2052–2058. doi:](http://paperpile.com/b/zPIKrB/PLZ0)[10.1002/jmv.24562](http://dx.doi.org/10.1002/jmv.24562)

43. [Cattoir L, Vankeerberghen A, Boel A, Van Vaerenbergh K, De Beenhouwer H. Epidemiology of RSV and hMPV in Belgium: a 10-year follow-up. Acta Clin Belg. 2018;74: 229–235. doi:](http://paperpile.com/b/zPIKrB/fNot)[10.1080/17843286.2018.1492509](http://dx.doi.org/10.1080/17843286.2018.1492509)

44. [Centers for Disease Control and Prevention (CDC). Respiratory syncytial virus--United States, July 2007-June 2011. MMWR Morb Mortal Wkly Rep. 2011;60: 1203–1206. Available:](http://paperpile.com/b/zPIKrB/u2aTv) <https://www.ncbi.nlm.nih.gov/pubmed/21900874>

45. [Chan PK, Sung RY, Fung KS, Hui M, Chik KW, Adeyemi-Doro FA, et al. Epidemiology of respiratory syncytial virus infection among paediatric patients in Hong Kong: seasonality and disease impact. Epidemiol Infect. 1999;123: 257–262. doi:](http://paperpile.com/b/zPIKrB/o9nC)[10.1017/s0950268899002824](http://dx.doi.org/10.1017/s0950268899002824)

46. [Chan PWK, Chew FT, Tan TN, Chua KB, Hooi PS. Seasonal variation in respiratory syncytial virus chest infection in the tropics. Pediatr Pulmonol. 2002;34: 47–51. doi:](http://paperpile.com/b/zPIKrB/qofI)[10.1002/ppul.10095](http://dx.doi.org/10.1002/ppul.10095)

47. [Chan PKS, Tam WWS, Lee TC, Hon KL, Lee N, Chan MCW, et al. Hospitalization Incidence, Mortality, and Seasonality of Common Respiratory Viruses Over a Period of 15 Years in a Developed Subtropical City. Medicine . 2015;94: e2024. doi:](http://paperpile.com/b/zPIKrB/sdUN2)[10.1097/MD.0000000000002024](http://dx.doi.org/10.1097/MD.0000000000002024)

48. [Chen Z, Zhu Y, Wang Y, Zhou W, Yan Y, Zhu C, et al. Association of meteorological factors with childhood viral acute respiratory infections in subtropical China: an analysis over 11 years. Arch Virol. 2013;159: 631–639. doi:](http://paperpile.com/b/zPIKrB/A33W)[10.1007/s00705-013-1863-8](http://dx.doi.org/10.1007/s00705-013-1863-8)

49. [Chew FT, Doraisingham S, Ling AE, Kumarasinghe G, Lee BW. Seasonal trends of viral respiratory tract infections in the tropics. Epidemiol Infect. 1998;121: 121–128. doi:](http://paperpile.com/b/zPIKrB/bzzz)[10.1017/s0950268898008905](http://dx.doi.org/10.1017/s0950268898008905)

50. [Chi H, Chang I-S, Tsai F-Y, Huang L-M, Shao P-L, Chiu N-C, et al. Epidemiological study of hospitalization associated with respiratory syncytial virus infection in Taiwanese children between 2004 and 2007. J Formos Med Assoc. 2011;110: 388–396. doi:](http://paperpile.com/b/zPIKrB/osdms)[10.1016/S0929-6646(11)60057-0](http://dx.doi.org/10.1016/S0929-6646(11)60057-0)

51. [Chittaganpitch M, Waicharoen S, Yingyong T, Praphasiri P, Sangkitporn S, Olsen SJ, et al. Viral etiologies of influenza-like illness and severe acute respiratory infections in Thailand. Influenza Other Respi Viruses. 2018;12: 482–489. doi:](http://paperpile.com/b/zPIKrB/RFay)[10.1111/irv.12554](http://dx.doi.org/10.1111/irv.12554)

52. [Choudhary ML, Anand SP, Wadhwa BS, Chadha MS. Genetic variability of human respiratory syncytial virus in Pune, Western India. Infect Genet Evol. 2013;20: 369–377. doi:](http://paperpile.com/b/zPIKrB/H1R9)[10.1016/j.meegid.2013.09.025](http://dx.doi.org/10.1016/j.meegid.2013.09.025)

53. [Chu F-L, Li C, Chen L, Dong B, Qiu Y, Liu Y. Respiratory viruses among pediatric inpatients with acute lower respiratory tract infections in Jinan, China, 2016-2019. J Med Virol. 2022;94: 4319–4328. doi:](http://paperpile.com/b/zPIKrB/qC9rq)[10.1002/jmv.27875](http://dx.doi.org/10.1002/jmv.27875)

54. [Cui G, Zhu R, Qian Y, Deng J, Zhao L, Sun Y, et al. Genetic variation in attachment glycoprotein genes of human respiratory syncytial virus subgroups a and B in children in recent five consecutive years. PLoS One. 2013;8: e75020. doi:](http://paperpile.com/b/zPIKrB/IMnZ)[10.1371/journal.pone.0075020](http://dx.doi.org/10.1371/journal.pone.0075020)

55. [Cui D, Feng L, Chen Y, Lai S, Zhang Z, Yu F, et al. Clinical and Epidemiologic Characteristics of Hospitalized Patients with Laboratory-Confirmed Respiratory Syncytial Virus Infection in Eastern China between 2009 and 2013: A Retrospective Study. PLoS One. 2016;11: e0165437. doi:](http://paperpile.com/b/zPIKrB/mcfA)[10.1371/journal.pone.0165437](http://dx.doi.org/10.1371/journal.pone.0165437)

56. [Darniot M, Pitoiset C, Millière L, Aho-Glélé LS, Florentin E, Bour J-B, et al. Different meteorological parameters influence metapneumovirus and respiratory syncytial virus activity. J Clin Virol. 2018;104: 77–82. doi:](http://paperpile.com/b/zPIKrB/Jb43)[10.1016/j.jcv.2018.05.002](http://dx.doi.org/10.1016/j.jcv.2018.05.002)

57. [De Conto F, Conversano F, Medici MC, Ferraglia F, Pinardi F, Arcangeletti MC, et al. Epidemiology of human respiratory viruses in children with acute respiratory tract infection in a 3-year hospital-based survey in Northern Italy. Diagn Microbiol Infect Dis. 2019;94: 260–267. doi:](http://paperpile.com/b/zPIKrB/vNcVL)[10.1016/j.diagmicrobio.2019.01.008](http://dx.doi.org/10.1016/j.diagmicrobio.2019.01.008)

58. [De Silva LM, Hanlon MG. Respiratory syncytial virus: a report of a 5-year study at a children’s hospital. J Med Virol. 1986;19: 299–305. doi:](http://paperpile.com/b/zPIKrB/zsbw)[10.1002/jmv.1890190402](http://dx.doi.org/10.1002/jmv.1890190402)

59. [Dearden CX, Jeevarathnum AC, Havinga J, Green RJ. The epidemiology of respiratory syncytial virus: A retrospective review from Steve Biko Academic Hospital 2013 - 2016. Afr J Thorac Crit Care Med. 2018;24. doi:](http://paperpile.com/b/zPIKrB/kuOZ)[10.7196/AJTCCM.2017.v24i1.163](http://dx.doi.org/10.7196/AJTCCM.2017.v24i1.163)

60. [Do AHL, van Doorn HR, Nghiem MN, Bryant JE, Hoang THT, Do QH, et al. Viral etiologies of acute respiratory infections among hospitalized Vietnamese children in Ho Chi Minh City, 2004-2008. PLoS One. 2011;6: e18176. doi:](http://paperpile.com/b/zPIKrB/tFOs)[10.1371/journal.pone.0018176](http://dx.doi.org/10.1371/journal.pone.0018176)

61. [Duppenthaler A, Gorgievski-Hrisoho M, Frey U, Aebi C. Two-year periodicity of respiratory syncytial virus epidemics in Switzerland. Infection. 2003;31: 75–80. doi:](http://paperpile.com/b/zPIKrB/CpdqX)[10.1007/s15010-002-3124-8](http://dx.doi.org/10.1007/s15010-002-3124-8)

62. [Eidelman AI, Megged O, Feldman R, Toker O. The burden of respiratory syncytial virus bronchiolitis on a pediatric inpatient service. Isr Med Assoc J. 2009;11: 533–536. Available:](http://paperpile.com/b/zPIKrB/d1TO) <https://www.ncbi.nlm.nih.gov/pubmed/19960846>

63. [Eriksson M, Bennet R, Rotzén-Ostlund M, von Sydow M, Wirgart BZ. Population-based rates of severe respiratory syncytial virus infection in children with and without risk factors, and outcome in a tertiary care setting. Acta Paediatr. 2002;91: 593–598. doi:](http://paperpile.com/b/zPIKrB/j8Al7)[10.1080/080352502753711740](http://dx.doi.org/10.1080/080352502753711740)

64. [Feng L, Li Z, Zhao S, Nair H, Lai S, Xu W, et al. Viral etiologies of hospitalized acute lower respiratory infection patients in China, 2009-2013. PLoS One. 2014;9: e99419. doi:](http://paperpile.com/b/zPIKrB/PY7d)[10.1371/journal.pone.0099419](http://dx.doi.org/10.1371/journal.pone.0099419)

65. [Fergie J, Purcell K. Respiratory syncytial virus laboratory surveillance and hospitalization trends in South Texas. Pediatr Infect Dis J. 2007;26: S51–4. doi:](http://paperpile.com/b/zPIKrB/o3kmc)[10.1097/INF.0b013e318157daae](http://dx.doi.org/10.1097/INF.0b013e318157daae)

66. [Ferrero F, Torres F, Abrutzky R, Ossorio MF, Marcos A, Ferrario C, et al. Seasonality of respiratory syncytial virus in Buenos Aires. Relationship with global climate change. Arch Argent Pediatr. 2015;114: 52–55. doi:](http://paperpile.com/b/zPIKrB/YkTtU)[10.5546/aap.2016.eng.52](http://dx.doi.org/10.5546/aap.2016.eng.52)

67. [Fjaerli H-O, Farstad T, Bratlid D. Hospitalisations for respiratory syncytial virus bronchiolitis in Akershus, Norway, 1993-2000: a population-based retrospective study. BMC Pediatr. 2004;4: 25. doi:](http://paperpile.com/b/zPIKrB/NZeh)[10.1186/1471-2431-4-25](http://dx.doi.org/10.1186/1471-2431-4-25)

68. [Fleming DM, Cross KW. Respiratory syncytial virus or influenza? Lancet. 1993;342: 1507–1510. doi:](http://paperpile.com/b/zPIKrB/4ENS)[10.1016/s0140-6736(05)80082-0](http://dx.doi.org/10.1016/s0140-6736(05)80082-0)

69. [Fleming DM, Pannell RS, Cross KW. Mortality in children from influenza and respiratory syncytial virus. J Epidemiol Community Health. 2005;59: 586–590. doi:](http://paperpile.com/b/zPIKrB/kKmQz)[10.1136/jech.2004.026450](http://dx.doi.org/10.1136/jech.2004.026450)

70. [Freitas FT de M. Sentinel surveillance of influenza and other respiratory viruses, Brazil, 2000-2010. Braz J Infect Dis. 2013;17: 62–68. doi:](http://paperpile.com/b/zPIKrB/tQMa)[10.1016/j.bjid.2012.09.001](http://dx.doi.org/10.1016/j.bjid.2012.09.001)

71. [Fry AM, Curns AT, Harbour K, Hutwagner L, Holman RC, Anderson LJ. Seasonal Trends of Human Parainfluenza Viral Infections: United States, 1990–2004. Clin Infect Dis. 2006;43: 1016–1022. doi:](http://paperpile.com/b/zPIKrB/rMNIq)[10.1086/507638](http://dx.doi.org/10.1086/507638)

72. [García-Arroyo L, Prim N, Del Cuerpo M, Marín P, Roig MC, Esteban M, et al. Prevalence and seasonality of viral respiratory infections in a temperate climate region: A 24-year study (1997-2020). Influenza Other Respi Viruses. 2022;16: 756–766. doi:](http://paperpile.com/b/zPIKrB/WKCsT)[10.1111/irv.12972](http://dx.doi.org/10.1111/irv.12972)

73. [Gentile A, Lucion MF, Juarez MDV, Areso MS, Bakir J, Viegas M, et al. Burden of Respiratory Syncytial Virus Disease and Mortality Risk Factors in Argentina: 18 Years of Active Surveillance in a Children’s Hospital. Pediatr Infect Dis J. 2019;38: 589–594. doi:](http://paperpile.com/b/zPIKrB/23GWJ)[10.1097/INF.0000000000002271](http://dx.doi.org/10.1097/INF.0000000000002271)

74. [Gil-Prieto R, Gonzalez-Escalada A, Marín-García P, Gallardo-Pino C, Gil-de-Miguel A. Respiratory Syncytial Virus Bronchiolitis in Children up to 5 Years of Age in Spain: Epidemiology and Comorbidities: An Observational Study. Medicine . 2015;94: e831. doi:](http://paperpile.com/b/zPIKrB/LQaL)[10.1097/MD.0000000000000831](http://dx.doi.org/10.1097/MD.0000000000000831)

75. [Glatman-Freedman A, Kaufman Z, Applbaum Y, Dichtiar R, Steiman A, Gordon E-S, et al. Respiratory Syncytial Virus hospitalization burden: a nation-wide population-based analysis, 2000-2017. J Infect. 2020;81: 297–303. doi:](http://paperpile.com/b/zPIKrB/nwDvF)[10.1016/j.jinf.2020.05.078](http://dx.doi.org/10.1016/j.jinf.2020.05.078)

76. [Goddard NL, Cooke MC, Gupta RK, Nguyen-Van-Tam JS. Timing of monoclonal antibody for seasonal RSV prophylaxis in the United Kingdom. Epidemiol Infect. 2006;135: 159–162. doi:](http://paperpile.com/b/zPIKrB/XvW5a)[10.1017/S0950268806006601](http://dx.doi.org/10.1017/S0950268806006601)

77. [Grilc E, Prosenc Trilar K, Lajovic J, Sočan M. Determining the seasonality of respiratory syncytial virus in Slovenia. Influenza Other Respi Viruses. 2020;15: 56–63. doi:](http://paperpile.com/b/zPIKrB/1iYBm)[10.1111/irv.12779](http://dx.doi.org/10.1111/irv.12779)

78. [Gunell M, Antikainen P, Porjo N, Irjala K, Vakkila J, Hotakainen K, et al. Comprehensive real-time epidemiological data from respiratory infections in Finland between 2010 and 2014 obtained from an automated and multianalyte mariPOC® respiratory pathogen test. Eur J Clin Microbiol Infect Dis. 2016;35: 405–413. doi:](http://paperpile.com/b/zPIKrB/1N0Q)[10.1007/s10096-015-2553-0](http://dx.doi.org/10.1007/s10096-015-2553-0)

79. [Halasa N, Williams J, Faouri S, Shehabi A, Vermund SH, Wang L, et al. Natural history and epidemiology of respiratory syncytial virus infection in the Middle East: Hospital surveillance for children under age two in Jordan. Vaccine. 2015;33: 6479–6487. doi:](http://paperpile.com/b/zPIKrB/JjTJ)[10.1016/j.vaccine.2015.08.048](http://dx.doi.org/10.1016/j.vaccine.2015.08.048)

80. [Halstead DC, Jenkins SG. Continuous non-seasonal epidemic of respiratory syncytial virus infection in the southeast United States. South Med J. 1998;91: 433–436. doi:](http://paperpile.com/b/zPIKrB/Raleq)[10.1097/00007611-199805000-00004](http://dx.doi.org/10.1097/00007611-199805000-00004)

81. [Hampp C, Asal N, Lipowski E, Kauf T, Schneider E, Kubilis P, et al. Validity of laboratory-based surveillance for detection of respiratory syncytial virus seasons. Am J Epidemiol. 2013;177: 841–851. doi:](http://paperpile.com/b/zPIKrB/7vWjg)[10.1093/aje/kws304](http://dx.doi.org/10.1093/aje/kws304)

82. [Haynes AK, Manangan AP, Iwane MK, Sturm-Ramirez K, Homaira N, Brooks WA, et al. Respiratory syncytial virus circulation in seven countries with Global Disease Detection Regional Centers. J Infect Dis. 2013;208 Suppl 3: S246–54. doi:](http://paperpile.com/b/zPIKrB/GMmUc)[10.1093/infdis/jit515](http://dx.doi.org/10.1093/infdis/jit515)

83. [Haynes AK, Fowlkes AL, Schneider E, Mutuc JD, Armstrong GL, Gerber SI. Human Metapneumovirus Circulation in the United States, 2008 to 2014. Pediatrics. 2016;137. doi:](http://paperpile.com/b/zPIKrB/HrPoU)[10.1542/peds.2015-2927](http://dx.doi.org/10.1542/peds.2015-2927)

84. [He Y, Lin G-Y, Wang Q, Cai X-Y, Zhang Y-H, Lin C-X, et al. A 3-year prospective study of the epidemiology of acute respiratory viral infections in hospitalized children in Shenzhen, China. Influenza Other Respi Viruses. 2014;8: 443–451. doi:](http://paperpile.com/b/zPIKrB/m7VoW)[10.1111/irv.12257](http://dx.doi.org/10.1111/irv.12257)

85. [Hendaus MA, Alhammadi AH, Chandra P, Muneer E, Khalifa MS. Identifying agents triggering bronchiolitis in the State of Qatar. Int J Gen Med. 2018;11: 143–149. doi:](http://paperpile.com/b/zPIKrB/Lvqn)[10.2147/IJGM.S154424](http://dx.doi.org/10.2147/IJGM.S154424)

86. [Hervás D, Reina J, Hervás JA. Meteorologic conditions and respiratory syncytial virus activity. Pediatr Infect Dis J. 2012;31: e176–81. doi:](http://paperpile.com/b/zPIKrB/e4Bx)[10.1097/INF.0b013e31825cef14](http://dx.doi.org/10.1097/INF.0b013e31825cef14)

87. [Hibino A, Saito R, Taniguchi K, Zaraket H, Shobugawa Y, Matsui T, et al. Molecular epidemiology of human respiratory syncytial virus among children in Japan during three seasons and hospitalization risk of genotype ON1. PLoS One. 2018;13: e0192085. doi:](http://paperpile.com/b/zPIKrB/Ii5N)[10.1371/journal.pone.0192085](http://dx.doi.org/10.1371/journal.pone.0192085)

88. [Hirsh S, Hindiyeh M, Kolet L, Regev L, Sherbany H, Yaary K, et al. Epidemiological changes of respiratory syncytial virus (RSV) infections in Israel. PLoS One. 2014;9: e90515. doi:](http://paperpile.com/b/zPIKrB/CQ96)[10.1371/journal.pone.0090515](http://dx.doi.org/10.1371/journal.pone.0090515)

89. [Hogan AB, Anderssen RS, Davis S, Moore HC, Lim FJ, Fathima P, et al. Time series analysis of RSV and bronchiolitis seasonality in temperate and tropical Western Australia. Epidemics. 2016;16: 49–55. doi:](http://paperpile.com/b/zPIKrB/VsIIl)[10.1016/j.epidem.2016.05.001](http://dx.doi.org/10.1016/j.epidem.2016.05.001)

90. [McMorrow ML, Tempia S, Walaza S, Treurnicht FK, Moyes J, Cohen AL, et al. The Role of Human Immunodeficiency Virus in Influenza- and Respiratory Syncytial Virus-associated Hospitalizations in South African Children, 2011-2016. Clin Infect Dis. 2019;68: 773–780. doi:](http://paperpile.com/b/zPIKrB/ktSc)[10.1093/cid/ciy532](http://dx.doi.org/10.1093/cid/ciy532)

91. [Horton KC, Dueger EL, Kandeel A, Abdallat M, El-Kholy A, Al-Awaidy S, et al. Viral etiology, seasonality and severity of hospitalized patients with severe acute respiratory infections in the Eastern Mediterranean Region, 2007-2014. PLoS One. 2017;12: e0180954. doi:](http://paperpile.com/b/zPIKrB/T8rb)[10.1371/journal.pone.0180954](http://dx.doi.org/10.1371/journal.pone.0180954)

92. [Houspie L, Lemey P, Keyaerts E, Reijmen E, Vergote V, Vankeerberghen A, et al. Circulation of HRSV in Belgium: from multiple genotype circulation to prolonged circulation of predominant genotypes. PLoS One. 2013;8: e60416. doi:](http://paperpile.com/b/zPIKrB/hReV)[10.1371/journal.pone.0060416](http://dx.doi.org/10.1371/journal.pone.0060416)

93. [Hsu C-H, Lin C-Y, Chi H, Chang J-H, Hung H-Y, Kao H-A, et al. Prolonged seasonality of respiratory syncytial virus infection among preterm infants in a subtropical climate. PLoS One. 2014;9: e110166. doi:](http://paperpile.com/b/zPIKrB/I3rwW)[10.1371/journal.pone.0110166](http://dx.doi.org/10.1371/journal.pone.0110166)

94. [Hu P, Zheng T, Chen J, Zhou T, Chen Y, Xu X, et al. Alternate circulation and genetic variation of human respiratory syncytial virus genotypes in Chengdu, West China, 2009-2014. J Med Virol. 2016;89: 32–40. doi:](http://paperpile.com/b/zPIKrB/8SKn)[10.1002/jmv.24603](http://dx.doi.org/10.1002/jmv.24603)

95. [Huang YC, Lin TY, Chang LY, Wong KS, Ning SC. Epidemiology of respiratory syncytial virus infection among paediatric inpatients in northern Taiwan. Eur J Pediatr. 2001;160: 581–582. doi:](http://paperpile.com/b/zPIKrB/3Gde)[10.1007/s004310100803](http://dx.doi.org/10.1007/s004310100803)

96. [Huang X-B, Yuan L, Ye C-X, Zhu X, Lin C-J, Zhang D-M, et al. Epidemiological characteristics of respiratory viruses in patients with acute respiratory infections during 2009-2018 in southern China. Int J Infect Dis. 2020;98: 21–32. doi:](http://paperpile.com/b/zPIKrB/5Q3h)[10.1016/j.ijid.2020.06.051](http://dx.doi.org/10.1016/j.ijid.2020.06.051)

97. [Irmen KE, Kelleher JJ. Use of monoclonal antibodies for rapid diagnosis of respiratory viruses in a community hospital. Clin Diagn Lab Immunol. 2000;7: 396–403. doi:](http://paperpile.com/b/zPIKrB/xcSo5)[10.1128/CDLI.7.3.396-403.2000](http://dx.doi.org/10.1128/CDLI.7.3.396-403.2000)

98. [Jepsen MT, Trebbien R, Emborg HD, Krause TG, Schønning K, Voldstedlund M, et al. Incidence and seasonality of respiratory syncytial virus hospitalisations in young children in Denmark, 2010 to 2015. Euro Surveill. 2018;23. doi:](http://paperpile.com/b/zPIKrB/EaElK)[10.2807/1560-7917.ES.2018.23.3.17-00163](http://dx.doi.org/10.2807/1560-7917.ES.2018.23.3.17-00163)

99. [Jiang M-L, Xu Y-P, Wu H, Zhu R-N, Sun Y, Chen D-M, et al. Changes in endemic patterns of respiratory syncytial virus infection in pediatric patients under the pressure of nonpharmaceutical interventions for COVID-19 in Beijing, China. J Med Virol. 2023;95: e28411. doi:](http://paperpile.com/b/zPIKrB/UJyei)[10.1002/jmv.28411](http://dx.doi.org/10.1002/jmv.28411)

100. [Jin Y, Zhang R-F, Xie Z-P, Yan K-L, Gao H-C, Song J-R, et al. Newly identified respiratory viruses associated with acute lower respiratory tract infections in children in Lanzou, China, from 2006 to 2009. Clin Microbiol Infect. 2011;18: 74–80. doi:](http://paperpile.com/b/zPIKrB/9VLa)[10.1111/j.1469-0691.2011.03541.x](http://dx.doi.org/10.1111/j.1469-0691.2011.03541.x)

101. [Kaneko M, Watanabe J, Kuwahara M, Ueno E, Hida M, Kinoshita A, et al. Impact of respiratory syncytial virus infection as a cause of lower respiratory tract infection in children younger than 3 years of age in Japan. J Infect. 2002;44: 240–243. doi:](http://paperpile.com/b/zPIKrB/KPEg)[10.1053/jinf.2002.0981](http://dx.doi.org/10.1053/jinf.2002.0981)

102. [Karron RA, Singleton RJ, Bulkow L, Parkinson A, Kruse D, DeSmet I, et al. Severe respiratory syncytial virus disease in Alaska native children. RSV Alaska Study Group. J Infect Dis. 1999;180: 41–49. doi:](http://paperpile.com/b/zPIKrB/YOac)[10.1086/314841](http://dx.doi.org/10.1086/314841)

103. [Khor C-S, Sam I-C, Hooi P-S, Quek K-F, Chan Y-F. Epidemiology and seasonality of respiratory viral infections in hospitalized children in Kuala Lumpur, Malaysia: a retrospective study of 27 years. BMC Pediatr. 2012;12: 32. doi:](http://paperpile.com/b/zPIKrB/IIXB)[10.1186/1471-2431-12-32](http://dx.doi.org/10.1186/1471-2431-12-32)

104. [Korsun N, Angelova S, Trifonova I, Georgieva I, Voleva S, Tzotcheva I, et al. Viral pathogens associated with acute lower respiratory tract infections in children younger than 5 years of age in Bulgaria. Braz J Microbiol. 2018;50: 117–125. doi:](http://paperpile.com/b/zPIKrB/zVld)[10.1007/s42770-018-0033-2](http://dx.doi.org/10.1007/s42770-018-0033-2)

105. [Kyeyagalire R, Tempia S, Cohen AL, Smith AD, McAnerney JM, Dermaux-Msimang V, et al. Hospitalizations associated with influenza and respiratory syncytial virus among patients attending a network of private hospitals in South Africa, 2007-2012. BMC Infect Dis. 2014;14: 694. doi:](http://paperpile.com/b/zPIKrB/H2UB)[10.1186/s12879-014-0694-x](http://dx.doi.org/10.1186/s12879-014-0694-x)

106. [Lagacé-Wiens P, Bullard J, Cole R, Van Caeseele P. Seasonality of coronaviruses and other respiratory viruses in Canada: Implications for COVID-19. Can Commun Dis Rep. 2021;47: 132–138. doi:](http://paperpile.com/b/zPIKrB/vDVJf)[10.14745/ccdr.v47i03a02](http://dx.doi.org/10.14745/ccdr.v47i03a02)

107. [Lam TT, Tang JW, Lai FY, Zaraket H, Dbaibo G, Bialasiewicz S, et al. Comparative global epidemiology of influenza, respiratory syncytial and parainfluenza viruses, 2010–2015. J Infect. 2019;79: 373–382. doi:](http://paperpile.com/b/zPIKrB/WnPPa)[10.1016/j.jinf.2019.07.008](http://dx.doi.org/10.1016/j.jinf.2019.07.008)

108. [Leecaster M, Gesteland P, Greene T, Walton N, Gundlapalli A, Rolfs R, et al. Modeling the variations in pediatric respiratory syncytial virus seasonal epidemics. BMC Infect Dis. 2011;11: 105. doi:](http://paperpile.com/b/zPIKrB/nwSk)[10.1186/1471-2334-11-105](http://dx.doi.org/10.1186/1471-2334-11-105)

109. [Li Y, Wang X, Broberg EK, Campbell H, Nair H, European RSV Surveillance Network. Seasonality of respiratory syncytial virus and its association with meteorological factors in 13 European countries, week 40 2010 to week 39 2019. Euro Surveill. 2022;27. doi:](http://paperpile.com/b/zPIKrB/68Em7)[10.2807/1560-7917.ES.2022.27.16.2100619](http://dx.doi.org/10.2807/1560-7917.ES.2022.27.16.2100619)

110. [Light M. Respiratory syncytial virus seasonality in southeast Florida: results from three area hospitals caring for children. Pediatr Infect Dis J. 2007;26: S55–9. doi:](http://paperpile.com/b/zPIKrB/VCs9C)[10.1097/INF.0b013e318157dac1](http://dx.doi.org/10.1097/INF.0b013e318157dac1)

111. [Light M, Bauman J, Mavunda K, Malinoski F, Eggleston M. Correlation between respiratory syncytial virus (RSV) test data and hospitalization of children for RSV lower respiratory tract illness in Florida. Pediatr Infect Dis J. 2008;27: 512–518. doi:](http://paperpile.com/b/zPIKrB/XkHEI)[10.1097/INF.0b013e318168daf1](http://dx.doi.org/10.1097/INF.0b013e318168daf1)

112. [Liu J, Mu Y, Dong W, Yao F, Wang L, Yan H, et al. Genetic variation of human respiratory syncytial virus among children with fever and respiratory symptoms in Shanghai, China, from 2009 to 2012. Infect Genet Evol. 2014;27: 131–136. doi:](http://paperpile.com/b/zPIKrB/CEqg)[10.1016/j.meegid.2014.07.011](http://dx.doi.org/10.1016/j.meegid.2014.07.011)

113. [Liu P, Xu M, He L, Su L, Wang A, Fu P, et al. Epidemiology of Respiratory Pathogens in Children with Lower Respiratory Tract Infections in Shanghai, China, from 2013 to 2015. Jpn J Infect Dis. 2017;71: 39–44. doi:](http://paperpile.com/b/zPIKrB/Sq1i)[10.7883/yoken.JJID.2017.323](http://dx.doi.org/10.7883/yoken.JJID.2017.323)

114. [Liu W-K, Chen D-H, Tan W-P, Qiu S-Y, Xu D, Zhang L, et al. Paramyxoviruses respiratory syncytial virus, parainfluenza virus, and human metapneumovirus infection in pediatric hospitalized patients and climate correlation in a subtropical region of southern China: a 7-year survey. Eur J Clin Microbiol Infect Dis. 2019;38: 2355–2364. doi:](http://paperpile.com/b/zPIKrB/rm9oD)[10.1007/s10096-019-03693-x](http://dx.doi.org/10.1007/s10096-019-03693-x)

115. [Loconsole D, Centrone F, Rizzo C, Caselli D, Orlandi A, Cardinale F, et al. Out-of-Season Epidemic of Respiratory Syncytial Virus during the COVID-19 Pandemic: The High Burden of Child Hospitalization in an Academic Hospital in Southern Italy in 2021. Children. 2022;9: 848. doi:](http://paperpile.com/b/zPIKrB/1S5Pg)[10.3390/children9060848](http://dx.doi.org/10.3390/children9060848)

116. [Loh TP, Lai FYL, Tan ES, Thoon KC, Tee NWS, Cutter J, et al. Correlations between clinical illness, respiratory virus infections and climate factors in a tropical paediatric population. Epidemiol Infect. 2011;139: 1884–1894. doi:](http://paperpile.com/b/zPIKrB/yLS8)[10.1017/S0950268810002955](http://dx.doi.org/10.1017/S0950268810002955)

117. [Low YL, Wong SY, Lee EKH, Muhammed MH. Prevalence of respiratory viruses among paediatric patients in acute respiratory illnesses in Malaysia. PLoS One. 2022;17: e0265288. doi:](http://paperpile.com/b/zPIKrB/8OXnW)[10.1371/journal.pone.0265288](http://dx.doi.org/10.1371/journal.pone.0265288)

118. [Lu L, Yan Y, Yang B, Xiao Z, Feng X, Wang Y, et al. Epidemiological and clinical profiles of respiratory syncytial virus infection in hospitalized neonates in Suzhou, China. BMC Infect Dis. 2015;15: 431. doi:](http://paperpile.com/b/zPIKrB/UFDf)[10.1186/s12879-015-1155-x](http://dx.doi.org/10.1186/s12879-015-1155-x)

119. [Lumley SF, Richens N, Lees E, Cregan J, Kalimeris E, Oakley S, et al. Changes in paediatric respiratory infections at a UK teaching hospital 2016-2021; impact of the SARS-CoV-2 pandemic. J Infect. 2021;84: 40–47. doi:](http://paperpile.com/b/zPIKrB/L8WR)[10.1016/j.jinf.2021.10.022](http://dx.doi.org/10.1016/j.jinf.2021.10.022)

120. [Luo M, Gong C, Zhang Y, Wang X, Liu Y, Luo Q, et al. Comparison of infections with respiratory syncytial virus between children and adults: a multicenter surveillance from 2015 to 2019 in Beijing, China. Eur J Clin Microbiol Infect Dis. 2022;41: 1387–1397. doi:](http://paperpile.com/b/zPIKrB/RCZsO)[10.1007/s10096-022-04492-7](http://dx.doi.org/10.1007/s10096-022-04492-7)

121. [Mak GC, Wong AH, Ho WYY, Lim W. The impact of pandemic influenza A (H1N1) 2009 on the circulation of respiratory viruses 2009-2011. Influenza Other Respi Viruses. 2012;6: e6–10. doi:](http://paperpile.com/b/zPIKrB/J6R1R)[10.1111/j.1750-2659.2011.00323.x](http://dx.doi.org/10.1111/j.1750-2659.2011.00323.x)

122. [Martin AJ, Gardner PS, McQuillin J. Epidemiology of respiratory viral infection among paediatric inpatients over a six-year period in north-east England. Lancet. 1978;2: 1035–1038. doi:](http://paperpile.com/b/zPIKrB/Fz7X)[10.1016/s0140-6736(78)92351-6](http://dx.doi.org/10.1016/s0140-6736(78)92351-6)

123. [McCracken JP, Arvelo W, Ortíz J, Reyes L, Gray J, Estevez A, et al. Comparative epidemiology of human metapneumovirus- and respiratory syncytial virus-associated hospitalizations in Guatemala. Influenza Other Respi Viruses. 2014;8: 414–421. doi:](http://paperpile.com/b/zPIKrB/RKhU)[10.1111/irv.12251](http://dx.doi.org/10.1111/irv.12251)

124. [McGuiness CB, Boron ML, Saunders B, Edelman L, Kumar VR, Rabon-Stith KM. Respiratory syncytial virus surveillance in the United States, 2007-2012: results from a national surveillance system. Pediatr Infect Dis J. 2014;33: 589–594. doi:](http://paperpile.com/b/zPIKrB/aBsPn)[10.1097/INF.0000000000000257](http://dx.doi.org/10.1097/INF.0000000000000257)

125. [Meerhoff TJ, Paget JW, Kimpen JL, Schellevis F. Variation of respiratory syncytial virus and the relation with meteorological factors in different winter seasons. Pediatr Infect Dis J. 2009;28: 860–866. doi:](http://paperpile.com/b/zPIKrB/br0sv)[10.1097/INF.0b013e3181a3e949](http://dx.doi.org/10.1097/INF.0b013e3181a3e949)

126. [Meningher T, Hindiyeh M, Regev L, Sherbany H, Mendelson E, Mandelboim M. Relationships between A(H1N1)pdm09 influenza infection and infections with other respiratory viruses. Influenza Other Respi Viruses. 2014;8: 422–430. doi:](http://paperpile.com/b/zPIKrB/LCRZ)[10.1111/irv.12249](http://dx.doi.org/10.1111/irv.12249)

127. [Midgley CM, Haynes AK, Baumgardner JL, Chommanard C, Demas SW, Prill MM, et al. Determining the Seasonality of Respiratory Syncytial Virus in the United States: The Impact of Increased Molecular Testing. J Infect Dis. 2017;216: 345–355. doi:](http://paperpile.com/b/zPIKrB/LX59k)[10.1093/infdis/jix275](http://dx.doi.org/10.1093/infdis/jix275)

128. [Miller EK, Gebretsadik T, Carroll KN, Dupont WD, Mohamed YA, Morin L-L, et al. Viral etiologies of infant bronchiolitis, croup and upper respiratory illness during 4 consecutive years. Pediatr Infect Dis J. 2013;32: 950–955. doi:](http://paperpile.com/b/zPIKrB/7hOJ)[10.1097/INF.0b013e31829b7e43](http://dx.doi.org/10.1097/INF.0b013e31829b7e43)

129. [Miyama T, Iritani N, Nishio T, Ukai T, Satsuki Y, Miyata H, et al. Seasonal shift in epidemics of respiratory syncytial virus infection in Japan. Epidemiol Infect. 2021;149: e55. doi:](http://paperpile.com/b/zPIKrB/hD5ZO)[10.1017/S0950268821000340](http://dx.doi.org/10.1017/S0950268821000340)

130. [Mizuta K, Abiko C, Aoki Y, Ikeda T, Matsuzaki Y, Itagaki T, et al. Seasonal patterns of respiratory syncytial virus, influenza A virus, human metapneumovirus, and parainfluenza virus type 3 infections on the basis of virus isolation data between 2004 and 2011 in Yamagata, Japan. Jpn J Infect Dis. 2013;66: 140–145. doi:](http://paperpile.com/b/zPIKrB/UQ2i)[10.7883/yoken.66.140](http://dx.doi.org/10.7883/yoken.66.140)

131. [Mlinaric-Galinovic G, Welliver RC, Vilibic-Cavlek T, Ljubin-Sternak S, Drazenovic V, Galinovic I, et al. The biennial cycle of respiratory syncytial virus outbreaks in Croatia. Virol J. 2008;5: 18. doi:](http://paperpile.com/b/zPIKrB/otzRz)[10.1186/1743-422X-5-18](http://dx.doi.org/10.1186/1743-422X-5-18)

132. [Montgomery AS, Lustik MB, Jones MU, Horseman TS. Respiratory Viral Pathogens in Children Evaluated at Military Treatment Facilities in Oahu, Hawaii From 2014 to 2018: Seasonality and Climatic Factors. J Pediatric Infect Dis Soc. 2021;10: 517–520. doi:](http://paperpile.com/b/zPIKrB/3KyN)[10.1093/jpids/piaa131](http://dx.doi.org/10.1093/jpids/piaa131)

133. [Moore HC, de Klerk N, Richmond P, Keil AD, Lindsay K, Plant A, et al. Seasonality of respiratory viral identification varies with age and Aboriginality in metropolitan Western Australia. Pediatr Infect Dis J. 2009;28: 598–603. doi:](http://paperpile.com/b/zPIKrB/mAtYr)[10.1097/INF.0b013e318199cefd](http://dx.doi.org/10.1097/INF.0b013e318199cefd)

134. [Morley C, Grimwood K, Maloney S, Ware RS. Meteorological factors and respiratory syncytial virus seasonality in subtropical Australia. Epidemiol Infect. 2018;146: 757–762. doi:](http://paperpile.com/b/zPIKrB/5eVn)[10.1017/S0950268818000614](http://dx.doi.org/10.1017/S0950268818000614)

135. [Moura FEA, Perdigão ACB, Ribeiro JF, Florêncio CMGD, Oliveira FMS, Pereira SAR, et al. Respiratory syncytial virus epidemic periods in an equatorial city of Brazil. Influenza Other Respi Viruses. 2013;7: 1128–1135. doi:](http://paperpile.com/b/zPIKrB/yqMda)[10.1111/irv.12104](http://dx.doi.org/10.1111/irv.12104)

136. [Movva N, Suh M, Reichert H, Hintze B, Sendak MP, Wolf Z, et al. Respiratory Syncytial Virus During the COVID-19 Pandemic Compared to Historic Levels: A Retrospective Cohort Study of a Health System. J Infect Dis. 2022;226: S175–S183. doi:](http://paperpile.com/b/zPIKrB/ddPlR)[10.1093/infdis/jiac220](http://dx.doi.org/10.1093/infdis/jiac220)

137. [Mufson MA, Levine HD, Wasil RE, Mocega-Gonzalez HE, Krause HE. Epidemiology of respiratory syncytial virus infection among infants and children in Chicago. Am J Epidemiol. 1973;98: 88–95. doi:](http://paperpile.com/b/zPIKrB/ACqt)[10.1093/oxfordjournals.aje.a121542](http://dx.doi.org/10.1093/oxfordjournals.aje.a121542)

138. [Mullins JA, Lamonte AC, Bresee JS, Anderson LJ. Substantial variability in community respiratory syncytial virus season timing. Pediatr Infect Dis J. 2003;22: 857–862. doi:](http://paperpile.com/b/zPIKrB/QzfFW)[10.1097/01.inf.0000090921.21313.d3](http://dx.doi.org/10.1097/01.inf.0000090921.21313.d3)

139. [Noveroske DB, Warren JL, Pitzer VE, Weinberger DM. Local variations in the timing of RSV epidemics. BMC Infect Dis. 2016;16: 674. doi:](http://paperpile.com/b/zPIKrB/JiaW8)[10.1186/s12879-016-2004-2](http://dx.doi.org/10.1186/s12879-016-2004-2)

140. [Nyoka R, Omony J, Mwalili SM, Achia TNO, Gichangi A, Mwambi H. Effect of climate on incidence of respiratory syncytial virus infections in a refugee camp in Kenya: A non-Gaussian time-series analysis. PLoS One. 2017;12: e0178323. doi:](http://paperpile.com/b/zPIKrB/hdTg7)[10.1371/journal.pone.0178323](http://dx.doi.org/10.1371/journal.pone.0178323)

141. [O’Kelly EA, Hillary IB. Epidemiology of respiratory syncytial virus infection among infants over three winter seasons. Ir J Med Sci. 1991;160: 12–16. doi:](http://paperpile.com/b/zPIKrB/o3vf)[10.1007/BF02944725](http://dx.doi.org/10.1007/BF02944725)

142. [Obando-Pacheco P, Justicia-Grande AJ, Rivero-Calle I, Rodríguez-Tenreiro C, Sly P, Ramilo O, et al. Respiratory Syncytial Virus Seasonality: A Global Overview. J Infect Dis. 2018;217: 1356–1364. doi:](http://paperpile.com/b/zPIKrB/h4sVb)[10.1093/infdis/jiy056](http://dx.doi.org/10.1093/infdis/jiy056)

143. [Oliveira-Santos M, Santos JA, Soares J, Dias A, Quaresma M. Influence of meteorological conditions on RSV infection in Portugal. Int J Biometeorol. 2016;60: 1807–1817. doi:](http://paperpile.com/b/zPIKrB/uLso)[10.1007/s00484-016-1168-1](http://dx.doi.org/10.1007/s00484-016-1168-1)

144. [Oskarsson Y, Haraldsson A, Oddsdottir BHI, Asgeirsdottir TL, Thors V. Clinical and Socioeconomic Burden of Respiratory Syncytial Virus in Iceland. Pediatr Infect Dis J. 2022;41: 800–805. doi:](http://paperpile.com/b/zPIKrB/zY6Hw)[10.1097/INF.0000000000003640](http://dx.doi.org/10.1097/INF.0000000000003640)

145. [Paes BA, Craig C, Pigott W, Latchman A. Seasonal respiratory syncytial virus prophylaxis based on predetermined dates versus regional surveillance data. Pediatr Infect Dis J. 2013;32: e360–4. doi:](http://paperpile.com/b/zPIKrB/ChHdE)[10.1097/INF.0b013e31829479d3](http://dx.doi.org/10.1097/INF.0b013e31829479d3)

146. [Paiva TM, Ishida MA, Benega MA, Constantino CRA, Silva DBB, Santos KCO, et al. Shift in the timing of respiratory syncytial virus circulation in a subtropical megalopolis: implications for immunoprophylaxis. J Med Virol. 2012;84: 1825–1830. doi:](http://paperpile.com/b/zPIKrB/PfEIA)[10.1002/jmv.23347](http://dx.doi.org/10.1002/jmv.23347)

147. [Panozzo CA, Stockman LJ, Curns AT, Anderson LJ. Use of respiratory syncytial virus surveillance data to optimize the timing of immunoprophylaxis. Pediatrics. 2010;126: e116–23. doi:](http://paperpile.com/b/zPIKrB/6twje)[10.1542/peds.2009-3221](http://dx.doi.org/10.1542/peds.2009-3221)

148. [Paynter S, Ware RS, Sly PD, Weinstein P, Williams G. Respiratory syncytial virus seasonality in tropical Australia. Aust N Z J Public Health. 2015;39: 8–10. doi:](http://paperpile.com/b/zPIKrB/2gJR)[10.1111/1753-6405.12347](http://dx.doi.org/10.1111/1753-6405.12347)

149. [Pellegrinelli L, Galli C, Bubba L, Seiti A, Anselmi G, Primache V, et al. Respiratory syncytial virus in pediatric influenza-like illness cases in Lombardy, Northern Italy, during seven consecutive winter seasons (from 2014-2015 to 2020-2021). Influenza Other Respi Viruses. 2021. doi:](http://paperpile.com/b/zPIKrB/Ox5th)[10.1111/irv.12940](http://dx.doi.org/10.1111/irv.12940)

150. [Peterson I, Bar-Zeev N, Kennedy N, Ho A, Newberry L, SanJoaquin MA, et al. Respiratory Virus-Associated Severe Acute Respiratory Illness and Viral Clustering in Malawian Children in a Setting With a High Prevalence of HIV Infection, Malaria, and Malnutrition. J Infect Dis. 2016;214: 1700–1711. doi:](http://paperpile.com/b/zPIKrB/2IZU)[10.1093/infdis/jiw426](http://dx.doi.org/10.1093/infdis/jiw426)

151. [Pierangeli A, Trotta D, Scagnolari C, Ferreri ML, Nicolai A, Midulla F, et al. Rapid spread of the novel respiratory syncytial virus A ON1 genotype, central Italy, 2011 to 2013. Euro Surveill. 2014;19. doi:](http://paperpile.com/b/zPIKrB/5UR0)[10.2807/1560-7917.es2014.19.26.20843](http://dx.doi.org/10.2807/1560-7917.es2014.19.26.20843)

152. [Price RHM, Graham C, Ramalingam S. Association between viral seasonality and meteorological factors. Sci Rep. 2019;9: 929. doi:](http://paperpile.com/b/zPIKrB/b012)[10.1038/s41598-018-37481-y](http://dx.doi.org/10.1038/s41598-018-37481-y)

153. [Ramaekers K, Keyaerts E, Rector A, Borremans A, Beuselinck K, Lagrou K, et al. Prevalence and seasonality of six respiratory viruses during five consecutive epidemic seasons in Belgium. J Clin Virol. 2017;94: 72–78. doi:](http://paperpile.com/b/zPIKrB/eCEh)[10.1016/j.jcv.2017.07.011](http://dx.doi.org/10.1016/j.jcv.2017.07.011)

154. [Reeves RM, Hardelid P, Gilbert R, Ellis J, Zhao H, Donati M, et al. Epidemiology of laboratory-confirmed respiratory syncytial virus infection in young children in England, 2010–2014: the importance of birth month. Epidemiology & Infection. 2016;144: 2049–2056. doi:](http://paperpile.com/b/zPIKrB/3NLPe)[10.1017/S0950268816000352](http://dx.doi.org/10.1017/S0950268816000352)

155. [Reiche J, Schweiger B. Genetic variability of group A human respiratory syncytial virus strains circulating in Germany from 1998 to 2007. J Clin Microbiol. 2009;47: 1800–1810. doi:](http://paperpile.com/b/zPIKrB/lp0rv)[10.1128/JCM.02286-08](http://dx.doi.org/10.1128/JCM.02286-08)

156. [Renko M, Tapiainen T. Change in respiratory syncytial virus seasonality in Finland. Acta Paediatr. 2019;109: 202–203. doi:](http://paperpile.com/b/zPIKrB/pBuC)[10.1111/apa.14983](http://dx.doi.org/10.1111/apa.14983)

157. [Reyes M, Eriksson M, Bennet R, Hedlund K-O, Ehrnst A. Regular pattern of respiratory syncytial virus and rotavirus infections and relation to weather in Stockholm, 1984--1993. Clin Microbiol Infect. 1997;3: 640–646. doi:](http://paperpile.com/b/zPIKrB/uo77Z)[10.1111/j.1469-0691.1997.tb00471.x](http://dx.doi.org/10.1111/j.1469-0691.1997.tb00471.x)

158. [Richter J, Panayiotou C, Tryfonos C, Koptides D, Koliou M, Kalogirou N, et al. Aetiology of Acute Respiratory Tract Infections in Hospitalised Children in Cyprus. PLoS One. 2016;11: e0147041. doi:](http://paperpile.com/b/zPIKrB/NkWB)[10.1371/journal.pone.0147041](http://dx.doi.org/10.1371/journal.pone.0147041)

159. [Rose EB, Wheatley A, Langley G, Gerber S, Haynes A. Respiratory Syncytial Virus Seasonality - United States, 2014-2017. MMWR Morb Mortal Wkly Rep. 2018;67: 71–76. doi:](http://paperpile.com/b/zPIKrB/l3Uth)[10.15585/mmwr.mm6702a4](http://dx.doi.org/10.15585/mmwr.mm6702a4)

160. [Rose EB, Nyawanda BO, Munywoki PK, Murunga N, Bigogo GM, Otieno NA, et al. Respiratory syncytial virus seasonality in three epidemiological zones of Kenya. Influenza Other Respi Viruses. 2020;15: 195–201. doi:](http://paperpile.com/b/zPIKrB/i2Xbo)[10.1111/irv.12810](http://dx.doi.org/10.1111/irv.12810)

161. [Rowlinson E, Dueger E, Mansour A, Azzazy N, Mansour H, Peters L, et al. Incidence and etiology of hospitalized acute respiratory infections in the Egyptian Delta. Influenza Other Respi Viruses. 2016;11: 23–32. doi:](http://paperpile.com/b/zPIKrB/99ho)[10.1111/irv.12409](http://dx.doi.org/10.1111/irv.12409)

162. [Rząd M, Kanecki K, Lewtak K, Tyszko P, Szwejkowska M, Goryński P, et al. Human Respiratory Syncytial Virus Infections among Hospitalized Children in Poland during 2010-2020: Study Based on the National Hospital Registry. J Clin Med Res. 2022;11. doi:](http://paperpile.com/b/zPIKrB/R9I6x)[10.3390/jcm11216451](http://dx.doi.org/10.3390/jcm11216451)

163. [Sato M, Saito R, Sakai T, Sano Y, Nishikawa M, Sasaki A, et al. Molecular epidemiology of respiratory syncytial virus infections among children with acute respiratory symptoms in a community over three seasons. J Clin Microbiol. 2005;43: 36–40. doi:](http://paperpile.com/b/zPIKrB/yDbN)[10.1128/JCM.43.1.36-40.2005](http://dx.doi.org/10.1128/JCM.43.1.36-40.2005)

164. [Shobugawa Y, Takeuchi T, Hibino A, Hassan MR, Yagami R, Kondo H, et al. Occurrence of human respiratory syncytial virus in summer in Japan. Epidemiol Infect. 2016;145: 272–284. doi:](http://paperpile.com/b/zPIKrB/dlg8O)[10.1017/S095026881600220X](http://dx.doi.org/10.1017/S095026881600220X)

165. [Singleton RJ, Bruden D, Bulkow LR. Respiratory syncytial virus season and hospitalizations in the Alaskan Yukon-Kuskokwim Delta. Pediatr Infect Dis J. 2007;26: S46–50. doi:](http://paperpile.com/b/zPIKrB/VnTAx)[10.1097/INF.0b013e318157da9b](http://dx.doi.org/10.1097/INF.0b013e318157da9b)

166. [Sirimi N, Miligkos M, Koutouzi F, Petridou E, Siahanidou T, Michos A. Respiratory syncytial virus activity and climate parameters during a 12-year period. J Med Virol. 2015;88: 931–937. doi:](http://paperpile.com/b/zPIKrB/65sk)[10.1002/jmv.24430](http://dx.doi.org/10.1002/jmv.24430)

167. [Sitthikarnkha P, Uppala R, Niamsanit S, Sutra S, Thepsuthammarat K, Techasatian L, et al. Burden of Respiratory Syncytial Virus Related Acute Lower Respiratory Tract Infection in Hospitalized Thai Children: A 6-Year National Data Analysis. Children. 2022;9. doi:](http://paperpile.com/b/zPIKrB/snhh4)[10.3390/children9121990](http://dx.doi.org/10.3390/children9121990)

168. [Stockman LJ, Brooks WA, Streatfield PK, Rahman M, Goswami D, Nahar K, et al. Challenges to evaluating respiratory syncytial virus mortality in Bangladesh, 2004-2008. PLoS One. 2013;8: e53857. doi:](http://paperpile.com/b/zPIKrB/9Yq61)[10.1371/journal.pone.0053857](http://dx.doi.org/10.1371/journal.pone.0053857)

169. [Straliotto SM, Nestor SM, Siqueira MM. Respiratory syncytial virus groups A and B in Porto Alegre, Brazil, from 1990 to 1995 and 1998. Mem Inst Oswaldo Cruz. 2001;96: 155–158. doi:](http://paperpile.com/b/zPIKrB/KWRS)[10.1590/s0074-02762001000200003](http://dx.doi.org/10.1590/s0074-02762001000200003)

170. [Straliotto SM, Siqueira MM, Muller RL, Fischer GB, Cunha MLT, Nestor SM. Viral etiology of acute respiratory infections among children in Porto Alegre, RS, Brazil. Rev Soc Bras Med Trop. 2002;35: 283–291. doi:](http://paperpile.com/b/zPIKrB/OHWs)[10.1590/s0037-86822002000400002](http://dx.doi.org/10.1590/s0037-86822002000400002)

171. [Sundell N, Andersson L-M, Brittain-Long R, Lindh M, Westin J. A four year seasonal survey of the relationship between outdoor climate and epidemiology of viral respiratory tract infections in a temperate climate. J Clin Virol. 2016;84: 59–63. doi:](http://paperpile.com/b/zPIKrB/f3Cy)[10.1016/j.jcv.2016.10.005](http://dx.doi.org/10.1016/j.jcv.2016.10.005)

172. [Sutmöller F, Ferro ZP, Asensi MD, Ferreira V, Mazzei IS, Cunha BL. Etiology of acute respiratory tract infections among children in a combined community and hospital study in Rio de Janeiro. Clin Infect Dis. 1995;20: 854–860. doi:](http://paperpile.com/b/zPIKrB/f9vki)[10.1093/clinids/20.4.854](http://dx.doi.org/10.1093/clinids/20.4.854)

173. [Tabatabai J, Ihling CM, Rehbein RM, Schnee SV, Hoos J, Pfeil J, et al. Molecular epidemiology of respiratory syncytial virus in hospitalised children in Heidelberg, Southern Germany, 2014-2017. Infect Genet Evol. 2022;98: 105209. doi:](http://paperpile.com/b/zPIKrB/A1UTM)[10.1016/j.meegid.2022.105209](http://dx.doi.org/10.1016/j.meegid.2022.105209)

174. [Tan KWJ, Yung CF, Maiwald M, Saffari SE, Thoon KC, Chong CY. Respiratory viral infections in hospitalised paediatric patients in the tropics. J Paediatr Child Health. 2020;57: 559–565. doi:](http://paperpile.com/b/zPIKrB/vTj3)[10.1111/jpc.15267](http://dx.doi.org/10.1111/jpc.15267)

175. [Tang JW, Lai FYL, Wong F, Hon KLE. Incidence of common respiratory viral infections related to climate factors in hospitalized children in Hong Kong. Epidemiol Infect. 2009;138: 226–235. doi:](http://paperpile.com/b/zPIKrB/wB8zW)[10.1017/S0950268809990410](http://dx.doi.org/10.1017/S0950268809990410)

176. [Terletskaia-Ladwig E, Enders G, Schalasta G, Enders M. Defining the timing of respiratory syncytial virus (RSV) outbreaks: an epidemiological study. BMC Infect Dis. 2005;5: 20. doi:](http://paperpile.com/b/zPIKrB/KGT1J)[10.1186/1471-2334-5-20](http://dx.doi.org/10.1186/1471-2334-5-20)

177. [Thomas E, Margach MJ, Orvell C, Morrison B, Wilson E. Respiratory syncytial virus subgroup B dominance during one winter season between 1987 and 1992 in Vancouver, Canada. J Clin Microbiol. 1994;32: 238–242. doi:](http://paperpile.com/b/zPIKrB/vvvR)[10.1128/jcm.32.1.238-242.1994](http://dx.doi.org/10.1128/jcm.32.1.238-242.1994)

178. [Thongpan I, Vongpunsawad S, Poovorawan Y. Respiratory syncytial virus infection trend is associated with meteorological factors. Sci Rep. 2020;10: 10931. doi:](http://paperpile.com/b/zPIKrB/tlHj)[10.1038/s41598-020-67969-5](http://dx.doi.org/10.1038/s41598-020-67969-5)

179. [Thwaites R, Buchan S, Fullarton J, Morris C, Grubb E, Rodgers-Gray B, et al. Clinical burden of severe respiratory syncytial virus infection during the first 2 years of life in children born between 2000 and 2011 in Scotland. Eur J Pediatr. 2020;179: 791–799. doi:](http://paperpile.com/b/zPIKrB/DTr5)[10.1007/s00431-019-03564-9](http://dx.doi.org/10.1007/s00431-019-03564-9)

180. [Tsolia MN, Kafetzis D, Danelatou K, Astral H, Kallergi K, Spyridis P, et al. Epidemiology of respiratory syncytial virus bronchiolitis in hospitalized infants in Greece. Eur J Epidemiol. 2003;18: 55–61. doi:](http://paperpile.com/b/zPIKrB/TMXA)[10.1023/a:1022556215190](http://dx.doi.org/10.1023/a:1022556215190)

181. [Turner C, Turner P, Cararra V, Eh Lwe N, Watthanaworawit W, Day NP, et al. A high burden of respiratory syncytial virus associated pneumonia in children less than two years of age in a South East Asian refugee population. PLoS One. 2012;7: e50100. doi:](http://paperpile.com/b/zPIKrB/Y1v0)[10.1371/journal.pone.0050100](http://dx.doi.org/10.1371/journal.pone.0050100)

182. [Učakar V, Sočan M, Trilar KP. The impact of influenza and respiratory syncytial virus on hospitalizations for lower respiratory tract infections in young children: Slovenia, 2006-2011. Influenza Other Respi Viruses. 2013;7: 1093–1102. doi:](http://paperpile.com/b/zPIKrB/dkxqZ)[10.1111/irv.12134](http://dx.doi.org/10.1111/irv.12134)

183. [Valley-Omar Z, Tempia S, Hellferscee O, Walaza S, Variava E, Dawood H, et al. Human respiratory syncytial virus diversity and epidemiology among patients hospitalized with severe respiratory illness in South Africa, 2012-2015. Influenza Other Respi Viruses. 2021;16: 222–235. doi:](http://paperpile.com/b/zPIKrB/1Q5Yl)[10.1111/irv.12905](http://dx.doi.org/10.1111/irv.12905)

184. [van der Sande MAB, Goetghebuer T, Sanneh M, Whittle HC, Weber MW. Seasonal variation in respiratory syncytial virus epidemics in the Gambia, West Africa. Pediatr Infect Dis J. 2004;23: 73–74. doi:](http://paperpile.com/b/zPIKrB/YMZz)[10.1097/01.inf.0000105183.12781.06](http://dx.doi.org/10.1097/01.inf.0000105183.12781.06)

185. [van Summeren J, Meijer A, Aspelund G, Casalegno JS, Erna G, Hoang U, et al. Low levels of respiratory syncytial virus activity in Europe during the 2020/21 season: what can we expect in the coming summer and autumn/winter? Euro Surveill. 2021;26. doi:](http://paperpile.com/b/zPIKrB/HoDc)[10.2807/1560-7917.ES.2021.26.29.2100639](http://dx.doi.org/10.2807/1560-7917.ES.2021.26.29.2100639)

186. [Vandini S, Corvaglia L, Alessandroni R, Aquilano G, Marsico C, Spinelli M, et al. Respiratory syncytial virus infection in infants and correlation with meteorological factors and air pollutants. Ital J Pediatr. 2013;39: 1. doi:](http://paperpile.com/b/zPIKrB/4NIp)[10.1186/1824-7288-39-1](http://dx.doi.org/10.1186/1824-7288-39-1)

187. [Verani JR, McCracken J, Arvelo W, Estevez A, Lopez MR, Reyes L, et al. Surveillance for hospitalized acute respiratory infection in Guatemala. PLoS One. 2013;8: e83600. doi:](http://paperpile.com/b/zPIKrB/el7n)[10.1371/journal.pone.0083600](http://dx.doi.org/10.1371/journal.pone.0083600)

188. [Viegas M, Barrero PR, Maffey AF, Mistchenko AS. Respiratory viruses seasonality in children under five years of age in Buenos Aires, ArgentinaA five-year analysis. J Infect. 2004;49: 222–228. doi:](http://paperpile.com/b/zPIKrB/OHDD)[10.1016/j.jinf.2003.10.006](http://dx.doi.org/10.1016/j.jinf.2003.10.006)

189. [Viguria N, Martínez-Baz I, Moreno-Galarraga L, Sierrasesúmaga L, Salcedo B, Castilla J. Respiratory syncytial virus hospitalization in children in northern Spain. PLoS One. 2018;13: e0206474. doi:](http://paperpile.com/b/zPIKrB/vwxwK)[10.1371/journal.pone.0206474](http://dx.doi.org/10.1371/journal.pone.0206474)

190. [Vila J, Lera E, Andrés C, Piñana M, Rello-Saltor V, Tobeña-Rué M, et al. The burden of non-SARS-CoV2 viral lower respiratory tract infections in hospitalized children in Barcelona (Spain): A long-term, clinical, epidemiologic and economic study. Influenza Other Respi Viruses. 2022;17. doi:](http://paperpile.com/b/zPIKrB/QTdsL)[10.1111/irv.13085](http://dx.doi.org/10.1111/irv.13085)

191. [Vos LM, Teirlinck AC, Lozano JE, Vega T, Donker GA, Hoepelman AI, et al. Use of the moving epidemic method (MEM) to assess national surveillance data for respiratory syncytial virus (RSV) in the Netherlands, 2005 to 2017. Euro Surveill. 2019;24. doi:](http://paperpile.com/b/zPIKrB/6BC5G)[10.2807/1560-7917.ES.2019.24.20.1800469](http://dx.doi.org/10.2807/1560-7917.ES.2019.24.20.1800469)

192. [Wagatsuma K, Koolhof IS, Shobugawa Y, Saito R. Shifts in the epidemic season of human respiratory syncytial virus associated with inbound overseas travelers and meteorological conditions in Japan, 2014-2017: An ecological study. PLoS One. 2021;16: e0248932. doi:](http://paperpile.com/b/zPIKrB/Yt6m7)[10.1371/journal.pone.0248932](http://dx.doi.org/10.1371/journal.pone.0248932)

193. [Wahab AA, Dawod ST, Raman HM. Clinical characteristics of respiratory syncytial virus infection in hospitalized healthy infants and young children in Qatar. J Trop Pediatr. 2001;47: 363–366. doi:](http://paperpile.com/b/zPIKrB/g9zZ)[10.1093/tropej/47.6.363](http://dx.doi.org/10.1093/tropej/47.6.363)

194. [Wang L, Davis PB, Berger NA, Kaelber DC, Volkow N, Xu R. Disruption in seasonality, patient characteristics and disparities of respiratory syncytial virus infection among young children in the US during and before the COVID-19 pandemic: 2010-2022. medRxiv. 2022. doi:](http://paperpile.com/b/zPIKrB/an6hH)[10.1101/2022.11.29.22282887](http://dx.doi.org/10.1101/2022.11.29.22282887)

195. [Weber MW, Dackour R, Usen S, Schneider G, Adegbola RA, Cane P, et al. The clinical spectrum of respiratory syncytial virus disease in The Gambia. The Pediatric Infectious Disease Journal. 1998. pp. 224–230. doi:](http://paperpile.com/b/zPIKrB/4DDq)[10.1097/00006454-199803000-00010](http://dx.doi.org/10.1097/00006454-199803000-00010)

196. [Weigl JA, Puppe W, Gröndahl B, Schmitt HJ. Epidemiological investigation of nine respiratory pathogens in hospitalized children in Germany using multiplex reverse-transcriptase polymerase chain reaction. Eur J Clin Microbiol Infect Dis. 2000;19: 336–343. doi:](http://paperpile.com/b/zPIKrB/n9ew)[10.1007/s100960050490](http://dx.doi.org/10.1007/s100960050490)

197. [Weigl JAI, Puppe W, Schmitt HJ. Seasonality of respiratory syncytial virus-positive hospitalizations in children in Kiel, Germany, over a 7-year period. Infection. 2002;30: 186–192. doi:](http://paperpile.com/b/zPIKrB/7fCuW)[10.1007/s15010-002-2159-1](http://dx.doi.org/10.1007/s15010-002-2159-1)

198. [Weissenbacher M, Carballal G, Avila M, Salomón H, Harisiadi J, Catalano M, et al. Etiologic and clinical evaluation of acute lower respiratory tract infections in young Argentinian children: an overview. Rev Infect Dis. 1990;12 Suppl 8: S889–98. doi:](http://paperpile.com/b/zPIKrB/xcs0)[10.1093/clinids/12.supplement_8.s889](http://dx.doi.org/10.1093/clinids/12.supplement_8.s889)

199. [Wilfret DA, Baker BT, Palavecino E, Moran C, Benjamin DK Jr. Epidemiology of respiratory syncytial virus in various regions within North Carolina during multiple seasons. N C Med J. 2008;69: 447–452. Available:](http://paperpile.com/b/zPIKrB/pdOs) <https://www.ncbi.nlm.nih.gov/pubmed/19256181>

200. [Winter GF, Hallam NF, Hargreaves FD, Molyneaux PJ, Burns SM, Inglis JM. Respiratory viruses in a hospitalized paediatric population in Edinburgh 1985-1994. J Infect. 1996;33: 207–211. doi:](http://paperpile.com/b/zPIKrB/rzNg)[10.1016/s0163-4453(96)92297-5](http://dx.doi.org/10.1016/s0163-4453(96)92297-5)

201. [Wrotek A, Czajkowska M, Jackowska T. Seasonality of Respiratory Syncytial Virus Hospitalization. Adv Exp Med Biol. 2020;1279: 93–100. doi:](http://paperpile.com/b/zPIKrB/u8hZN)[10.1007/5584_2020_503](http://dx.doi.org/10.1007/5584_2020_503)

202. [Yamagami H, Kimura H, Hashimoto T, Kusakawa I, Kusuda S. Detection of the Onset of the Epidemic Period of Respiratory Syncytial Virus Infection in Japan. Front Public Health. 2019;7: 39. doi:](http://paperpile.com/b/zPIKrB/hfrAP)[10.3389/fpubh.2019.00039](http://dx.doi.org/10.3389/fpubh.2019.00039)

203. [Costa LF, Yokosawa J, Mantese OC, Oliveira TFM, Silveira HL, Nepomuceno LL, et al. Respiratory viruses in children younger than five years old with acute respiratory disease from 2001 to 2004 in Uberlândia, MG, Brazil. Mem Inst Oswaldo Cruz. 2006;101: 301–306. doi:](http://paperpile.com/b/zPIKrB/k2Xb)[10.1590/s0074-02762006000300014](http://dx.doi.org/10.1590/s0074-02762006000300014)

204. [Yorita KL, Holman RC, Steiner CA, Effler PV, Miyamura J, Forbes S, et al. Severe bronchiolitis and respiratory syncytial virus among young children in Hawaii. Pediatr Infect Dis J. 2007;26: 1081–1088. doi:](http://paperpile.com/b/zPIKrB/29BE)[10.1097/INF.0b013e31812e62c2](http://dx.doi.org/10.1097/INF.0b013e31812e62c2)

205. [Yu J, Liu C, Xiao Y, Xiang Z, Zhou H, Chen L, et al. Respiratory syncytial virus seasonality, Beijing, China, 2007-2015. Emerg Infect Dis. 2019;25: 1127–1135. doi:](http://paperpile.com/b/zPIKrB/5XvIE)[10.3201/eid2506.180532](http://dx.doi.org/10.3201/eid2506.180532)

206. [Yusuf S, Piedimonte G, Auais A, Demmler G, Krishnan S, Van Caeseele P, et al. The relationship of meteorological conditions to the epidemic activity of respiratory syncytial virus. Epidemiology and Infection. 2007. pp. 1077–1090. doi:](http://paperpile.com/b/zPIKrB/riwp)[10.1017/s095026880600776x](http://dx.doi.org/10.1017/s095026880600776x)

207. [Zhang Z-Y, Du L-N, Chen X, Zhao Y, Liu E-M, Yang X-Q, et al. Genetic variability of respiratory syncytial viruses (RSV) prevalent in Southwestern China from 2006 to 2009: emergence of subgroup B and A RSV as dominant strains. J Clin Microbiol. 2010;48: 1201–1207. doi:](http://paperpile.com/b/zPIKrB/6P2a)[10.1128/JCM.02258-09](http://dx.doi.org/10.1128/JCM.02258-09)

208. [Zhang X-L, Shao X-J, Wang J, Guo W-L. Temporal characteristics of respiratory syncytial virus infection in children and its correlation with climatic factors at a public pediatric hospital in Suzhou. J Clin Virol. 2013;58: 666–670. doi:](http://paperpile.com/b/zPIKrB/K43M)[10.1016/j.jcv.2013.09.027](http://dx.doi.org/10.1016/j.jcv.2013.09.027)

209. [Zhao H, Green H, Lackenby A, Donati M, Ellis J, Thompson C, et al. A new laboratory-based surveillance system (Respiratory DataMart System) for influenza and other respiratory viruses in England: results and experience from 2009 to 2012. Euro Surveill. 2014;19. doi:](http://paperpile.com/b/zPIKrB/rGdI)[10.2807/1560-7917.es2014.19.3.20680](http://dx.doi.org/10.2807/1560-7917.es2014.19.3.20680)

210. [Zlateva KT, Vijgen L, Dekeersmaeker N, Naranjo C, Van Ranst M. Subgroup prevalence and genotype circulation patterns of human respiratory syncytial virus in Belgium during ten successive epidemic seasons. J Clin Microbiol. 2007;45: 3022–3030. doi:](http://paperpile.com/b/zPIKrB/vjMv)[10.1128/JCM.00339-07](http://dx.doi.org/10.1128/JCM.00339-07)

211. [Waris M. Pattern of respiratory syncytial virus epidemics in Finland: two-year cycles with alternating prevalence of groups A and B. J Infect Dis. 1991;163: 464–469. doi:](http://paperpile.com/b/zPIKrB/aDK44)[10.1093/infdis/163.3.464](http://dx.doi.org/10.1093/infdis/163.3.464)

212. [Weigl JAI, Puppe W, Meyer CU, Berner R, Forster J, Schmitt HJ, et al. Ten years’ experience with year-round active surveillance of up to 19 respiratory pathogens in children. Eur J Pediatr. 2007;166: 957–966. doi:](http://paperpile.com/b/zPIKrB/G5GoC)[10.1007/s00431-007-0496-x](http://dx.doi.org/10.1007/s00431-007-0496-x)

213. U.S. department of health & human services (HHS). In: HHS.gov [Internet]. [cited 13 Mar 2023]. Available: <https://www.hhs.gov/>
